# Codon Usage Optimization in the Prokaryotic Tree of Life: How Synonymous Codons Are Differentially Selected in Sequence Domains with Different Expression Levels and Degrees of Conservation

**DOI:** 10.1128/mBio.00766-20

**Published:** 2020-07-21

**Authors:** José Luis López, Mauricio Javier Lozano, María Laura Fabre, Antonio Lagares

**Affiliations:** a Instituto de Biotecnología y Biología Molecular, CONICET, CCT-La Plata, Departamento de Ciencias Biológicas, Facultad de Ciencias Exactas, Universidad Nacional de La Plata, La Plata, Argentina; Université Claude Bernard Lyon 1; University of Vienna

**Keywords:** codon usage selection, mutational bias, genome evolution, core genes, singletons, translation efficiency, translation accuracy

## Abstract

Prokaryote genomes exhibit a wide range of GC contents and codon usages, both resulting from an interaction between mutational bias and natural selection. In order to investigate the basis underlying specific codon changes, we performed a comprehensive analysis of 29 different prokaryote families. The analysis of core gene sets with increasing ancestries in each family lineage revealed that the codon usages became progressively more adapted to the tRNA pools. While, as previously reported, highly expressed genes presented the most optimized codon usage, the singletons contained the less selectively favored codons. The results showed that usually codons with the highest translational adaptation were preferentially enriched. In agreement with previous reports, a C bias in 2- to 3-fold pyrimidine-ending codons, and a U bias in 4-fold codons occurred in all families, irrespective of the global genomic GC content. Furthermore, the U biases suggested that U_3_-mRNA–U_34_-tRNA interactions were responsible for a prominent codon optimization in both the most ancestral core and the highly expressed genes. A comparative analysis of sequences that encode conserved (*cr*) or variable (v*r*) translated products, with each one being under high (HEP) and low (LEP) expression levels, demonstrated that the efficiency was more relevant (by a factor of 2) than accuracy to modeling codon usage. Finally, analysis of the third position of codons (GC3) revealed that in genomes with global GC contents higher than 35 to 40%, selection favored a GC3 increase, whereas in genomes with very low GC contents, a decrease in GC3 occurred. A comprehensive final model is presented in which all patterns of codon usage variations are condensed in four distinct behavioral groups.

## Abstract (precis)

The prokaryotic genomes—the current heritage of the most ancient life forms on earth—are comprised of diverse gene sets, all characterized by varied origins, ancestries, and spatial-temporal expression patterns. Such genetic diversity has for a long time raised the question of how cells shape their coding strategies to optimize protein demands (i.e., product abundance) and accuracy (i.e., translation fidelity) through the use of the same genetic code in genomes with GC contents that range from less than 20 to more than 80%. Here, we present evidence on how codon usage is adjusted in the prokaryotic tree of life and on how specific biases have operated to improve translation. Through the use of proteome data, we characterized conserved and variable sequence domains in genes of either high or low expression level and quantitated the relative weight of efficiency and accuracy—as well as their interaction—in shaping codon usage in prokaryotes.

## INTRODUCTION

The wide range of GC contents exhibited by prokaryote genomes—i.e., from less than 20% to 80%—is believed to be primarily caused by interspecies differences in mutational processes that operate on both the coding and the noncoding regions (1–6). Since prokaryote genomes consist mainly of coding regions that tightly reflect the genomic GC content, mutational bias is a main force that shapes the codon usage of the majority of the genes (7, 8). Thus, understanding how selection is coupled to mutational processes to model codon usage under such diverse GC contents is an essential issue (9–11). Recent evidence suggests that prokaryotic genomes with intermediate to high GC contents are affected by mutations that are universally biased in favor of AT replacements (12, 13). That process is counterbalanced by selection-based constraints that, in turn, increase the GC content and fine-tune codon usage—i.e., the so-called mutation-selection-drift model (14–16). Intragenomic codon usage heterogeneities, however, are always present among different gene sets—i.e., between core genes that are shared throughout a given lineage and singletons (unique accessory genes) that are taxon and/or strain specific (17, 18). Furthermore, in a multipartite genome, the linkage between the physical patterns of heterogeneity in codon usage and the replicon location of the different core genes has also been recently demonstrated (19). The analysis of intragenomic codon usage heterogeneities by different authors (20, 21) has served to identify at least the following three distinctive gene groups. The first comprises the majority of the coding sequences that are associated with the so-called typical codon usage, while the second consists of the putative highly expressed (PHE) genes involving codon usages that are the best adapted to the translational machinery (20, 22–26). The third contains genes that encode the accessory information, including the singletons (unique genes) that are present in mobile genetic elements as well as in the most stable replicons (21, 27–30). The intracellular variations in codon usage can be explained on the basis of selective pressures that operate with different strengths depending on gene function and the resulting impact on cellular fitness (31). A search for the biochemical basis associated with the heterogeneity in codon usage among different gene sets has been the focus of numerous studies. Several lines of evidence have indicated that the biased codon usage in PHE genes correlates with the copy number of the specific tRNA species that decode the preferred codons (23, 32, 33) and with an optimal codon-anticodon interaction (34). The latter includes both the classical Watson-Crick interactions (WCIs) and a wobble base pairing with the corresponding cognate tRNAs. All these interactions have been taken into consideration in order to define different numerical indices (35, 36) as estimators of the codon adaptation to the existing tRNA pool. Though not considered in currently used translation-adaptation indices, evidence has also been found for other nonstandard codon-anticodon interactions which, by improving the decoding capacity, are also relevant to codon usage evolution (37–40).

The analysis of an extensive number of genes with different functions, degrees of ubiquitousness, and degrees of phylogenetic conservation has demonstrated that codon usage is related to gene expression level (32, 41, 42), the degree of conservation (18, 31, 43, 44), the genomic location—i.e., chromosome, chromid, or plasmidome (19, 45, 46)—and different features such as codon ramps and mRNA secondary structure, among others (47–49). Current evidence indicates that accessory genes involve atypical codon usages (21, 28, 46, 50) compared to the most conserved (ancestral) core genes in a given lineage. The latter genes, for their part, exhibit adaptational variations in codon usages ranging from typical to more biased, as the one observed in genes that correspond to highly abundant proteins which are coded by PHE genes (51). Moreover, that core genes may also exhibit remarkable codon usage heterogeneities has been recently demonstrated (19).

In the work reported here, after examining 29 different prokaryote families, we performed a consolidated analysis aimed at characterizing the specific intragenomic codon variations that lead to differences in codon usage between gene sets with diverse expression levels and degrees of conservation in a given lineage. The evaluation of intragenic regions with different coding characteristics—compared to strategies based on the global analyses of complete genes—enabled the recognition of different patterns of codon usages within a message to be translated. Thus, the questions emerged of (i) whether the codon usage patterns associated with highly expressed amino acid sequences (i.e., affecting efficiency) were the same as those associated with genes encoding highly conserved sequences (i.e., affecting accuracy) and (ii) whether the requirements for translation efficiency and accuracy were fully independent or whether those two types of demands interacted. The results have indicated how, even in organisms with quite different GC contents, alterations in specific codons are associated with a selective adaptation of the most ancestral genes compared to the adaptation of those genes that are newer in the phylogeny. Through an independent analysis of sequences associated with variable or conserved regions having different expression levels (i.e., low versus high), we were able to identify the specific codon usages associated with translation efficiency and accuracy as well as quantitatively estimate their relative relevance to codon usage.

## RESULTS

### Ancestry-dependent codon usage bias as revealed by the analysis of core genes from diverse prokaryotic families.

**FIG 1:**
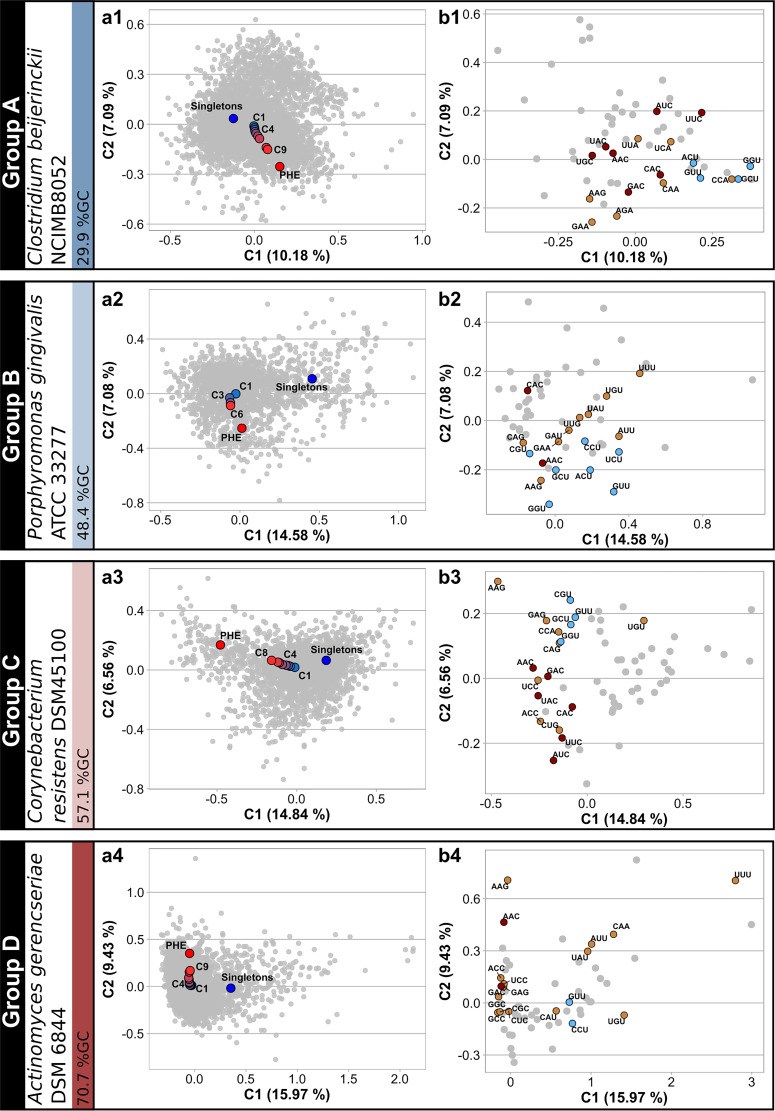
Raw-codon-count-based correspondence analysis (CA) plots of core-gene sets with different degrees of conservation throughout the phylogeny of selected prokaryote families (groups A to D). (a1 to a4) In 4 reference strains with different GC contents, individual genes (in gray) are represented in the space of the first-two CA components, with the percent variation of components 1 and 2 being indicated on the axes. CAs were computed using raw codon counts (RCC) as the input variables. Average coordinates (centroids) for different gene sets (i.e., singletons in blue, C1 to Cn in a gradient from blue to red, and putative highly expressed [PHE] genes in red) were projected on the CA space. In C1 to Cn, the higher number, the more ancestral the core gene set within the phylogeny. Table S1a (tab 1) lists the prokaryote species that were used to construct each Ci gene set by means of the EDGAR software (57, 58). (b1 to b4) Plots describing codon relative weight in the first two principal-component positions of the CA. Codons with the highest codon usage frequency (CUF) enrichment for each amino acid from C1 to PHE (i.e., those codons that better represent translational adaptation) are colored light brown, except when those same codons corresponded also to a 2-/3-fold C or to a 4-fold U bias, in which case they are colored dark brown and light blue, respectively.

López et al. (19) have recently demonstrated that in a model proteobacterium, the more ancestral the core genes were, the better adapted their codon usages were to the translational machinery. In order to investigate if such a correlation was associated with a general phenomenon in different prokaryote taxa, we assembled different core gene sets that progressed deeper into the phylogenies of 27 Gram-negative and -positive eubacterial families spanning the phyla *Proteobacteria*, *Actinobacteria*, *Firmicutes*, and *Bacteroidetes* along with 2 archaeal families from the phylum *Euryarchaeota*. Table S1a (tab 1) in the supplemental material itemizes for each taxon the number of genes in each gene set from the most recent core 1 (C1) to the most ancestral core n (Cn). In each prokaryote family, the most ancestral core gene set (Cn) consisted of 100 to 500 orthologs. The codon usage variation with gene ancestry within a given prokaryote family was evaluated through a correspondence analysis (CA) that used as variables the raw codon counts (RCC) of the individual genes in each genome analyzed (see Materials and Methods). The average values of the first two components for the core gene sets C1 to Cn were projected on the CA plots. Figure 1 (left graphs) depicts the CA for four genomes specifically selected to represent groups of organisms with different types of CA plots and GC3 contents in their core genes, PHE genes, and singletons, namely, groups A to D (see Materials and Methods). CA were also calculated using relative synonymous-codon usages (RSCUs) as input variables instead of RCC as presented in Fig. S1A. In agreement with a recent study with Sinorhizobium meliloti (19), in all instances a directional shift in the codon usage positions was evident from the most recent toward the most ancestral core gene set. That this ancestry-dependent pattern of codon usage variation was observed in even quite distant prokaryote families among those analyzed in this study was remarkable (cf. the CA plots for all other species in Fig. S1B, left graphs). In the evolution of core codon usages, however, the extent of the observed shifts and the type of synonymous codons enriched in each taxon (i.e., the direction of change) varied markedly among different families (Fig. 1, Fig. S1A, and Fig. S1B, right graphs).

10.1128/mBio.00766-20.1 FIG S1 (A) RSCU-based correspondence analysis (CA) plots of core gene sets with different degrees of conservation throughout the phylogeny of selected prokaryote families (groups A to D). (a1 to a4) In 4 reference strains with different GC contents, individual genes (in gray) are represented in the space of the first two CA components, with the percent variation of components 1 and 2 being indicated on the axes. CA were computed using RSCUs as the input variables. Modal codon usages―calculated as indicated in Materials and Methods in the main text―for different gene sets (i.e., singletons in blue, C1 to Cn in a gradient from blue to red, and PHE in red) were projected on the CA space. In C1 to Cn the higher number indicates a more ancestral core gene set within the phylogeny. (b1 to b4) Plots describing codon relative weight in the first two principal-component positions of the CA. Codons that are colored in brown indicate those which present―for each amino acid―the highest CUF enrichment from C1 to PHE (i.e., those codons that better represent translational adaptation) except when some of those codons also correspond to a C bias (colored in red) or a U bias (colored in light blue). (B) CA plots of core gene sets with different degrees of conservation throughout the phylogeny of 25 prokaryote families. The strains that were not included in the main article are represented here and classified as belonging to group A, B, C, or D. (a1 to a25) The individual genes (in gray) are represented in the space of the first two CA components, with the percent variation of components 1 and 2 being indicated on the axes. CAs were computed using either raw codon counts (RCC) or RSCU as the input variables as indicated. Average coordinates (centroids) for different gene sets (i.e., singletons in blue, C1 to Cn in a gradient from blue to red, and PHE in red) were projected on the CA space in the RCC-based CA plots. Similarly, modal codon usages for the same gene sets―calculated as indicated in Materials and Methods―were projected on the RSCU-based CA plots. In C1 to Cn, the higher number indicate a more ancestral core-gene set within the phylogeny. Table S1a (tab 1) lists the prokaryote species that were used to construct each Ci gene set by means of the EDGAR software (J. Blom, S. P. Albaum, D. Doppmeier, A. Pühler, et al., BMC Bioinformatics 10:154, 2009, https://doi.org/10.1186/1471-2105-10-154; J. Yu, J. Blom, S. P. Glaeser, S. Jaenicke, et al., J Biotechnol 261:2–9, 2017, https://doi.org/10.1016/j.jbiotec.2017.07.010). (b1 to b25) Plots describing codon relative weight in the first two principal-component positions of the CA. Codons that are colored brown indicate those which present―for each amino acid―the highest CUF enrichment from C1 to PHE (i.e., those codons that better represent translational adaptation) except when some of those codons also correspond to a C bias (colored red) or a U bias (colored light blue). Download FIG S1, PDF file, 2.9 MB. Copyright © 2020 López et al. 2020 López et al. This content is distributed under the terms of the Creative Commons Attribution 4.0 International license.

10.1128/mBio.00766-20.6 TABLE S1a Tab 1, bacterial strains used in this work and their corresponding core genes; tab 2, tree distances of the core genes extracted from each reference phylogenetic tree shown in the next tabs; all other tabs, phylogenetic trees of prokaryote families used in this study. Download Table S1a, XLSX file, 2.3 MB. Copyright © 2020 López et al. 2020 López et al. This content is distributed under the terms of the Creative Commons Attribution 4.0 International license.

The general features that characterized the bias in codon usages can be summarized as follows. First, a general pattern indicated that in bacteria from groups B, C, and D, the PHE genes are enriched in codons with higher GC3 than in singletons (Fig. 1 and Fig. S1, right graphs). Conversely, an AU enrichment in the third position of codons was observed in the ancestral core fractions of organisms from group A which have extremely low GC contents. Second, from C1 to Cn in the CA plot, the codon usages gradually shifted away from the position of the singletons (the unique genes) to approach the region where the PHE genes were located (Fig. 1 and Fig. S1, left graphs). Similar results were obtained when PHE genes were subtracted from the different Cn cores (i.e., Cn-woPHE [Fig. S2]). Thus, the overall evidence suggested that gene ancestry correlated with a codon usage optimization that resembled the one observed in the PHE genes. Nonetheless, the most ancestral core genes (i.e., the Cn gene sets) never overlapped with the position of the PHE genes in the CA plots. In most prokaryote species, the order of positions in the CA plot followed the sequence singletons-C1-Cn, which series was associated with both an enrichment in C-ending 2-/3-fold degenerate codon families (i.e., a C bias in the 2-/3-fold degenerate pyrimidine-ending codons) (Fig. S3A, panel a, shows a significant C bias from the most recent to the most ancestral core gene set―both without PHE genes―with a *P* value of <0.02 [*t* test]) and an additional enrichment in U-ending 4-fold degenerate codon families (Fig. S3A, panel b, shows a significant U bias associated with gene ancestry, with a *P* value of <0.05 [*t* test]). Such C and U biases were found to be even more intense when comparing the most ancestral core gene sets against PHE genes (Fig. S3B). Each of the previous effects varied in relative intensity among the different prokaryote families and was more intense in microorganisms from groups B and C (central blue and orange bars in Fig. S3 [all panels]). In agreement with previous reports (52), no specific C bias with increasing core gene ancestry was observed in the TGC codon (Cys) irrespective of the group under consideration. Comparable codon enrichments also were found when comparing Cn-woPHE genes (i.e., Cn without PHE genes) to PHE genes (Fig. S3B, panels a and b, where a significant C bias [*P < *0.002, *t* test], except for group D, and a significant U bias [*P < *0.03, *t* test] were observed). Wald et al. (40) have previously reported that the C and the U biases are associated with an improved codon usage correspondence to the anticodons of the tRNA pool. The combined effects of the C and U biases are the basis for the “rabbit head” distribution of genes that can be observed in most of the CA plots (gray dots), an effect that was originally described for Escherichia coli (21). Contrasting with the codon usage of core and PHE genes, the singleton genes tend to be enriched in A/U-ending codons.

10.1128/mBio.00766-20.2 FIG S2 RCC-based CA plots of core gene sets excluding the PHE genes. (a1 to a4) PHE genes were extracted from each group of core genes and the resulting gene sets, indicated as Ci (from “1” to “n,” in blue to red circles), were projected on the CA plots. Singletons and PHE genes are in blue and red, respectively. (b1 to b4) Plots describing codon relative weight in the first two principal-component positions of the CA. Codons with the highest CUF enrichment for each amino acid from C1 to PHE (i.e., those codons that better represent translational adaptation) are colored light brown, except when those same codons corresponded also to a 2-/3-fold C bias or to a 4-fold U bias, in which cases they are colored dark brown and light blue, respectively. Such C bias and U bias indicate that 2-/3-fold degenerate amino acids are biased towards the use of C-ending codon, and that 4-fold degenerate amino acids are biased towards the use of U-ending codon, respectively. Download FIG S2, PDF file, 0.5 MB. Copyright © 2020 López et al. 2020 López et al. This content is distributed under the terms of the Creative Commons Attribution 4.0 International license.

10.1128/mBio.00766-20.3 FIG S3 (A) Ancestry-dependent C and U biases in the codon usage in the gene sets C1-woPHE and Cn-woPHE (i.e., Cn without PHE genes) in organisms from groups A to D. (a) C bias at the third position of codons in 2-/3-fold degenerate amino acids (significant bias in all groups at *P *value of <0.02, *t* test). (b) U bias at the third position of codons in 4-fold degenerate amino acids (significant bias in all groups at *P *value of <0.05, *t* test). Average values and standard deviations (SD) are shown in each panel on the right side considering all codons together (except TGC for Cys in panel a). (B) Ancestry-dependent C and U biases in the codon usage in the gene sets Cn-woPHE (i.e., Cn without PHE genes) and PHE genes in organisms from groups A to D. (a) C bias at the third position of codons in 2-/3-fold degenerate amino acids (significant bias in groups A to C at *P *value of <0.002, *t* test). (b) U bias at the third position of codons in 4-fold degenerate amino acids (significant bias in all groups at *P *value of <0.03, *t* test). Average values and standard deviations (SD) are shown in each panel on the right side considering all codons together (except TGC for Cys in panel a). Download FIG S3, PDF file, 0.4 MB. Copyright © 2020 López et al. 2020 López et al. This content is distributed under the terms of the Creative Commons Attribution 4.0 International license.

### Indication from m-tAI values that the codon usages of the most ancestral genes are better adapted to the cellular translational machinery.

**FIG 2:**
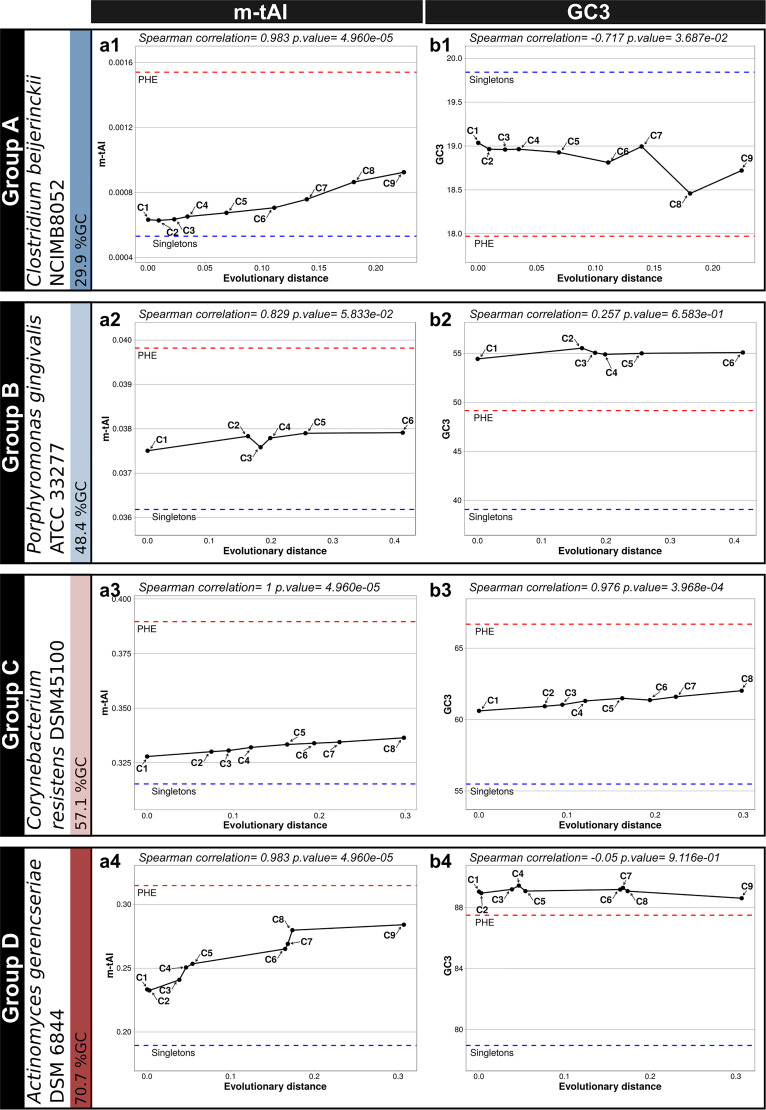
Codon usage adaptations to the cellular tRNA pool, and changes in the GC3 content of different prokaryote core genes. The reference strains represented are the same five as in Fig. 1. (a1 to a4) In each panel, the modal tRNA adaptation index (m-tAI) calculated for each of the Ci gene sets as described in Materials and Methods is plotted on the ordinate as a function of the evolutionary distance indicated on the abscissa (Table S1a, tab 2) as inferred from the corresponding phylogenetic trees included in Table S1a to c. Higher values of m-tAI indicate an enrichment in the codon usage frequencies of those synonymous codons better adapted to the host cell tRNA pool. The C1 to Cn gene sets plotted are the same as those presented in Fig. 1. The red and blue horizontal dashed lines correspond to the respective m-tAI values calculated for the PHE genes and the singletons. (b1 to b4) In each panel, the average GC3 content in each core gene set of increasing ancestry is plotted on the ordinate as a function of the evolutionary distance indicated on the abscissa as in panels a1 to a4. The PHE genes and the singletons are represented as red and blue horizontal dashed lines, respectively.

In order to explore how extensive the correlations between codon usage, gene ancestry, and translation efficiency were, we calculated the modal species-specific tRNA-adaptation index (m-tAI) values for the C1 to the Cn genes for a given strain and used those indices to estimate the adaptation of each gene set to the tRNA pool. Each m-tAI takes into consideration both the copy number of each tRNA structural gene as an estimation of that tRNA’s cellular concentration and the codon-anticodon interactions, including the classical Watson-Crick interactions (WCIs) along with the wobble rules (see Materials and Methods). Figure 2 and Fig. S4, left graphs, illustrate how with progressive gene ancestry the m-tAI generally increases to often approach that of the PHE genes, thus evidencing that the most ancestral cores are enriched in genes that displayed adaptive—i.e., selection-dependent—changes in their codon usage. That such m-tAI increases with progressive ancestry had been observed in strains from group A (average Spearman coefficient = 0.99 and *P* value = 0.002), group B (average Spearman coefficient = 0.66 and *P* value = 0.02), and group C (average Spearman coefficient 0.90 and *P* value = 0.08) was indeed remarkable (cf. Fig. 2 and Fig. S4, left graphs). Unfortunately, nonstandard forms of base pairing, such as U:U interactions and others, are not included in the m-tAI calculations, and this fact might negatively impact the way that the m-tAi varies with ancestry, in particular in organisms from the GC-rich group D. In the reference strains from these prokaryote families, the PHE genes (red dashed lines) were always associated with higher m-tAI values than those of the core gene sets from the same genome. Conversely, singletons (blue dashed lines) were always the gene sets with the lower m-tAIs, suggesting that accessory genes (i.e., those present in plasmids and phages and the unique genes in chromosomes) involve codon usages that—most likely due to their nonessential character—are far from being optimized with respect to the host translation machinery. Strains with the characteristics described above have genomes with quite diverse GC contents, ranging from ca. 30% to more than 70%. Exceptions to the general increase in the m-tAI values with ancestry are likely due to m-tAI deficiencies to quantitate nonstandard codon-anticodon interactions (i.e., those different from WCIs, along with wobble base pairing) (35).

10.1128/mBio.00766-20.4 FIG S4 Codon usage adaptations to the cellular tRNA pool and changes in the GC3 content of core gene sets with different degrees of conservation throughout the phylogeny of 25 prokaryote families. The strains represented here are the same as the 25 in Fig. S1. (a1 to a25) In each graph, the modal tRNA-adaptation index (m-tAI) calculated for each of the Ci gene sets as described in Materials and Methods is plotted on the ordinate as a function of the evolutionary distance (Table S1a, tab 2) indicated on the abscissa as inferred from the corresponding phylogenetic trees included in Table S1a to c. Higher values of m-tAI indicate an enrichment in the codon usage frequencies of those synonymous codons better adapted to the host-cell tRNA pool. The C1 to Cn gene sets plotted are the same as those presented in Fig. 1. The red and blue horizontal dashed lines correspond to the respective m-tAI values calculated for the PHE genes and the singletons. (b1 to b25) In each graph, the average GC3 content in each core gene set of increasing ancestry is plotted on the ordinate as a function of the evolutionary distance indicated on the abscissa as in panels a1 to a25. The PHE genes and the singletons are represented as red and blue horizontal dashed lines, respectively. Download FIG S4, PDF file, 2.9 MB. Copyright © 2020 López et al. 2020 López et al. This content is distributed under the terms of the Creative Commons Attribution 4.0 International license.

### Effect of codon optimization on the GC content.

An analysis of the prokaryote genomes with different GC contents enabled us to explore how the GC composition at the third base of codons (i.e., the GC3) changed in the core gene sets over ancestry and to compare the results with the GC3 in PHE genes and singletons. Since the first two positions in codons are constrained by the protein-coding information, most of the GC changes result in variations in synonymous codons (2). As we have seen in the two previous sections, core genes adjust their codon usages in the direction of the PHE genes (Fig. 1 and Fig. S1, left graphs) in order to improve translation (Fig. 2 and Fig. S4, left graphs). The question thus was raised as to how bacteria with different GC contents changed their GC3 compositions in the process of adapting their codon usages. The results presented in Fig. 2 and Fig. S4 (right graphs) show that changes in GC3 in genomes from groups A to D each follow a distinctive pattern as determined by comparing singletons to Ci-Cn to PHE genes. Whereas in genomes that belong to group A (overall GC content lower than ca. 35%) the GC3 decreases from singletons to Ci to PHE genes (cf. Fig. 2, panel b1), in the genomes included in group C the GC3 either increases from singletons to Ci to PHE (cf. Fig. 2, panel b3) or plateaus in Ci to PHE genes at a high level (cf. Fig. S4, panel b17). In contrast, genomes pertaining to group B exhibited a biphasic pattern with an initial GC3 increase from the level of the singletons up to the contents of the Ci series (with i varying from 1 to n) followed by a later decrease from the Cn values down to those of the PHE genes (cf. Fig. 2, panel b2). Those changes in the group B genomes were reflected in pronounced forward and backward movements in the position of the core genes in the CA plots, first from singletons to Ci and then from Cn to the PHE genes (cf. Fig. S1, organisms in group B). A similar biphasic pattern in the CA plots could also be recognized, though softened, in certain species that were included in group C or even group D, in which the PHE genes did not evidence a decrease in GC3 levels compared to those of the core genes. The genomes in group D had extremely high global GC contents and had GC3 values in all their core gene sets (C1 to Cn) that were comparable to—though slightly higher—than the corresponding values in their PHE genes. Next, we describe how individual codons for a given amino acid are selected in the most ancestral core gene sets.

### Characterization of codons that improve adaptation to the tRNA pool.

**FIG 3:**
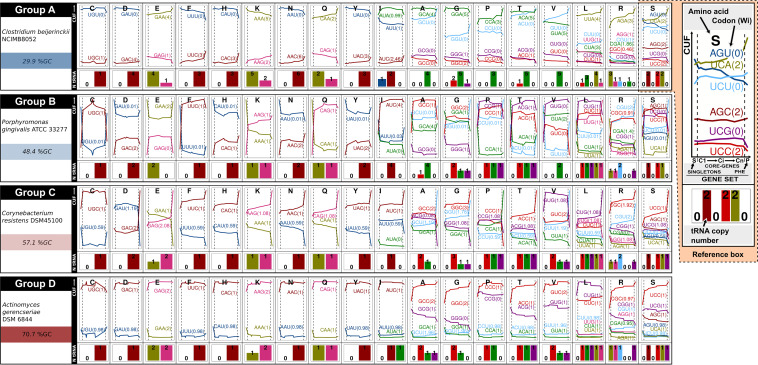
Codon usage frequencies and absolute adaptiveness values (*Wi*s) of the gene sets analyzed in this work, together with the tRNA gene copy numbers for strains of the four reference groups A to D. For the amino acid indicated by the corresponding single-letter identification code located above each panel, the change in the modal CUFs (see Materials and Methods) of the core gene sets with increasing ancestries (left to right, C1 to Cn), the PHE genes, and the singletons are plotted in the upper portions as solid horizontal curves for each of the indicated codon triplets between the two vertical broken lines, for the singletons to the left of the first of those lines, and for the PHE genes to the right of the second (with singletons and PHE genes being located at the beginning and the end of the curves, respectively). The CUFs are represented by different colors, with the associated absolute adaptiveness value (*Wi* [35]) being indicated within parentheses beside each triplet. Finally, the presence and gene copy number of the cognate tRNA species of a given synonymous codon bearing the exact complementary anticodon is depicted with a number and a bar of proportional height in the lower panel in the same color as the corresponding triplet and curve in the upper portion.

The variations in the use of individual codons when progressing from the C1 to the Cn gene sets were analyzed in the different prokaryote genomes, together with the tRNA gene copy numbers and the absolute adaptiveness values (*Wi*s; see Materials and Methods). Figure 3 and Fig. S5A illustrate the codon usage frequencies (CUFs; see Materials and Methods) for singletons, PHE genes, and core genes with increasing ancestry together with the tRNA gene copies and the *Wi*s (Fig. S5B summarizes the *Wi*s, ΔCn-C1, and ΔPHE-Cn in the different genomes studied). In agreement with previous reports (10), our results demonstrated that the CUFs among synonymous codons were strongly influenced by the global GC content in each genome—i.e., codons with G and C in the 3′ position (N_3_) were the most abundant synonymous codons in the GC-rich genomes, whereas A and U were predominant in that position in the genomes with low GC contents (Fig. 3 and Fig. S5A). An inspection of the proportion of codon usage for each amino acid in ancestral cores compared to the most recent ones (curves in Fig. 3 and Fig. S5A and B) revealed that in most genomes a C-bias enrichment occurred with increased ancestry at the 3′ position of the 2-fold pyrimidine-ending codons—for Asp (GAC), Phe (UUC), His (CAC), Asn (AAC), and Tyr (UAC)—as well as in the unique 3-fold codons for Ile (AUC). Corresponding to the observed C bias, in all these examples high *Wi*s (shown in parentheses in the figure) were observed for the C-ending codons, which triplets were decoded through exact WCIs with the cognate tRNA species (i.e., with the anticodon G_34_N_35_N_36_). Because of the absence of tRNA species bearing anticodons A_34_N_35_N_36_ for these five amino acids, lower *Wi*s were obtained for the U-ending codons as the consequence of a weaker wobble codon-anticodon non-WCI recognition. Especially noteworthy was the observation that, though to a lesser extent, the bacteria with extremely low GC contents likewise exhibited a C bias in the 2- to 3-fold codon family, irrespective of a global decrease in the GC3 value, as in the example of Clostridium beijerinckii (cf. Fig. 2 and 3).

10.1128/mBio.00766-20.5 FIG S5 (A) Codon usage frequencies and adaptation indices (*Wi*s) of the gene sets analyzed in this work, together with the tRNA gene copy numbers for strains of 25 different prokaryote families included in groups A to D. For the amino acid indicated by the corresponding single-letter identification code located above each graph, the change in the codon usage frequencies (CUFs; see Materials and Methods) of the core gene sets with increasing ancestries (left to right, C1 to Cn), the PHE genes, and the singletons are plotted in the upper portions as solid horizontal curves for each of the indicated codon triplets between the two vertical broken lines, for the singletons to the left of the first of those lines, and for the PHE genes to the right of the second (with singletons and PHE genes being located at the beginning and the end of the curves, respectively). The CUFs are represented by different colors with the associated codon adaptation index being denoted within parentheses beside each triplet. Finally, the presence and gene copy number of the cognate tRNA species of a given synonymous codon bearing the exact complementary anticodon is depicted with a number and a bar of proportional height in the lower portion in the same color as the corresponding triplet and curve in the upper portion. (B) General codon enrichment profiles in reference strains of the 29 prokaryote families analyzed. The reference species from groups A, B, C, and D are indicated. The enrichment in a given ith codon over ancestry was calculated as CODON“i” CUFCn–CODON“i” CUFC1, referred to simply as Δ(Cn-C1). In like manner, the enrichment in the PHE genes over ancestry was calculated as CODON“i” CUFPHE–CODON“i” CUFCn, similarly referred to as Δ(PHE-Cn). The rectangular boxes to the left indicating the codons in the different amino acids are color-coded according to the nature of the 3′ base present as follows: blue, variants U; red, variants C; green variants A; and violet, variants G. The vertical rectangles at the left of each small panel represent the *Wi*s, with the intensity of the blue color being proportional to the values for each codon. The *Wi* color intensities are furthermore normalized to the maximum *Wi* value in each amino acid codon family. The vertical rectangles in the middle and at the right of each small panel represent the Δ(Cn-C1) or Δ(PHE-Cn), with the color intensity being in proportion to either an increased (blue), a decreased (red), or an equal (white) CUF in the gene sets under analysis (Cn versus C1, PHE versus Cn), as indicated in the color keys. The variations in codon-anticodon interactions (WCI, wobble base, U:U) along with their participation in the C (red) and U biases (light blue) are illustrated in the boxes at the bottom. Download FIG S5, PDF file, 1.8 MB. Copyright © 2020 López et al. 2020 López et al. This content is distributed under the terms of the Creative Commons Attribution 4.0 International license.

In the instance of the 2-fold purine-ending codons—that is, GAA and GAG for Glu, AAA and AAG for Lys, and CAA and CAG for Gln—we observed that the codons with G or A in the 3′ position were enriched from C1 to Cn and from Cn to PHE genes (i.e., ΔCn-C1 and ΔPHE-Cn in Fig. S5B, respectively) depending upon which tRNA species (anticodons) were present. In those examples where only the tRNAs bearing the U_34_N_35_N_36_ anticodons were present, the cognate A-ending codons recognized by WCIs were the ones that became enriched in the most ancestral core and/or PHE genes (cf. in Fig. S5B the GAA triplet for Glu in Chromobacterium violaceum, Paenibacillus graminis, Bacillus subtilis, Bordetella holmesii, and Leisingera methylohalidivorans, the AAA for Lys in Methanobrevibacter smithii and Bacillus subtilis, and the CAA for Gln in M. smithii, Streptococcus equi, and B. subtilis). Accordingly, these 3′ A-ending codons were associated with higher *Wi*s than the corresponding codons ending in G, as the latter were recognized only by wobble-base pairing (i.e., G_3_-U_34_ interaction). In a second circumstance, where both tRNA species for the same amino acid (i.e., those bearing anticodon U_34_N_35_N_36_ or C_34_N_35_N_36_) were present, a more frequent enrichment in G-ending codons was observed (with few exceptions) since such codons can be decoded by either Watson-Crick or wobble interactions with the tRNA anticodon C_34_N_35_N_36_ or U_34_N_35_N_36_, respectively. In those few examples where the A-ending codons were more enriched than the G-ending codons, a higher copy number of the tRNA genes was always observed with anticodon U_34_N_35_N_36_ than that obtained with the anticodon C_34_N_35_N_36_ (cf. in Fig. 3 and Fig. S5A and B the GAA triplets for Glu in Bacteroides vulgatus and C. beijerinckii, the AAA triplets for Lys in Sulfurospirillum multivorans, and the CAA triplets for Gln in C. beijerinckii and S. multivorans).

A different codon usage bias—in a pattern not found in the 2-/3-fold degenerate amino acids—was observed in codons encoded by 4-fold degenerate amino acids (Val, Thr, Pro, Gly, and Ala) or by the 4-fold boxes of the 6-fold degenerate amino acids (Ser, Leu, and Arg). In these 4-fold groups, a U-bias enrichment (i.e., an NNU codon enrichment) was observed in the PHE genes from most of the genomes irrespective of their GC contents (Fig. 3 and Fig. S5A and B). This enrichment in U-ending codons, previously reported as a U bias (40), could not be explained by WCIs with A_34_N_35_N_36_ tRNAs because the latter species are not present in prokaryotes, except in the case of Arg. The observed U bias likely occurred through the previously proposed nonconventional codon-U_3_–anticodon-U_34_ interaction that was known to exist in bacteria (53). The presence of U_34_N_35_N_36_ tRNA species might, then, lead to an increase in both NNA and NNU codons as a consequence of positive WCIs and U_3_-U_34_ interactions, respectively.

All the codon adaptations that we have described in this section referring to core genes proved to be more prominent in the PHE genes, whose triplets were even better adapted to the translational machinery. Contrasting with such a strong pattern of selection-associated codon bias, the singletons displayed codon usages that were in general the most distant from those observed in the PHE genes (as exemplified in the CUFs in Fig. 3 and Fig. S5A and in the CA plots from Fig. 1 and Fig. S1). These observations are also in agreement with variations in the m-tAIs for the different gene sets presented in the previous section.

### Search for coding signatures for translation efficiency and accuracy: codon usage profiles associated with sequences encoding HEP_*vr* and HEP_*cr* translated domains.

**FIG 4:**
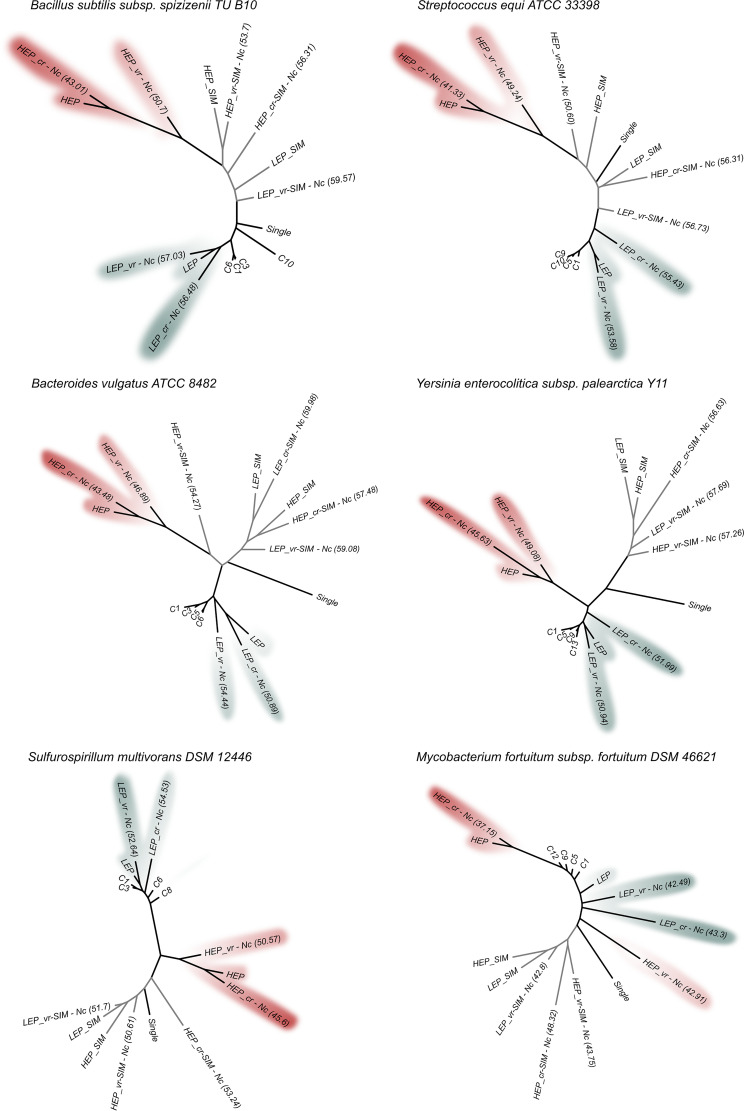
Neighbor-joining distance trees of different gene sets encoding HEP, LEP, and their associated conserved (*cr*) and variable (*vr*) regions based on the corresponding modal codon usage. Modal codon usage-based neighbor-joining trees with black branches were constructed for the indicated natural gene sets and their intragenic regions (*cr* and *vr*) following the method described by Karberg et al. (17) along with the neighbor-joining program of the Phylip package (62). Artificially simulated sequences were used as controls in the neighbor-joining tree (SIM labeled data and gray branches in the tree). Such artificially generated sequences were evolved under a model with no pressure for codon selection and preserving the same *K_A_*/*K_S_* ratio as that corresponding to each of their natural HEP/LEP set of homologs (see Materials and Methods). LEP_*cr*-SIM sequences are not included since, on average, fewer than 53 conserved amino acid positions/protein were collected in the simulation. Phylogenetic trees were drawn through the use of the Figtree application (59). Abbreviations: C1 to Ci, core gene sets with increasing ancestry; single, singletons; HEP, genes encoding proteins with the highest expression level; LEP, genes encoding proteins with the lowest expression level; HEP_*cr*, conserved HEP sequences (dark red); HEP_*vr*, variable HEP sequences (light red); LEP_*cr*, conserved LEP sequences (dark blue); and LEP_*vr*, variable LEP sequences (light blue). HEP and LEP *cr* and *vr* subfractions were recovered as indicated in Materials and Methods through the use of the polypeptide sequences included in C13 for Yersinia enterocolitica subsp. *palearctica* Y11, C10 for Streptococcus equi ATCC 33398, C8 for Sulfurospirillum multivorans DSM 12446, C9 for Bacillus subtilis subsp. *spizizenii* TU B 10, C6 for Bacteroides vulgatus ATCC 8482, and C12 for Mycobacterium fortuitum subsp. *fortuitum* DSM 46621 (ATCC 6841). The effective number of codons (Nc_s_) as previously defined by Wright (71) are indicated in parentheses for the *cr* and *vr* subset of sequences.

Expression level and amino acid sequence conservation are both parameters that positively correlate with codon usage optimization (54). Nevertheless, the relative relevance of efficiency and accuracy to translation and the way in which either one of those parameters affects the other have not yet been investigated in detail. A central limitation that made such studies difficult was associated with the natural genomic heterogeneity in gene ancestry along with the expression level and the sequence conservation (structural constraints) in the translated products. In order to reduce the degrees of freedom in the analysis, for each of six different bacterial species, we created two distinct gene sets based on the experimental proteome data. One of those gene sets consisted of genes encoding proteins with the highest expression levels in the cell (i.e., the HEP), while the other was associated with proteins with low cellular abundance (i.e., the LEP). Then, the conserved (*cr*) and variable (*vr*) sequences among the orthologs were collected from each individual gene (see Materials and Methods), and the corresponding highly expressed conserved (HEP_*cr*), highly expressed variable (HEP_*vr*), lowly expressed conserved (LEP_*cr*), and lowly expressed variable (LEP_*vr)* modal codon usages were used to calculate the relative distances illustrated in the neighbor-joining tree presented in Fig. 4. In five out of the six species present in the trees (all except *Mycobacterium fortuitum*), the HEP_*cr* and HEP_*vr* sequences separated from those of the singletons, the core genes, and all the LEPs as a result of a strong codon usage adaptation (also reflected in the low effective number of codons [Nc_s_] associated with the HEPs, indicated in parentheses following labels in the tree). Furthermore, the large distance in the tree between HEP_*cr* and LEP_*cr* (where both sequences encode regions with conserved amino acids but with different expression levels) compared to the much shorter distance between HEP_*cr* and HEP_*vr* (where both encode highly expressed products with different degrees of conservation) pointed to the quantitatively stronger effect of efficiency over accuracy in shaping codon usage bias. Control data sets were incorporated into the trees in Fig. 4 (branches in gray) using artificially evolved sequences with no pressure for codon selection (see Materials and Methods). The results show that, as expected, for the six analyzed genera the distance between the most and the least selected gene sets (i.e., distance from HEP_*cr* to LEP_*vr*) was always larger in natural genes than in the simulated sequences without selection (i.e., [distance from HEP_*cr* to LEP_*vr*]^natural^/[distance from HEP_*cr* to LEP_*vr*]^simulated^ > 1, with an average value ± standard deviation [SD] = 1,69 ± 0.20). Thus, natural sequences display more divergent (positively adapted) codon usages. It is noteworthy that SIM sequences tended to group with singletons―the least adapted gene set―and had in general higher Nc values than their corresponding natural sequences.

**FIG 5:**
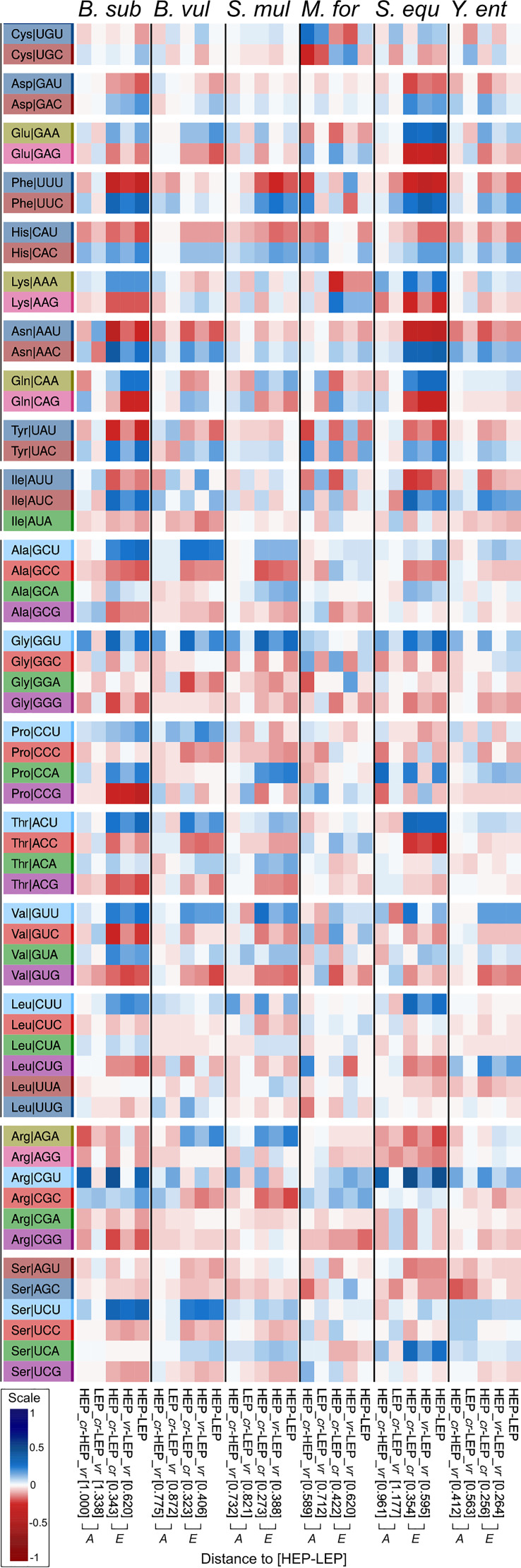
Heat map representation expressing differences in modal codon usage profiles between the indicated gene sets. The color scale from red to blue indicates the relative level of use of each particular codon in a gene set compared to that of another (i.e., gene set 1 versus gene set 2). The blue color corresponds to the dominant use of a particular codon in gene set 1 over the use of the same codon in gene set 2 (and vice versa for the red color). Amino acids are indicated in the standard three-letter code. The heat map was constructed through the use of the phytools R package (72). Distance between gene sets was determined using their corresponding modal codon usages as previously reported (46). “HEP (gene set 1)–LEP (gene set 2)” represents the profile of the optimized codons when comparing the coding strategies in high- versus low-expression genes (i.e., reflecting differences in their modal codon usages). The columns indicated by “*A*” correspond to the profiles of codons optimized as a result of accuracy (i.e., differences between HEP_*cr*–HEP_*vr* and LEP_*cr*–LEP_*vr*). The columns indicated by “*E*” correspond to the profiles of optimized codons through high expression (i.e., reflecting differences in efficiency between HEP_cr–LEP_*cr* and HEP_*vr*–LEP_*vr*). The numbers in brackets indicate the extent to which changes induced by either efficiency or accuracy approach the differences in codon usage between HEP and LEP (i.e., distances from each column to the column HEP–LEP). The shorter the distance in brackets the stronger the evolutionary constraint and the contribution of the indicated factor (i.e., accuracy or expression level) to codon optimization.

**FIG 6:**
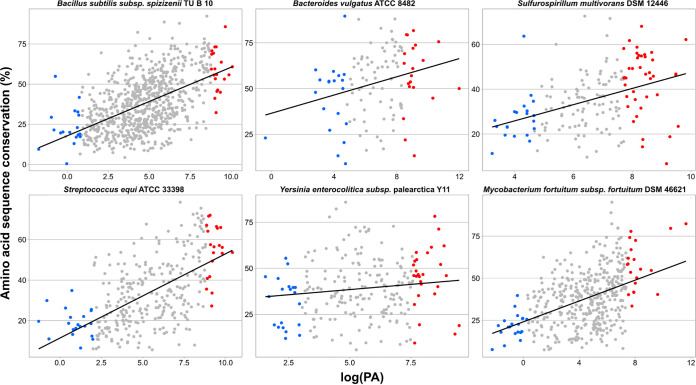
Amino acid sequence conservation in proteins with different cellular abundances. The amino acid sequence conservation calculated for proteins of the indicated bacterial species and core fractions (see Materials and Methods) are plotted on the ordinate as a function of the logarithm of the corresponding protein abundance (logPA) on the abscissa. The red and blue dots correspond to HEP and LEP, respectively, with all the other proteins of the same core represented in gray. The linear regressions and graphs were all made with the ggplot2 library from the R package.

Codons that were optimized as a result of accuracy under high and under low expression—i.e., HEP_*cr*–HEP_*vr* and LEP_*cr*–LEP_*vr*, respectively, labeled “*A*” for “accuracy” at the bottom of Fig. 5—were highly coincident with the codons that were optimized through efficiency—i.e., HEP_*cr*–LEP_*cr* and HEP_*vr*–LEP_*vr*, labeled “*E*” for “efficiency.” For some organisms, the greater distance between HEP_*cr* and HEP_*vr* than between LEP_*cr* and LEP_*vr* (Fig. 4) indicates a stronger influence of accuracy in codon usage optimization when operating under high-expression conditions, thus pointing to an interaction between the simultaneous requirements of high fidelity and efficiency. The most relevant contributions to the global difference in codon usage between HEP and LEP were efficiency (both in conserved and in variable regions) (*E* columns in Fig. 5) followed by accuracy under high expression (first *A* column in Fig. 5) (the stronger the contribution of each factor, either *E* or *A*, the shorter the distance in brackets to HEP-LEP in the figure). The heat maps display the complete profiles of preferred codons for sequences requiring high translational accuracy and/or efficiency (protein demands). As expected, the preferred codon for each amino was in agreement with the C and U bias and the tRNA copy number described in the previous sections. In light of these results, the highly expressed variable and conserved domains constitute the basis for explaining the observed codon usage optimization in the most ancestral core gene sets (Cn), which concentrate HEPs (Table S3). Figure 6 illustrates that HEP sequences (red dots) are those under the highest selective pressure to optimize codon usage because of both their expression level and their degree of conservation.

## DISCUSSION

Since gene adaptation to a host cell is expected to be associated with an improved codon selection for translation efficiency and accuracy (42, 55), we investigated correlations between core gene ancestry and their modal codon usage within a given prokaryote family. In order to ascertain if the adaptation of the most ancestral core genes was an extensive phenomenon among prokaryotes, we analyzed core modal codon usages in 27 different species of *Bacteria* and 2 of *Archaea*. That in the CA plots the most ancestral core genes had been the ones with the closest location to the PHE genes in all families was remarkable and strongly indicated a core codon usage adaptation that likely operated to improve translation. In agreement with the position of the different gene sets in the CA plots, the m-tAI values served to confirm that the PHE genes were the best-adapted gene set, followed by the Cn to C1 core genes, in that order, and finally by the singletons, with those being the least adapted genes with the lowest m-tAIs in the genome. Studies by others have previously demonstrated that the level of gene expression together with the need to preserve accuracy during the translation of conserved amino acid regions are both among the main parameters that govern codon usage selection (54). The bioinformatics isolation of conserved (*cr*) and variable (*vr*) coding sequence domains from genes under high-expression (HEP) and low-expression (LEP) regimes served in this work to ascertain quantitatively the relative contribution of efficiency (expression level) versus accuracy during the selection-based codon usage optimization. According to the observed neighbor-joining distances (Table S3 worksheet “distances” and tree in Fig. 4), changes in codon usage derived from differences in gene expression levels—i.e., the efficiency in terms of the distance from the LEP to the HEP—were between 1.25 to 2.35 times greater than the changes in codon usage resulting in increased accuracy—i.e., the distance from *vr* to *cr*. The increasing proportion of highly expressed variable and specially conserved sequences (i.e., HEP_*vr* and HEP_*cr*) in the most ancestral gene sets constituted the basis for explaining the corresponding high degree of codon usage optimization that gradually increased from C1 to Cn.

**FIG 7:**
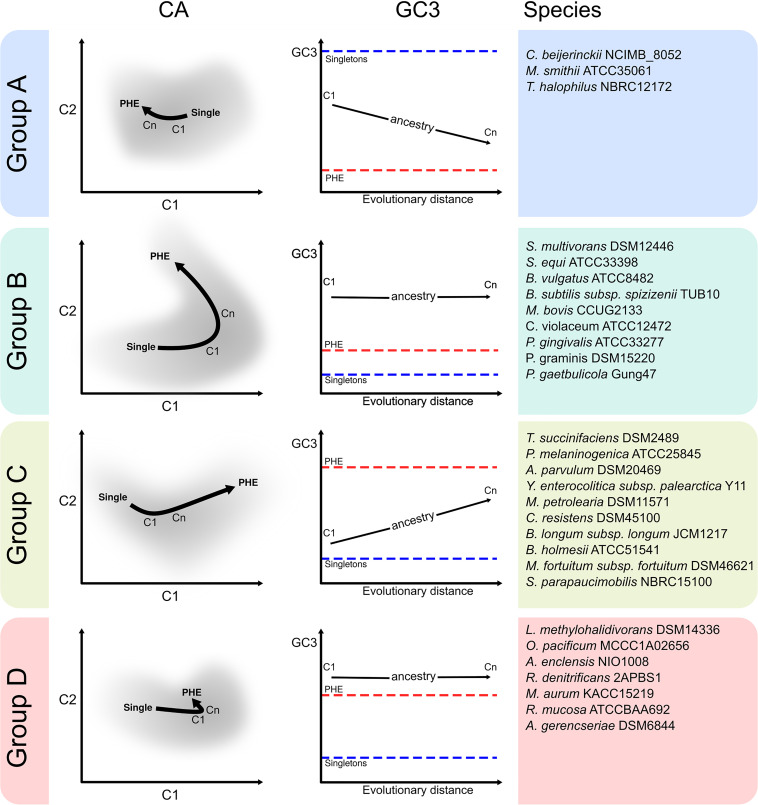
Schematic representation in a cartoon format of general codon-usage patterns observed in different prokaryote families. For the prokaryote strains whose genomes were classified as belonging to groups A, B, C, and D and which are listed to the right of each set of graphs, cartoons with the associated correspondence analysis and GC3 variation pattern among the core gene sets of increasing ancestry (light gray) are presented, along with the corresponding singletons (blue) and PHE genes (red). The light blue arrow indicates the direction of the U bias and the red arrow that of the C bias. The right graph is a plot of the GC3 content on the ordinate as a function of increasing evolutionary distance on the abscissa, with the red horizontal dashed line indicating the PHE genes and the blue the singletons.

The central question therefore was how adaptive changes in codon usage—which alterations become reflected in m-tAI values—occurred in prokaryotes with quite diverse GC contents (10). Because of the small amount of intergenic DNA in prokaryotes, genomic differences in base composition must mainly derive from changes in the coding regions. Within the alterations in the open reading frames, changes in GC are preferentially associated with modifications in the GC3, and only to a lower extent with alterations in the GC content of the first two codon positions (2, 4). How mutational bias (12) competes with selection (15) to drive all these changes is not yet fully understood. The codon usage biases described here were associated with the four different patterns of GC3 changes summarized in the schemes presented in Fig. 7 (i.e., the genome groups A, B, C, and D). The group A genomes, those having an extremely low GC content and with their GC3 frequency decreasing from C1 to Cn, proved to have only the tRNA-U_34_ to recognize 4-fold synonymous codons in one or more amino acids. In such instances, the observed core-gene AT enrichment over ancestry appeared to be directly affected by selection (as with the PHE genes), where codons NNA (via WCIs with the tRNA-U_34_) and NNU (via nonconventional U-U interactions) were preferentially enriched over NNC and NNG codons. Though both of those changes were probably related to improvements in translation efficiency, such increases are not always reflected in the m-tAIs since, as stated earlier, U-U interactions are not considered in the calculation of that index. Unfortunately, when we (data not shown) and others (36) have attempted to improve the tRNA adaptation index by including additional nonstandard base pairings, we obtained no better results. Nonetheless, under the assumption that the PHE genes are the best adapted to the translational machinery, in genomes with extremely low GC content—such as those belonging to group A—the observed AT3 enrichment from C1 to Cn to PHE (Fig. 7, right side) should mainly result from selection. According to Hildebrand et al. (15), the mutational processes in very-low-GC organisms favor a GC3 enrichment. That the core and PHE genes in bacteria that belonged to group A had been selected to bear lower GC3 values than singletons in order to improve translation in view of the previous pattern of increasing GC content was remarkable, with this circumstance being a result of the above-mentioned enrichment in NNA and NNU triplets compared to their proportion in the synonymous codons (Fig. 7, right side). In group B genomes, the biphasic pattern observed from singletons to PHE genes could be explained by an initial increase in GC3-rich codons from singletons to core genes, followed by a later U bias from core genes to PHE genes. That initial GC3 enrichment followed by a U3 increase was sufficient to explain the basis of the previously reported “rabbit head” distribution of codon usages that characterizes most prokaryote genomes (21, 56). What should be also especially noted is that the PHE genes separated from the Cn (in both the CA and the GC3 plots) because of a much more intense U bias likely associated with the difference in expression levels between the two gene sets. In the type C genomes, in which the GC3 always increased, the absence of a strong U bias from the Cn to the PHE genes led to a less pronounced—i.e., more linear—“rabbit-head” distribution of genes in the CA plot. In addition to that general trend, Yersinia enterocolitica, Methanolacinia petrolearia, and Sphingomonas parapaucimobilis could be considered as having behavior intermediate between that of the bacteria in group C and that of the bacteria in group B. Finally, the group D genomes, which had extremely high GC contents, were the most restricted with respect to GC3 variations. The quite small compositional variations in that group of genomes became apparent in the compacted location of the different core and PHE genes in the CA plots. What was remarkable is that in group D genomes a U bias (though much less intense than in the genomes of groups A, B, and C) was still a visible variable that differentiated codon usages between the core and the PHE genes. As stated above, the noninclusion of U-U interactions in the m-tAI calculation limited the use of this parameter to expresses the translational adaptation of those gene sets in which a U bias was dominant. Pouyet et al. (11) present a model to predict and separate the relative contribution of mutational bias (N layer), codon selection (C layer), and amino acid composition (A layer) on the global GC content and the GC3 content. Our analysis is fully consistent with the results reported by Pouyet et al. (11) where the C layer (codon selection/translational selection) has a stronger influence on the GC3 of genes with low effective number of codons (Nc) (such as Cn and PHE) compared to the influence on genes with the highest Nc (such as C1).

The results presented here together with previous evidence from other authors have enabled a comprehensive analysis of the principal basis underlying the changes associated with the optimization of codon usage in prokaryotes in different gene sets and in organisms with different GC contents. As stated previously, the overall codon usage is known to be constrained by genome-wide mutational processes (7, 8, 10) that are considered a main force in shaping the global GC content. The intragenomic codon usage will concurrently become accommodated through selection-driven processes, as has also been extensively reported (32, 34, 41, 47). In order to further our knowledge of the relevance of those factors/forces generating intragenomic variations, we investigated the different nucleotide base changes underlying the selection of preferred codons in the core and PHE genes of representative prokaryote species. The analysis of gene sets with different expression levels and degrees of conservation in organisms with diverse global GC contents enabled a description of how core codon usage approaches that existing in the PHE genes and how nucleotide changes correlate with an improved compatibility between the genes and the coexisting tRNA pool. That C- and U-ending codons in 2-/3-fold and in 4-fold degenerate amino acids, respectively, were specifically enriched as a result of selection to improve translation has been previously reported for different prokaryotic genomes (40). Using separate analyses focused on different gene sets, we demonstrated in this study that similar selection-driven adaptations in codon usage have taken place from singletons to core genes to PHE genes. The intensity and relevance of the C and U biases were dependent on the particular genome—and especially on the genomic GC content—as well as on the gene fractions under consideration. In contrast to the codon usage variations occasioned by selection in the core and PHE genes, the singletons constituted the gene set characterized by both the lowest GC3 content as a result of the AT mutation that is universally biased in prokaryotes (12) and a much more relaxed selection than that of the most ancestral genes, with the sole exception of the extremely low-GC-containing genomes of group A. In addition to a description of the basic changes that together conform the intracellular codon usage variations, further investigation should be focused on the analysis of the time course required by the newly acquired information to be properly incorporated into the genetic language of the host cell (codon usage tuning).

## MATERIALS AND METHODS

### Prokaryote families selected for analysis in this study and identification of core genes and singletons.

We screened the EDGAR public project database (57, 58) available at https://edgar.computational.bio.uni-giessen.de/cgi-bin/edgar_login.cgi, chose several prokaryote families that included at least 20 complete genomes each, and finally selected 27 bacterial and 2 archaeal families (Table S1a, tab 1). A specific core gene set was defined as a group of genes whose orthologs are present in a given set of species under investigation. For each of the families selected, sequential core gene sets with increasing ancestry (C1 through Cn) were calculated. To that end, first the phylogenetic tree for each family was extracted from EDGAR and one species per family was chosen as a reference. Next, the different core gene sets were obtained by incorporating into the analysis new species having sequentially increasing phylogenetic distances from the reference strain (accordingly, by following the tree from the branches to the root). Table S1a to c lists the phylogenetic trees used for these calculations as well as the particular species that were included in each core gene set (C1 to Cn) for the different prokaryote families. The phylogenetic trees were edited with the Figtree (59) and Inkskape programs (TEAM-Inkscape). At least six core gene sets differing in size from ca. 50 to 100 genes each were calculated per family. Table S2, tabs 2 to 30, lists the singletons—those corresponding to genes that were specific to the reference strains with no orthologs within the family—as calculated with EDGAR.

10.1128/mBio.00766-20.7 TABLE S1b Phylogenetic trees of prokaryote families used in this study (continues from Table S1a). Download Table S1b, XLSX file, 2.7 MB. Copyright © 2020 López et al. 2020 López et al. This content is distributed under the terms of the Creative Commons Attribution 4.0 International license.

10.1128/mBio.00766-20.8 TABLE S1c Phylogenetic trees of prokaryote families used in this study (continues from Table S1b). Download Table S1c, XLSX file, 1.5 MB. Copyright © 2020 López et al. 2020 López et al. This content is distributed under the terms of the Creative Commons Attribution 4.0 International license.

10.1128/mBio.00766-20.9 TABLE S2 PHE genes and lists of singletons in all prokaryote organisms used in this study. Download Table S2, XLSX file, 2.7 MB. Copyright © 2020 López et al. 2020 López et al. This content is distributed under the terms of the Creative Commons Attribution 4.0 International license.

10.1128/mBio.00766-20.10 TABLE S3 Estimation of expression data for selected proteins, and modal codon usage distances among natural and simulated sequences for the model organisms presented in Fig. 4 to 6. Tabs 1 to 6, column A, protein expression data obtained from the PaxDB database for orthologs of the translated products of genes that are listed in column B; column B, core gene set under analysis for the indicated model organism used in this study; column C, amino acid sequence conservation (percent) among orthologs from genomes included in the core gene set under analysis. Tab 7, modal codon usage distances among natural and simulated (SIM) sequences presented in the neighbor-joining trees from Fig. 4. Download Table S3, XLSX file, 1.5 MB. Copyright © 2020 López et al. 2020 López et al. This content is distributed under the terms of the Creative Commons Attribution 4.0 International license.

### PHE genes.

For each of the selected reference genomes, we obtained a set of genes encoding ribosomal proteins and tRNA synthetases (24, 60). Table S2, tab 1, itemizes the PHE (putative highly expressed genes) whose orthologs were obtained and analyzed in each reference genome.

### Codon usage diversity groups.

The prokaryote species studied in this work were classified into four different groups based on the compositional characteristics of their codon usage. Eubacterial and archaeal species were classified into groups A to D according to their global GC contents and to the relative GC3 contents (i.e., percent GC at the third position of codons) among their core genes (C1 to Cn), PHE, and singletons; as follows: group A, which included species with very low global GC (<36%) and where GC3^singletons^> GC3^C1–Cn^> GC3^PHE^; group B, which included species with low to intermediate global GC (48% in average) and where GC3^C1-Cn^ >>> GC3^PHE^ and GC3^singletons^; group C, which included species with intermediate global GC (53% on average) where GC3^PHE^ > GC3^C1-Cn^ > GC3^singletons^; and group D, which included species with very high global GC (68% in average) and where GC3^C1-Cn^ was greater than or comparable to GC3^PHE^ > GC3^singletons^. Groups A and group D were those that included the species with lower and higher global GC contents, respectively.

### Highly and lowly expressed proteins within the same core gene set.

Integrated expression data from the Protein Abundance Database (PaxDB [61]) were retrieved for the bacterial strains Yersinia pestis CO92, Streptococcus pyogenes M1 GAS, Campylobacter jejuni subsp. *jejuni* NCTC 11168, Bacillus subtilis subsp. *subtilis* strain 168, Bacteroides thetaiotaomicron VPI 5482, and Mycobacterium tuberculosis H37Rv. Assuming that orthologs have comparable expression levels within the same—or closely related—species and using the PaxDB data from the above-indicated 6 strains, we inferred putative expression data for the proteomes of the microorganisms presented in Fig. 4 to 6 and listed in Table S3. Then, for selected core fractions, we obtained one subset of genes encoding highly expressed proteins (HEP) plus another subset codifying lowly expressed proteins (LEP). For 23 out of the 29 prokaryotic genomes that we analyzed, no proteome data were available, nor were any in phylogenetically related microorganisms.

### Analysis of codon usage in gene sequence regions that encode either conserved or variable amino acid positions in the HEP and LEP subsets.

Individual genes that belonged to the HEP and LEP groups were aligned with the corresponding orthologs. Then codons corresponding to conserved and variable amino acid positions in the HEP genes were separated and each concatenated to generate the HEP_*cr* and HEP_*vr* sequence groups. Through the use of a similar procedure with the LEP genes, the LEP_*cr*, and LEP_*vr* sequences were also generated. Codons categorized as belonging to the *cr* group were those associated with positions with fully conserved amino acids throughout the alignment. Codons categorized as belonging to the *vr* group were those associated with positions where none of the amino acids aligned (at that specific point) reached a proportion higher than 0.5. The modal codon usage (46) of each collection of *cr* and *vr* sequences were calculated and used for further analysis.

### Codon composition based HEP/LEP_*cr*/*vr* distance trees: control trees with artificially evolved sequences under no pressure for codon selection.

Modal codon usage-based neighbor-joining trees were constructed for the indicated gene sets and intragenic regions (*cr* and *vr*) following the method described by Karberg et al. (17) along with the neighbor-joining program of the Phylip package (62). Artificially simulated sequences were used as controls in the neighbor-joining tree. Such generated sequences were evolved under a model with no pressure for codon selection and preserving the same *K_A_*/*K_S_* ratio (i.e., the ratio between non-synonymous to synonymous substitutions) as that corresponding to each of the natural HEP/LEP set of homologs. Amino acid and codon alignments were generated with TranslatorX using MUSCLE (http://translatorx.co.uk/). For proteins in both HEP and LEP groups, we inferred the most likely evolutionary model using the amino acid alignments and modeltest-ng (63) as well as a maximum likelihood phylogenetic tree using codonphyml (64). Next, we used both the inferred trees and codon alignments to optimize a codon evolutionary model using codeml from the PAML suite (65). For simplicity, an M0 model with F3×4 codon equilibrium frequencies was used. F3×4 frequencies avoided the introduction of compositional biases. The obtained parameters of the model, which included the *K_A_*/*K_S_* value for each set of HEP and LEP orthologs, were used to generate simulated DNA sequences for each protein using PAML evolver software. Such artificially generated sequences were used to recover the *cr*/*vr* simulated data sets (namely, the SIM data sets) using CUBACR and the procedure described above to obtain the natural HEP_*cr*/*vr* and LEP_*cr*/*vr* data sets (see previous section). The scripts used for this analysis are included in CUBACR (https://github.com/maurijlozano/CUBACR).

### Correspondence analyses.

**(i) RCC-based analyses.** Raw-codon-count (RCC)-based correspondence analyses (CA) were performed using bash and R-software homemade scripts which can be found at the CUBES software page (this work; available at https://github.com/maurijlozano/CUBES). Briefly, G. Olsen codon usage software was used to count codons on coding sequences (available at http://www.life.illinois.edu/gary/programs.html), data were loaded on R, and the correspondence analyses were run using the FactoMiner (66) and Factoextra (https://CRAN.R-project.org/package=factoextra) packages. Plots were made using the ggplot2, ggrepel, ggthemes, and gtools R packages. For each core gene set, the CA coordinates were calculated as the arithmetic mean of the first and second dimensions of all the genes present in that set (centroids). Then, a plot was generated containing all the coding sequences, together with the projections of the core-gene sets (C1 to Cn), the singletons and PHE genes.

**(ii) RSCU-based CAs and calculation of modal codon usages.** The CAs based on the use of relative synonymous codon usages (RSCUs) (67) of all individual genes from a given genome were calculated by CodonW (68). The modal codon usages (46) were calculated for the core genes, singletons, and PHE genes. Artificial sequences representing modal codon usages (i.e., modal sequences) and the amino acid composition present in each core fraction (Cn) were generated through the use of a homemade Perl script (calculate_modals2.pl) from the CUBES package and incorporated into the CA. In order to accurately represent the modal codon usage, particularly for synonymous codons from low-abundance amino acids, modal sequences were designed with a length of at least 10,000 codons. RSCU-based CA plots (see the supplemental material) were generated through the use of the Ggplot2 program (69) and edited with Inkskape (TEAM-Inkscape).

### tRNA gene copy number and m-tAI.

The gene copy number of each tRNA in the different prokaryote species studied was downloaded from the GtRNAdb server (http://gtrnadb.ucsc.edu), which uses predictions made by the program tRNAscan-SE (70). For each reference genome, the copy number for the tRNAs and the sequences of all the open reading frames were used to train the *S_ij_* weights as previously reported, with that parameter estimating the efficiency of the interaction between the *i*th codon and the *j*th anticodon in a given organism (35, 36). The procedure is, briefly, as follows. With randomly generated *S_ij_* starting points—i.e., having values that range between 0 and 1—the tAI was calculated for each coding sequence by means of the tAI package (35; https://github.com/mariodosreis/tai). Next, the directional codon bias score (DCBS [36]) was calculated through the use of the script seq2DCBS.pl (CUBES package). Finally, the Nelder-Mead optimization algorithm from R project was used (instead of the hill-climbing algorithm) to search for the *S_ij_* value that maximized the Spearman rank correlation between the DCBS and the tAI. A script for bulk *S_ij_* estimation is available in the CUBES package (https://github.com/maurijlozano/CUBES, calculate_sopt_DCBS_GNM_f.sh). The estimated sets of *S_ij_* weights were used to calculate both the *Wi*s―i.e., the absolute adaptiveness values―as originally reported by dos Reis et al. (35) and the modal tRNA-adaptation index (m-tAI) for different species and gene sets (i.e., core and PHE genes plus singletons) as a measure of their efficiency in being recognized by the intracellular tRNA pool. The m-tAIs were calculated from the previously generated modal sequences by means of the tAI_Modal_g.sh script from the CUBES package. The Spearman coefficient was used to characterize how m-tAIs changed, from C1 to Cn, over ancestry.
