# Supplementary material for: Codon Usage Optimization in the Prokaryotic Tree of Life: How Synonymous Codons Are Differentially Selected in Sequence Domains with Different Expression Levels and Degrees of Conservation
Source: mBio. 2020 Jul 21;11(4):e00766-20. doi: 10.1128/mBio.00766-20 (PMC7374057; doi:10.1128/mBio.00766-20)
Supplement: FIG S1 [file mBio.00766-20-sf001.pdf]

FIGURE S1A

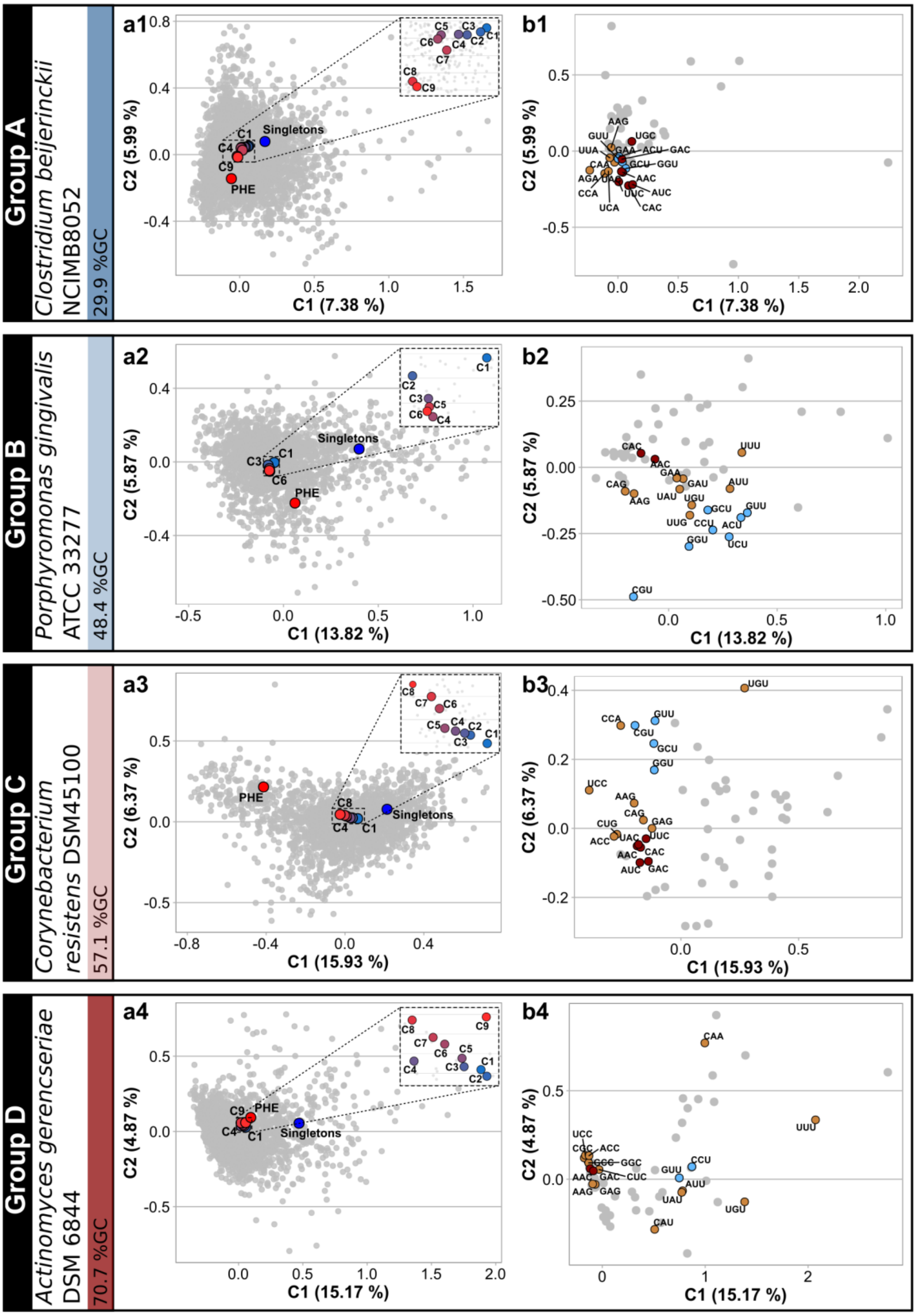

Fig. S1A. RSCU-based Correspondence Analysis (CA) plots of core-gene sets with different degrees of conservation throughout the phylogeny of selected

prokaryote families (Groups A to D). Panels a1 to a4: In 4 reference strains with different GC contents, individual genes (in gray) are represented in the space of the first-two CA components, with the percent variation of components 1 and 2 being indicated on the axes. CAs were computed using RSCUs as the input variables. Modal codon usages—calculated as indicated in Materials and Methods—for different gene sets (i.e. singletons in blue, C1 to Cn in a gradient from blue to red, and PHE in red) were projected on the CA space. In C1 to Cn the higher number denote a more ancestral core-gene set within the phylogeny. **Panel b1 to b4.** Plots describing codon relative weight in the first two principal-component positions of the CA. Codons that are colored in brown indicate those which present—for each amino acid—the highest CUF enrichment from C1 to PHE (i.e. those codons that better represent translational adaptation) except when some of those codons also correspond to a C-bias (colored in red) or a U-bias (colored in light blue).

Fig. S1-B

GROUP A

Methanobrevibacter smithii ATCC 35061

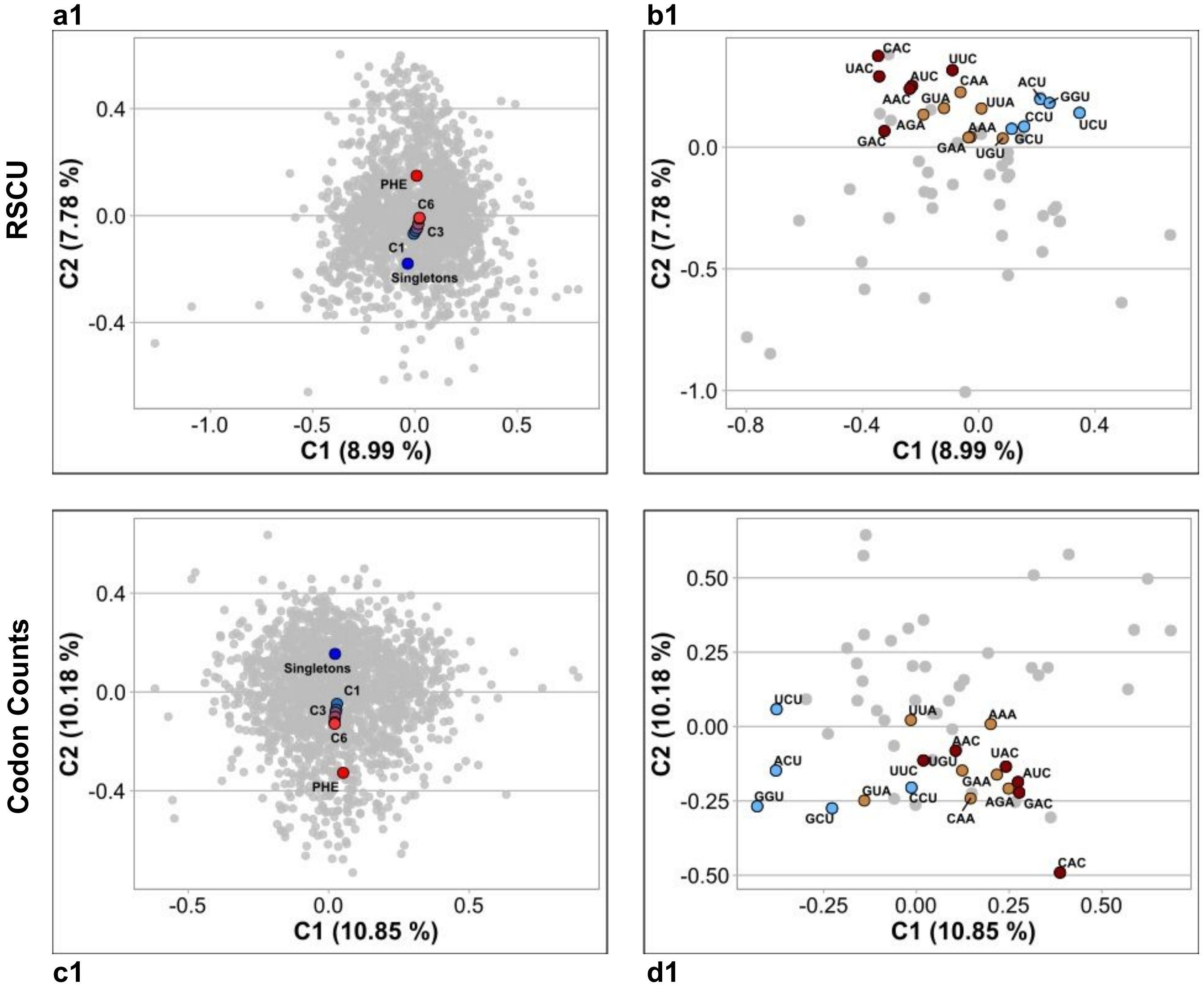

Tetragenococcus halophilus NBRC 12172 NC_016052

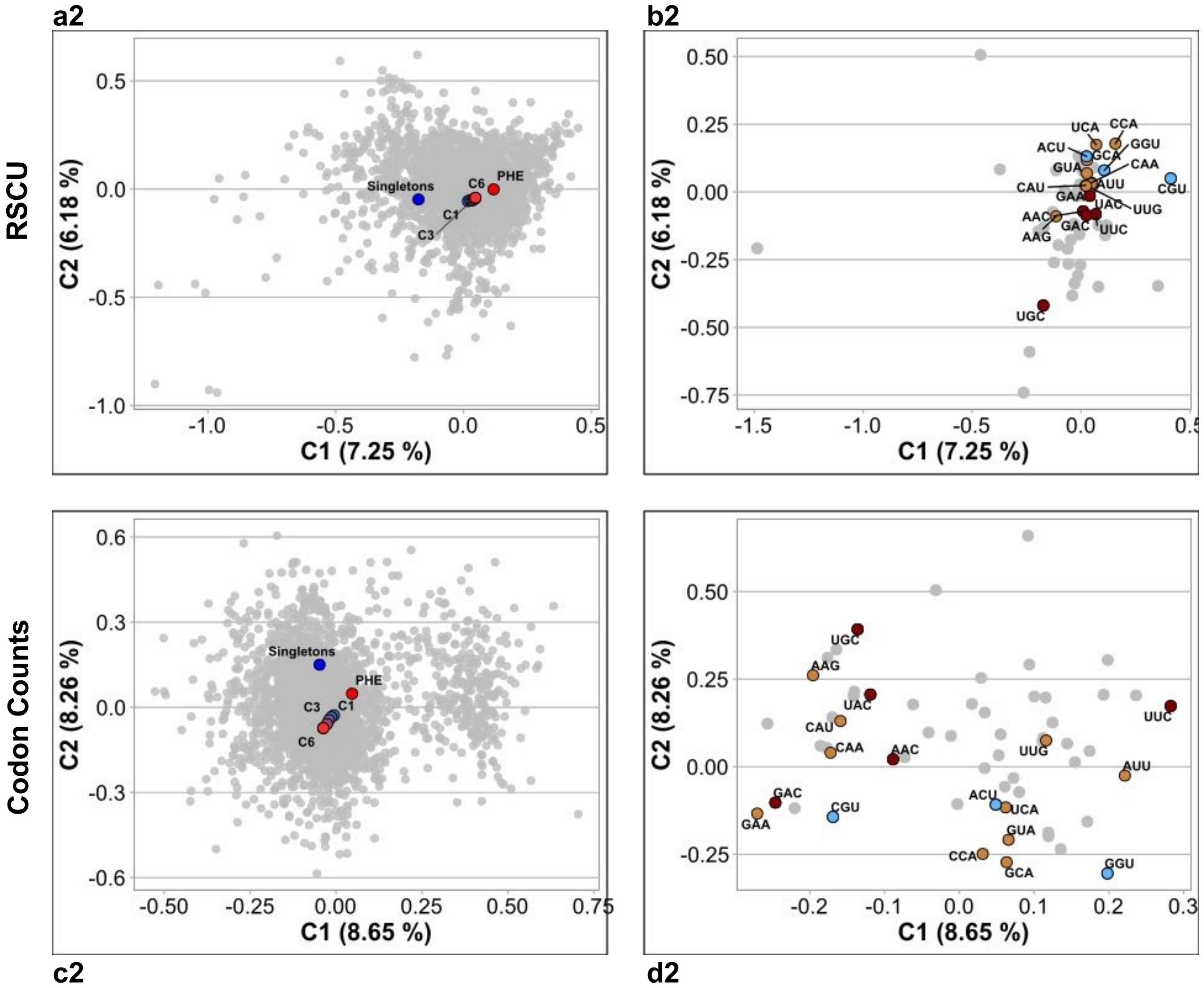

GROUP B

Sulfurospirillum multivorans DSM 12446 NZ_CP007201

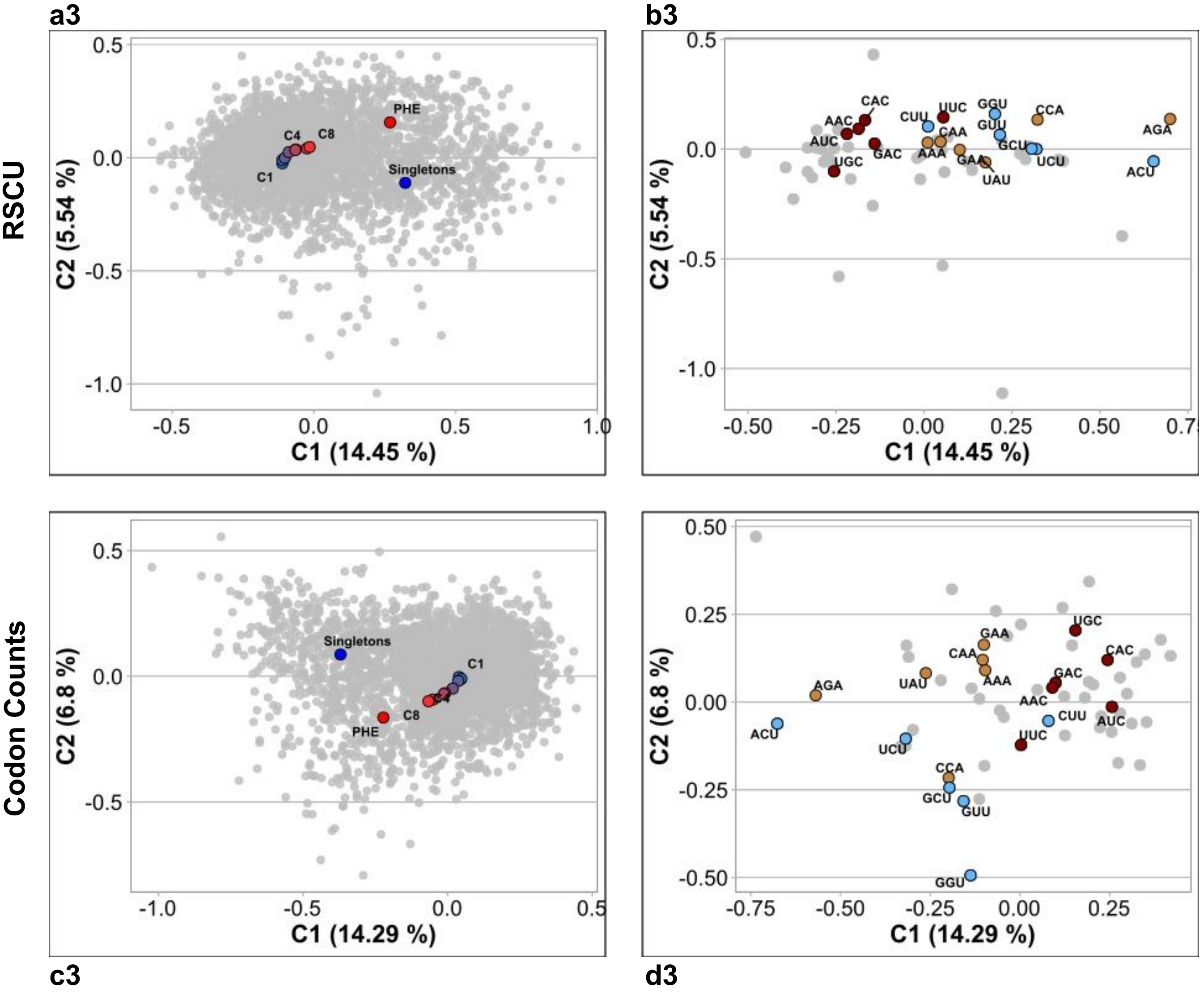

Streptococcus equi ATCC 33398 NZ_FTNH01000046

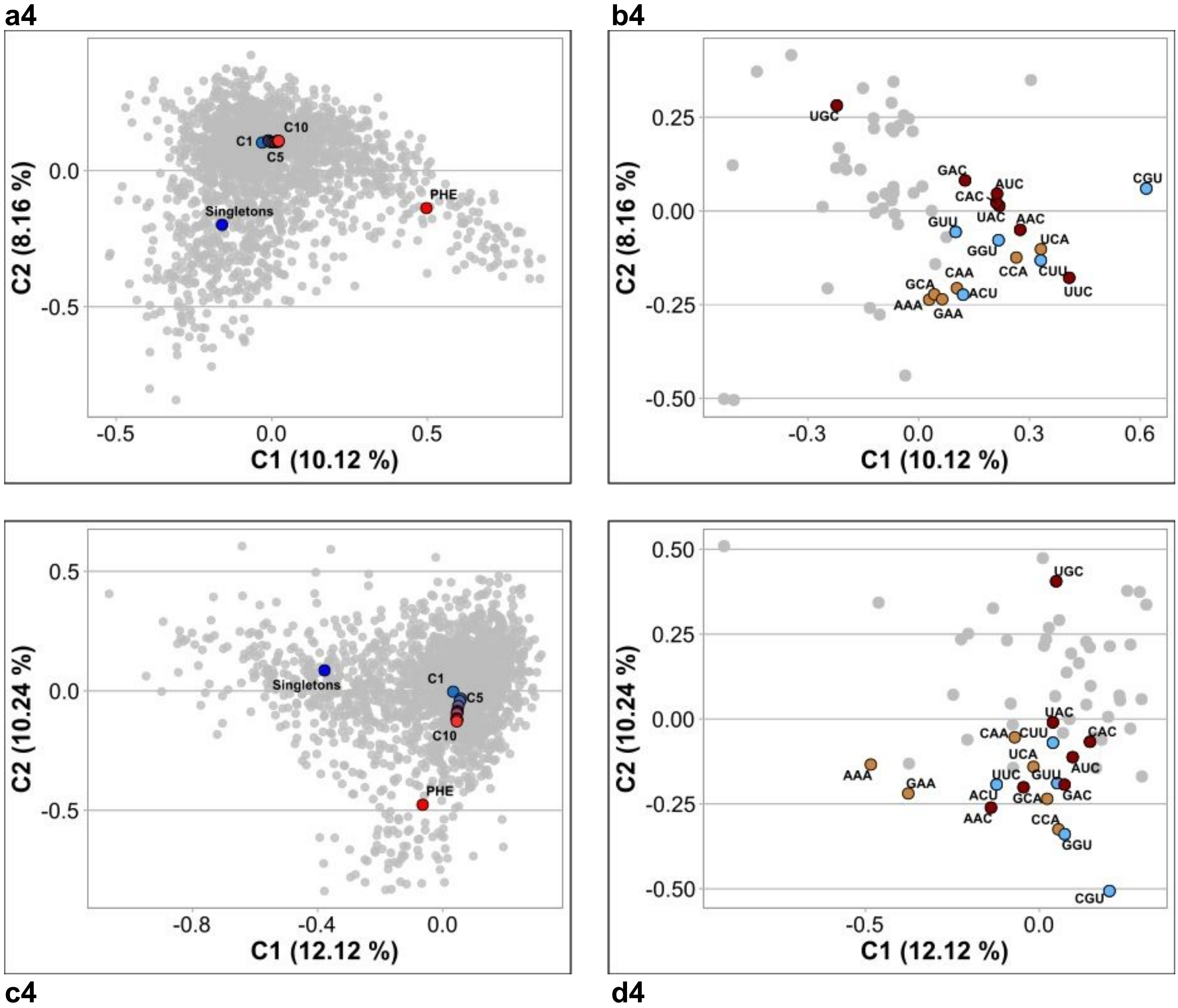

Bacteroides vulgatus ATCC 8482 NC_009614

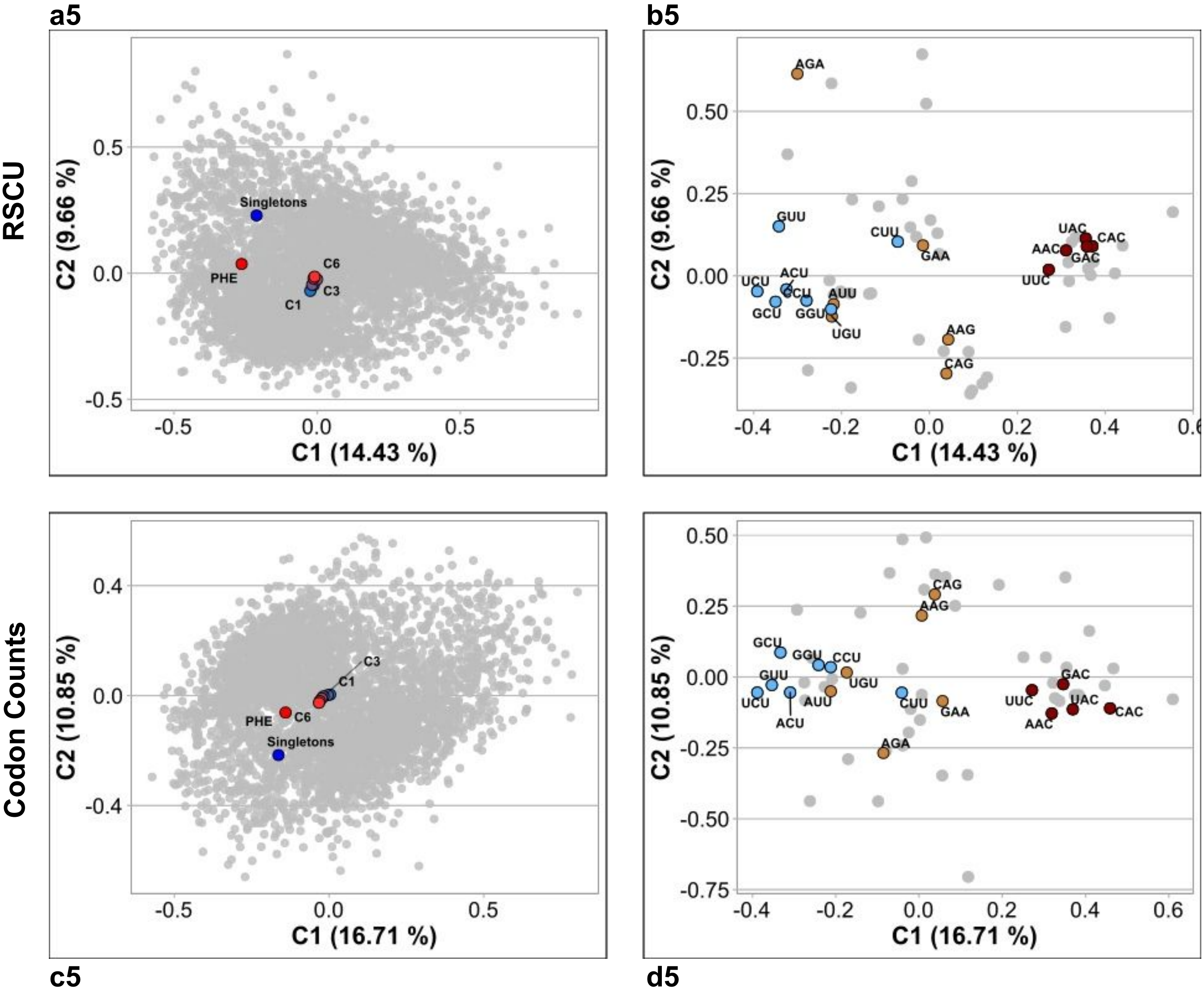

Bacillus subtilis subsp spizizenii TU B 10 NC_016047

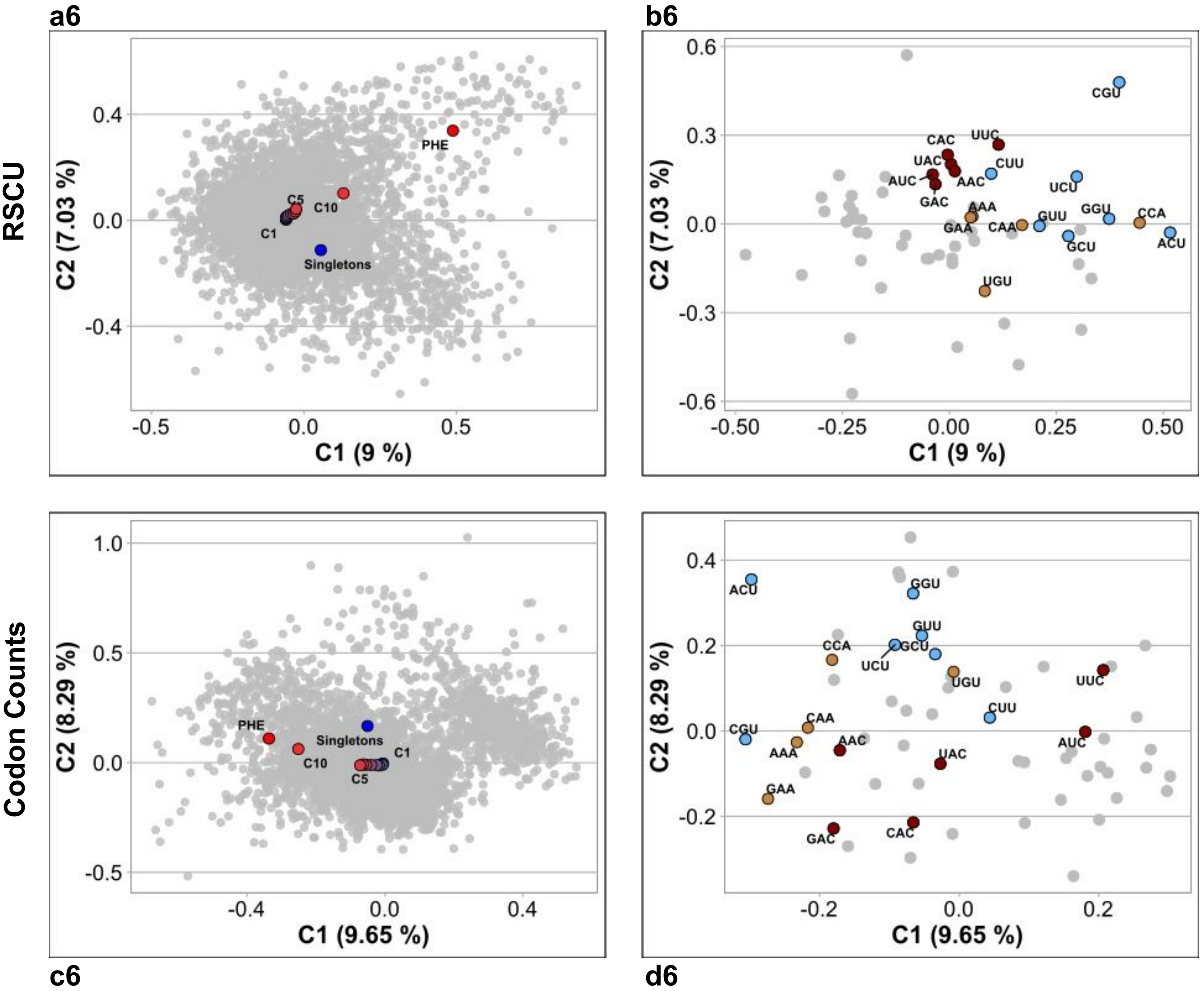

Moraxella bovis CCUG 2133 NZ_MUXV01000108

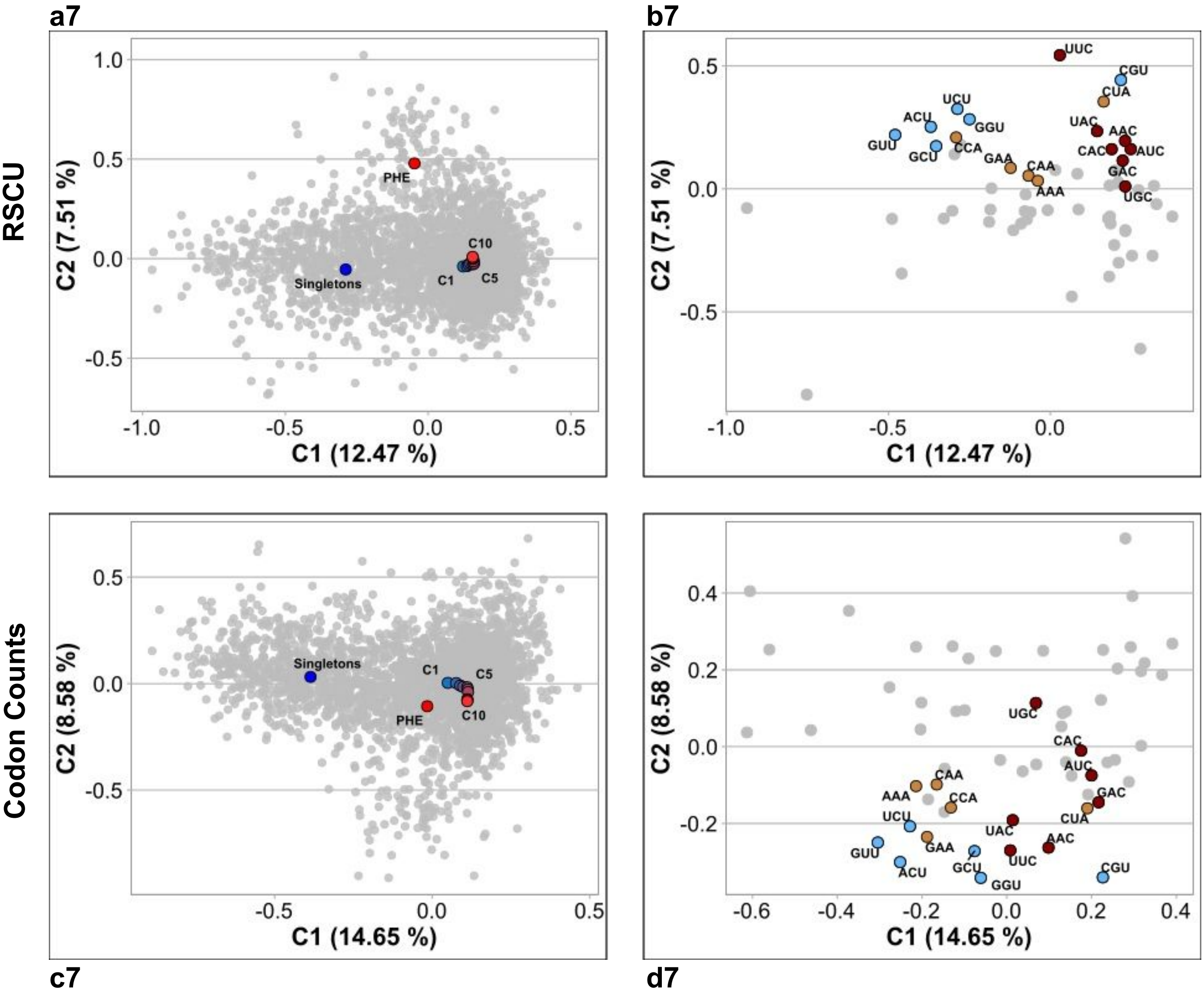

Chromobacterium violaceum ATCC12472

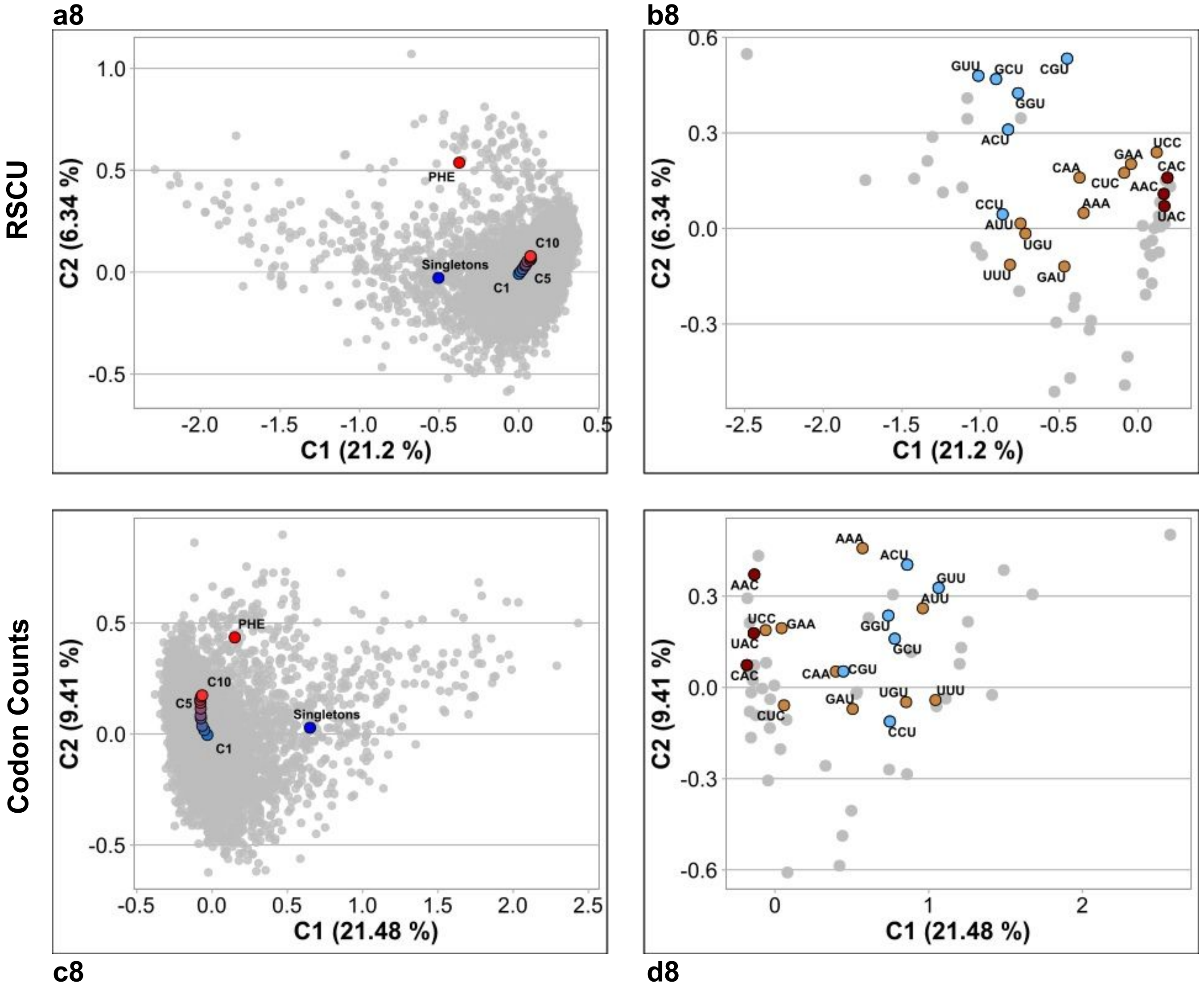

Paenibacillus graminis DSM 15220 NZ_CP009287

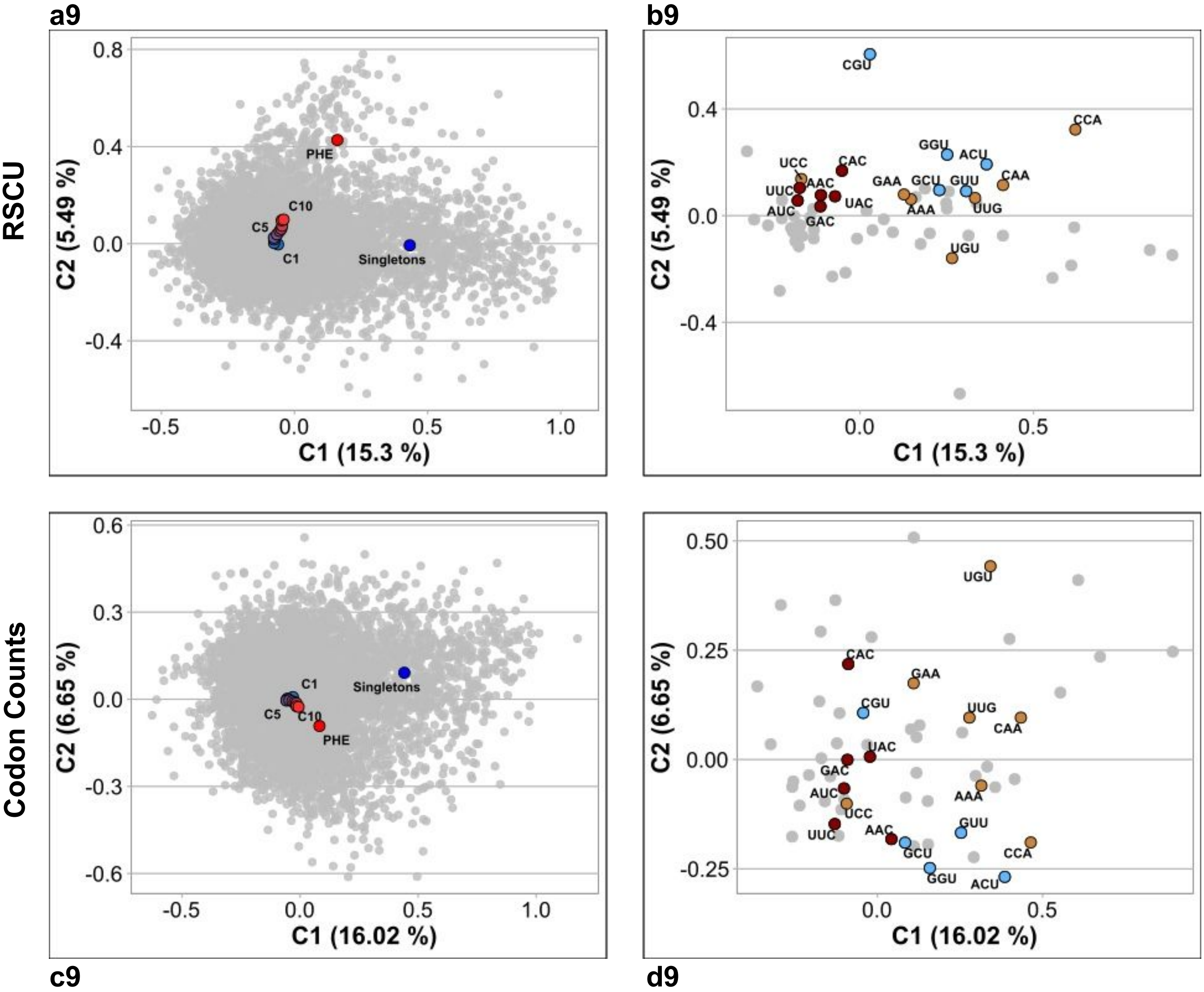

Photobacterium gaetbulicola Gung47

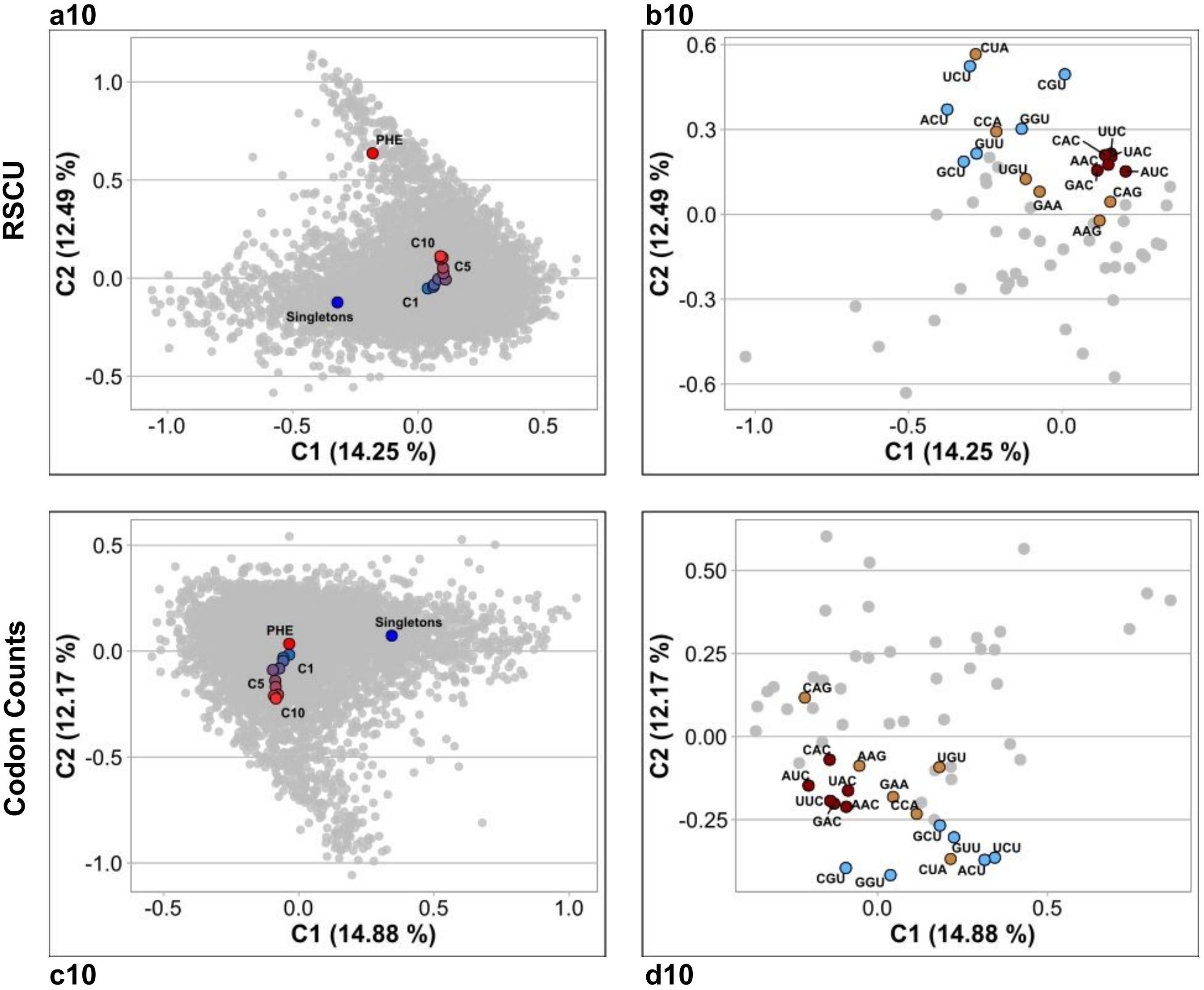

GROUP C

Treponema succinifaciens DSM 2489 NC_015385

a11 b11

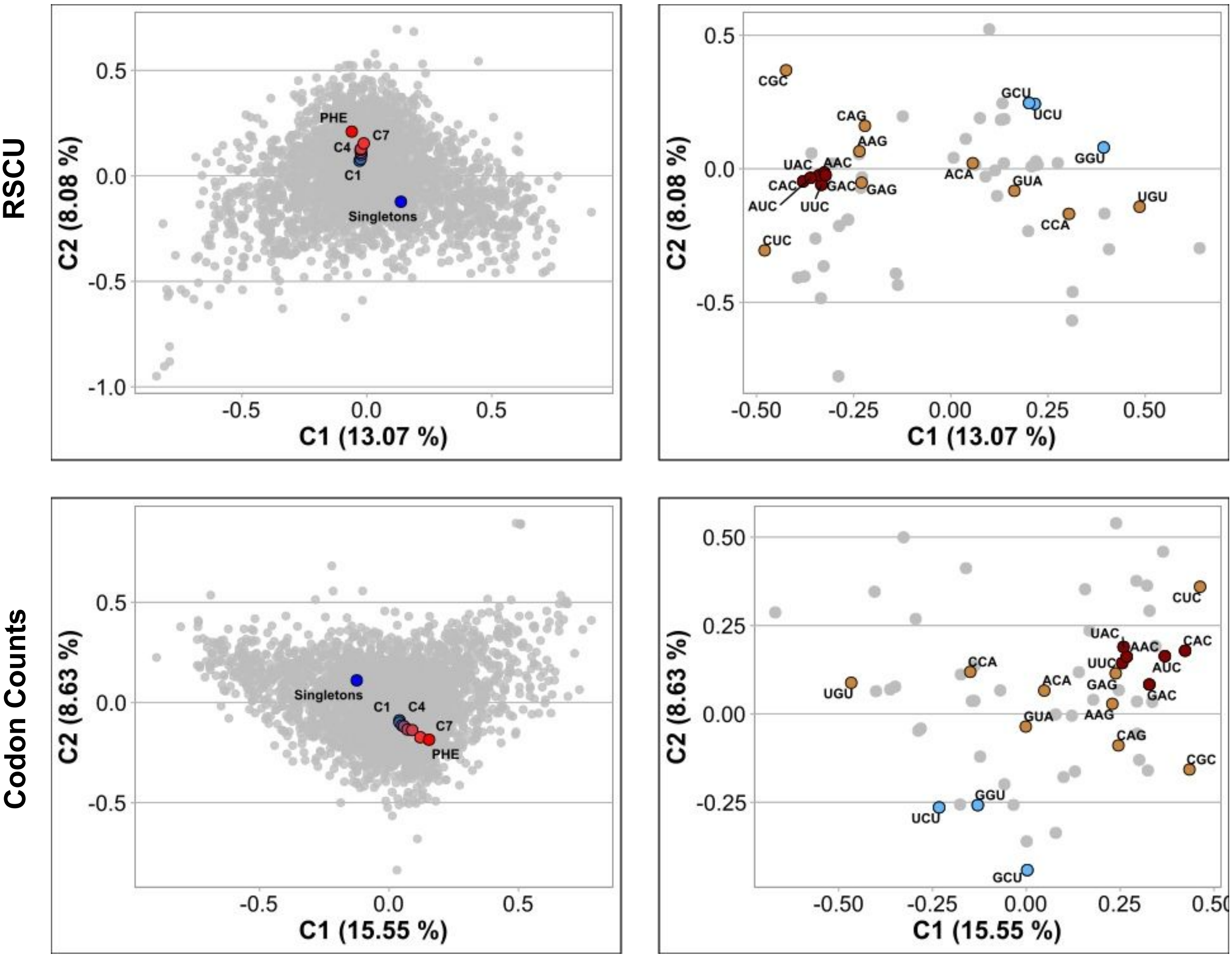

c11 d11

Prevotella melaninogenica ATCC 25845 NC_014371

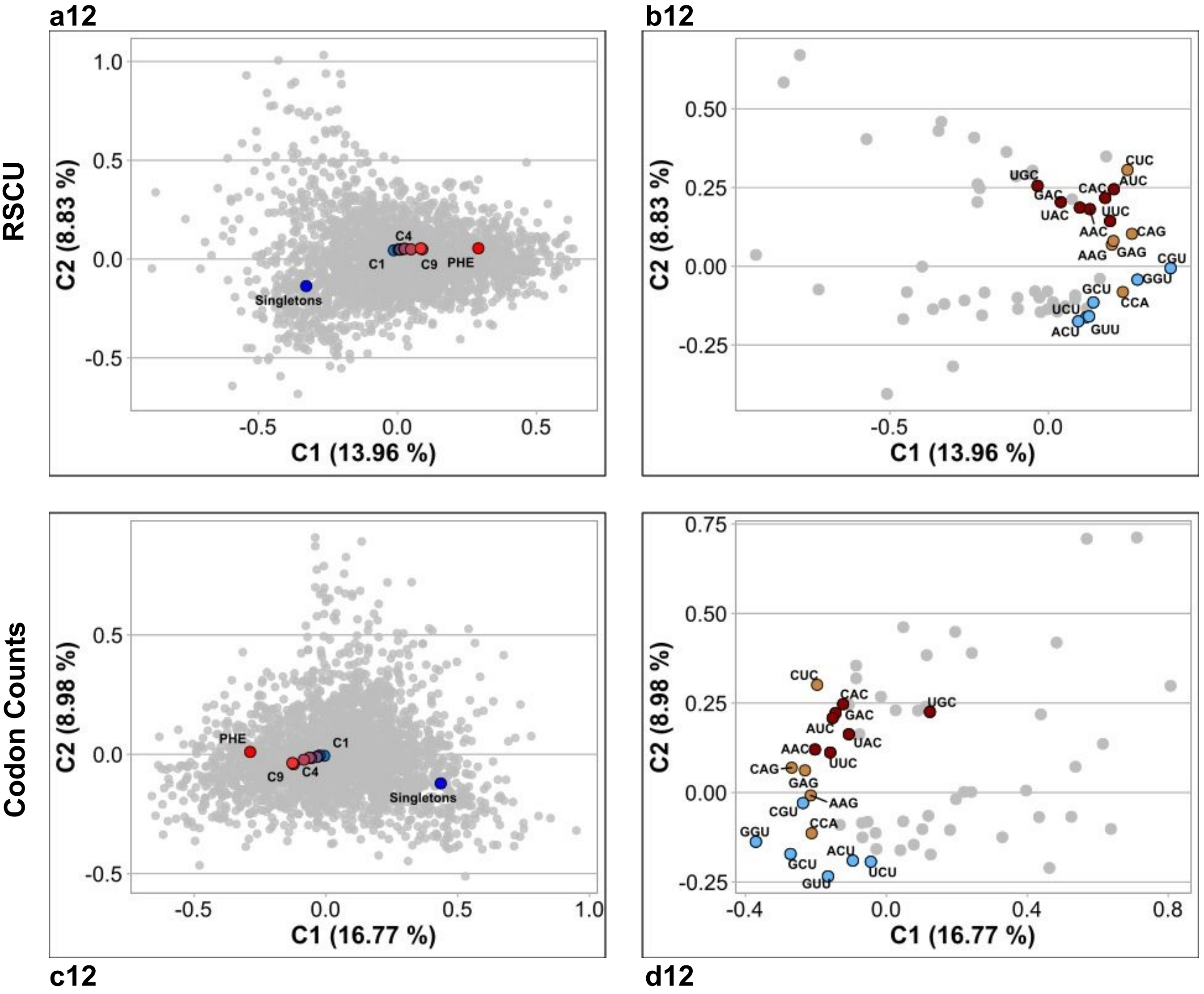

Yersinia enterocolitica subsp palearctica Y11 NC_017564

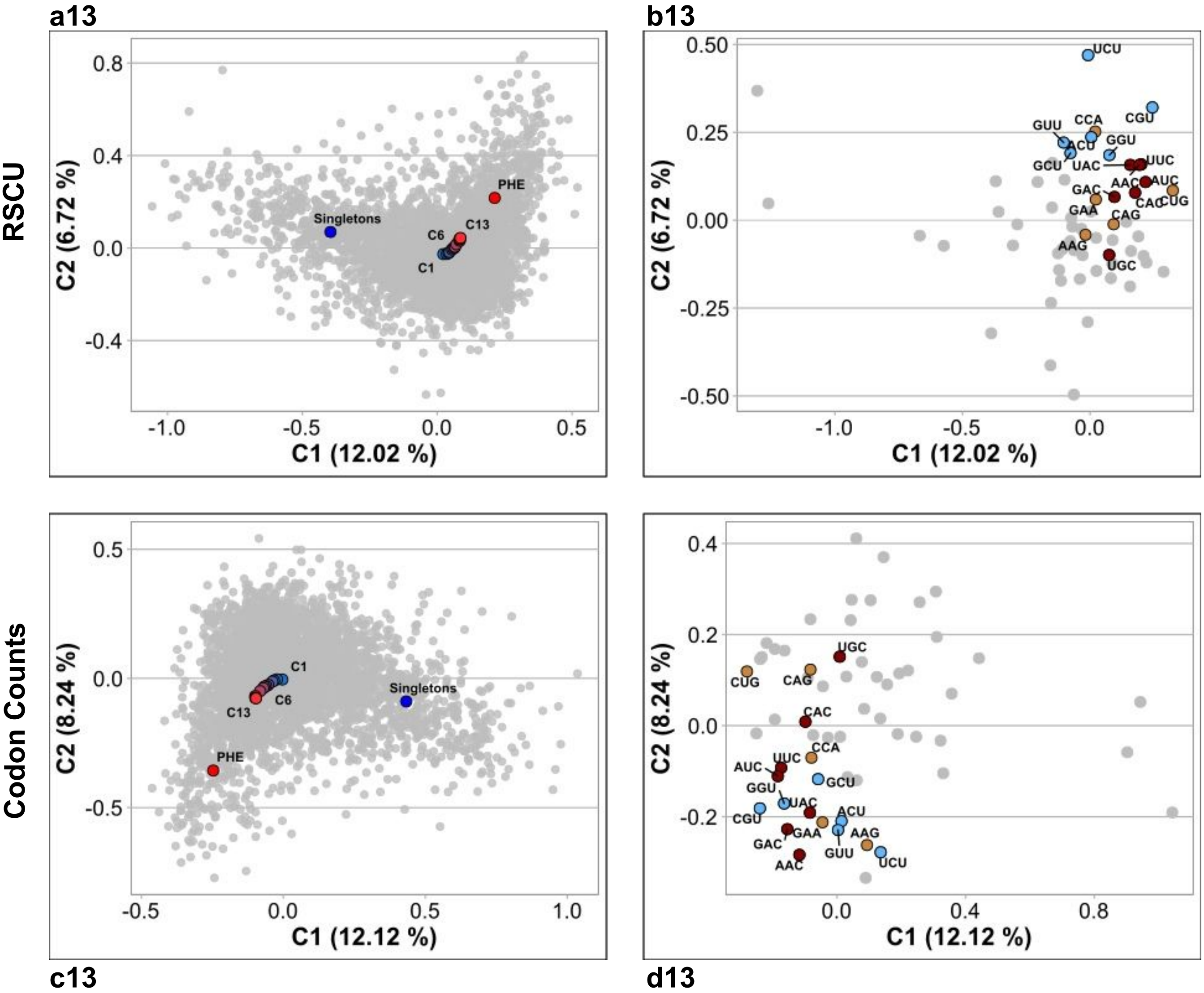

Methanolacinia petrolearia DSM 11571 NC_014507

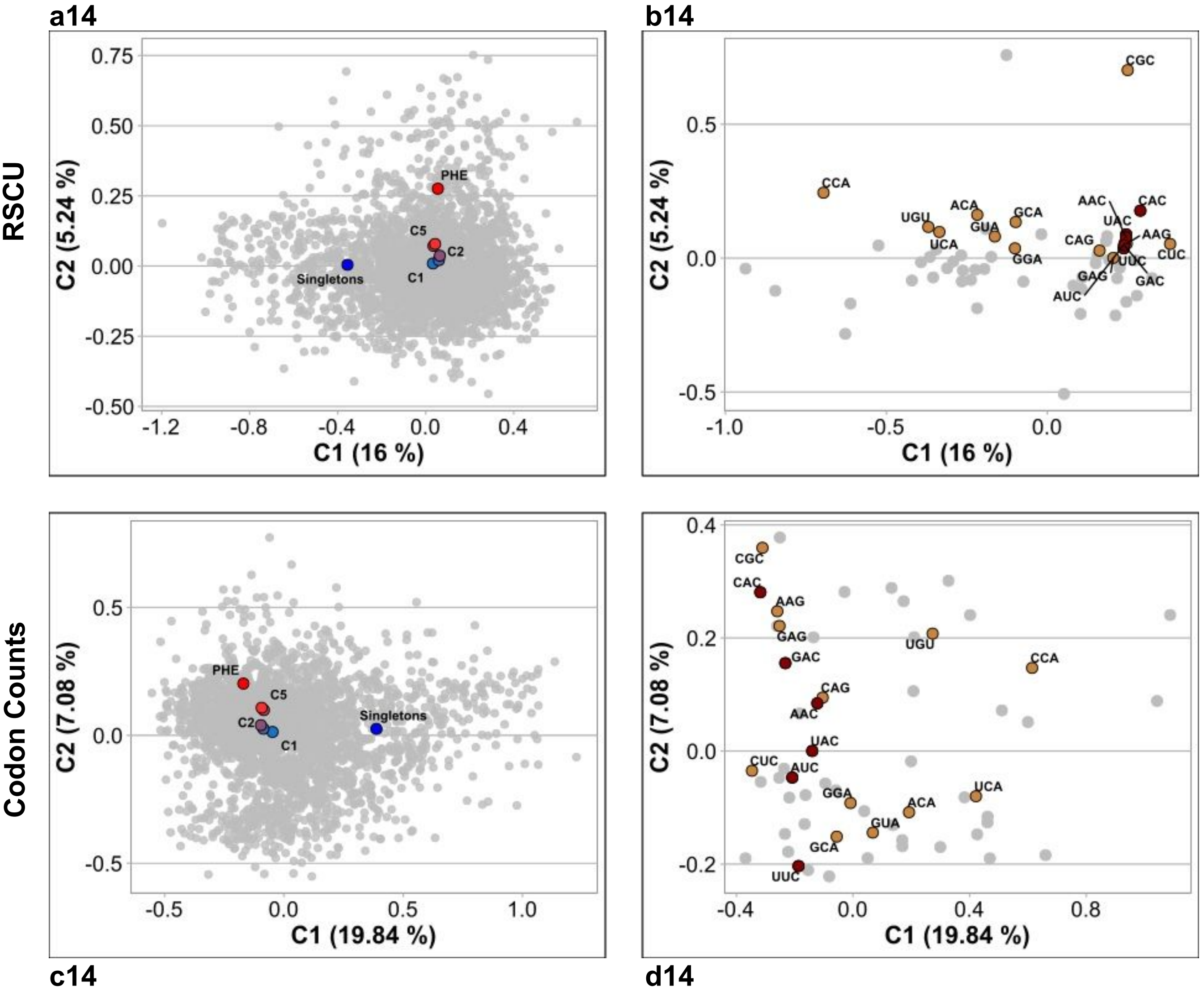

Bifidobacterium longum subsp longum JCM 1217 NC_015067

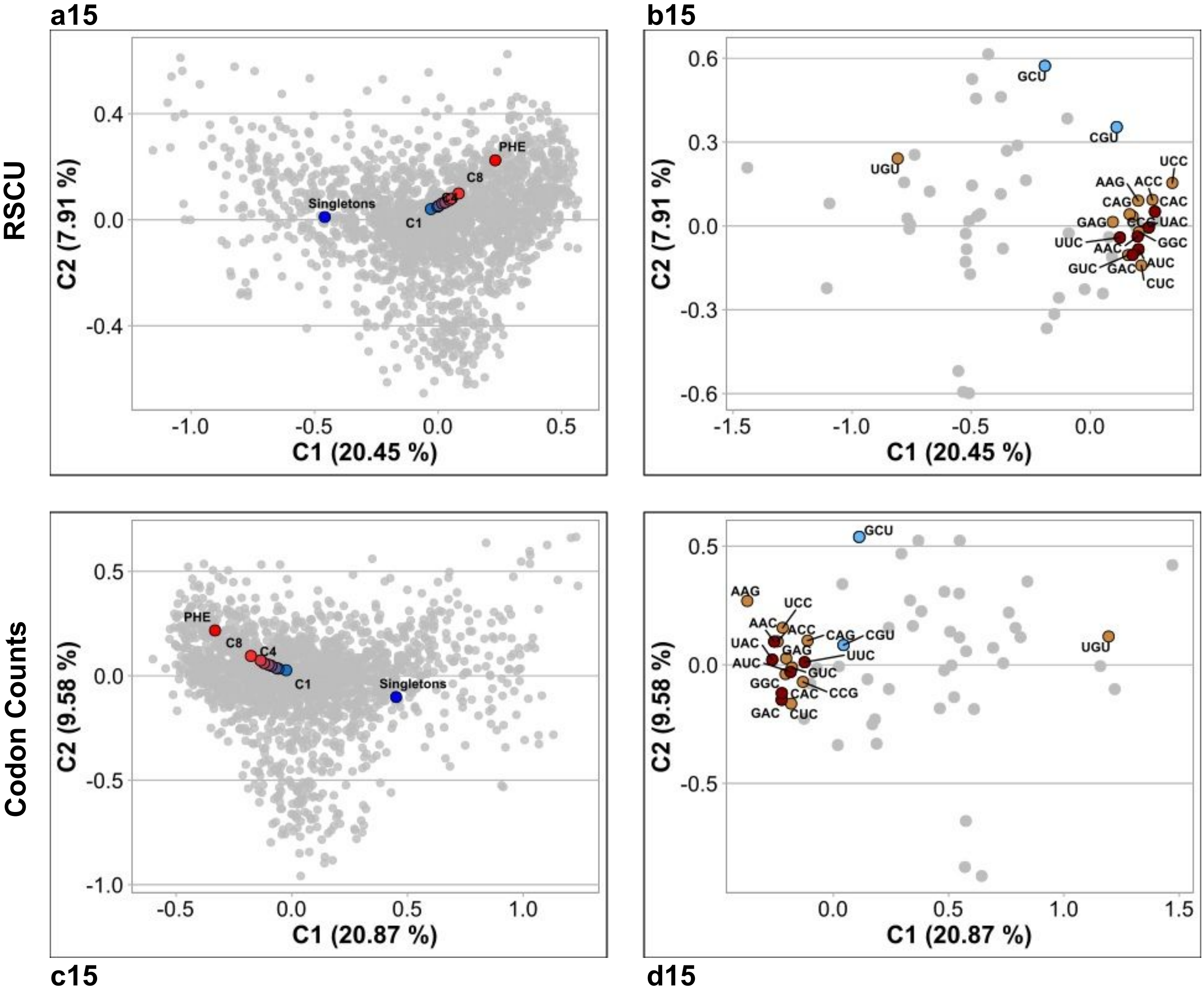

Bordetella holmesii ATCC 51541 NZ_CP007494

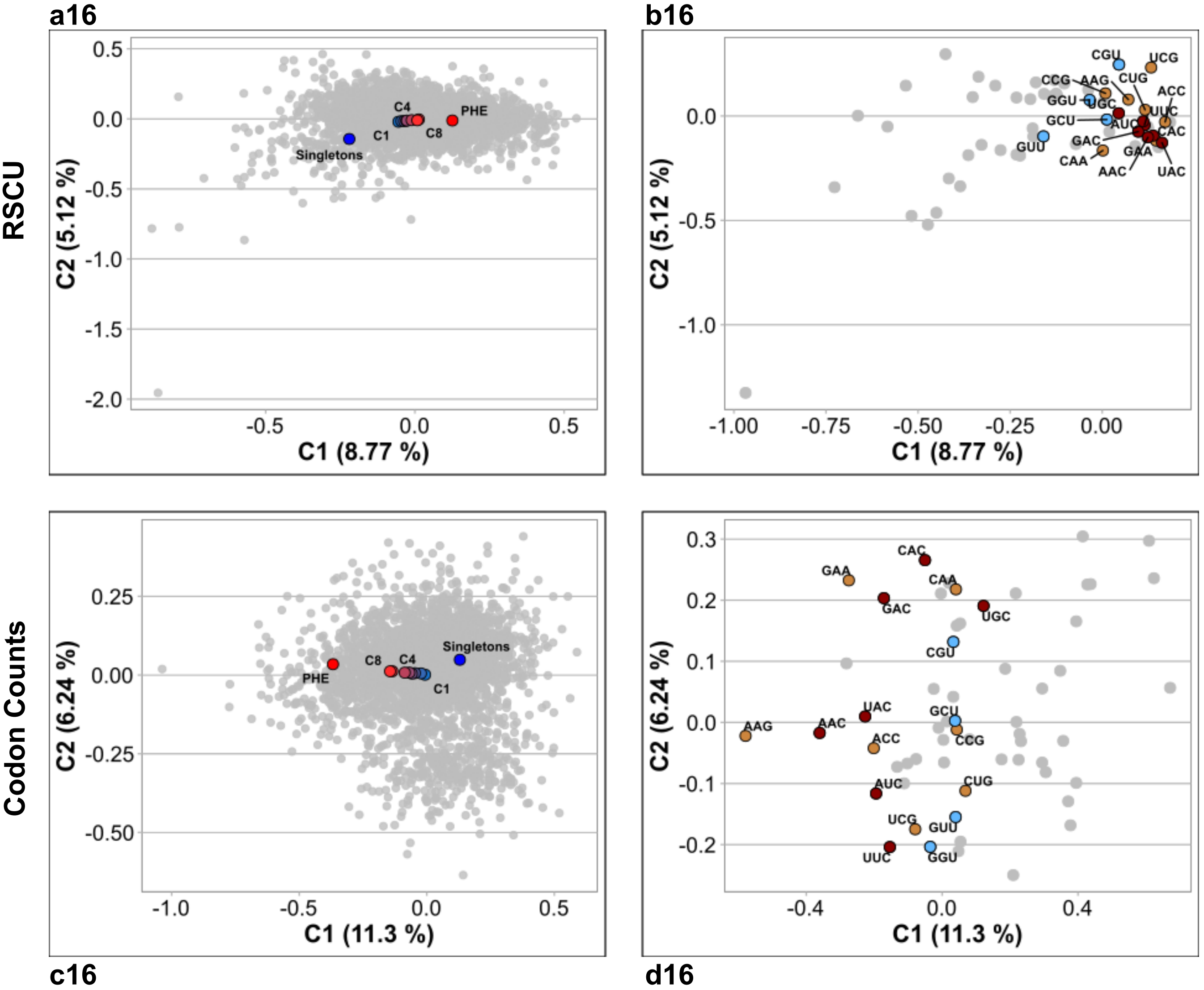

Mycobacterium fortuitum subsp fortuitum DSM 46621 ATCC 6841 CP014258

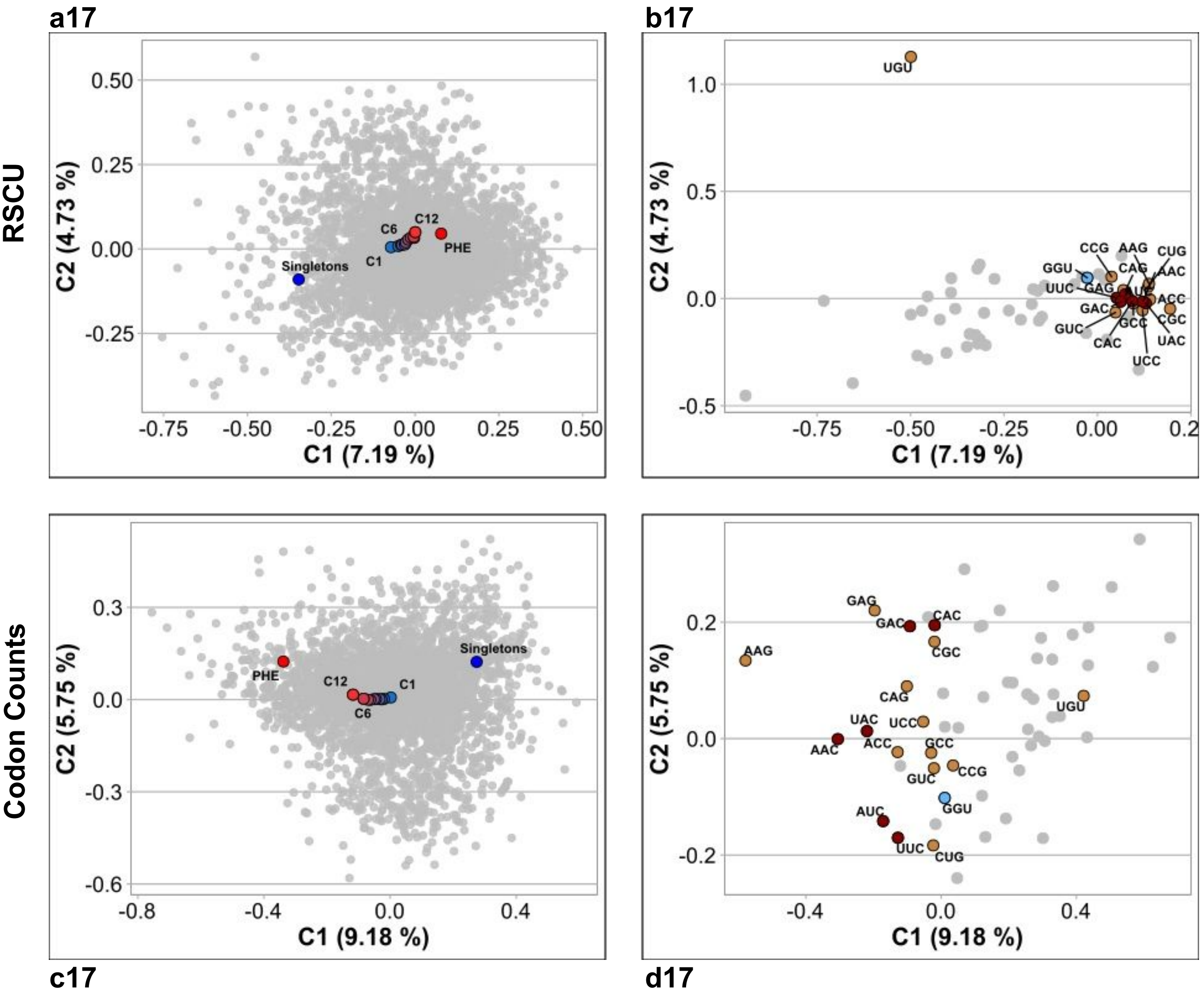

Sphingomonas parapaucimobilis NBRC 15100 NZ_BBPI01000001

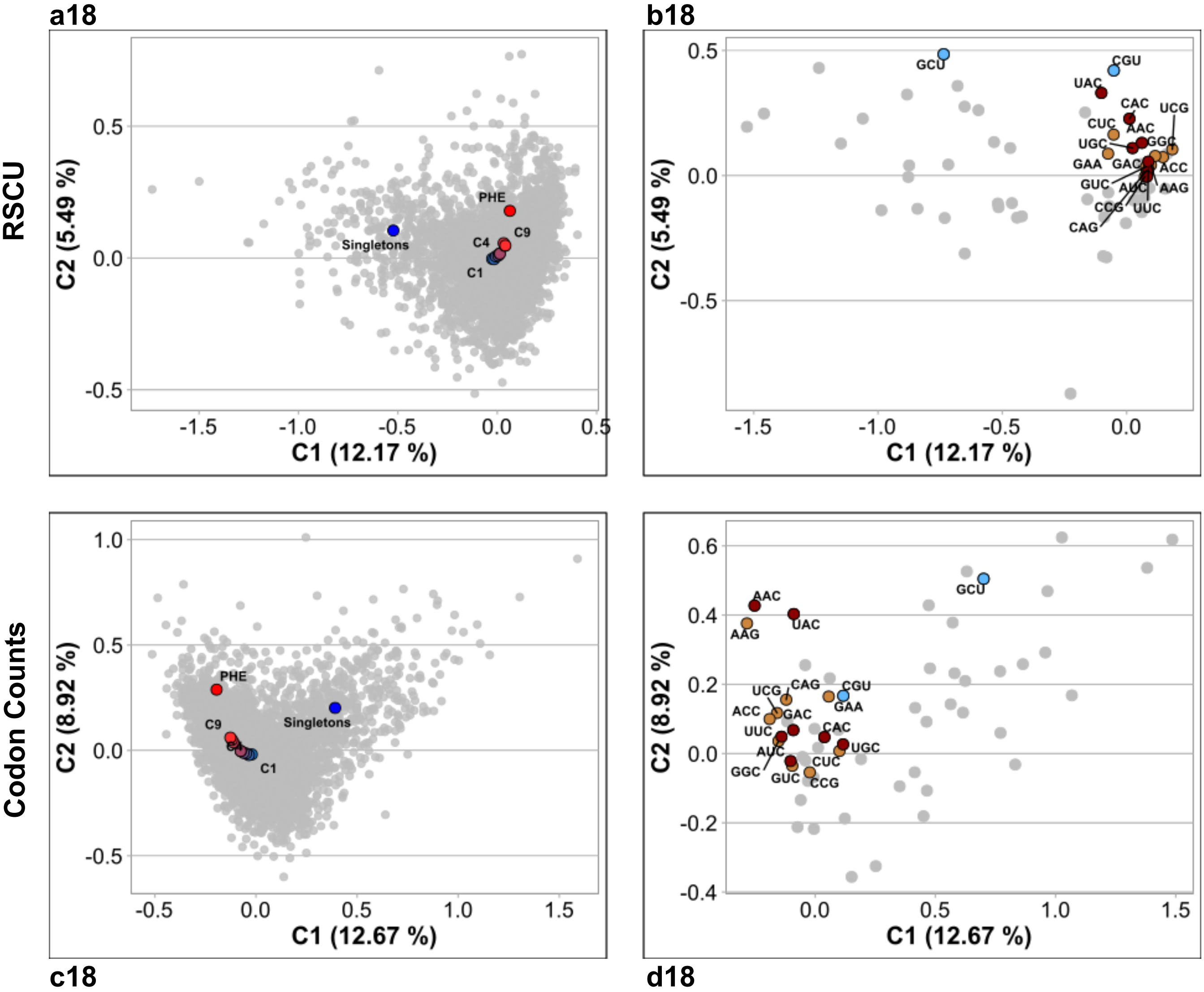

Atopobium parvulum DSM20469

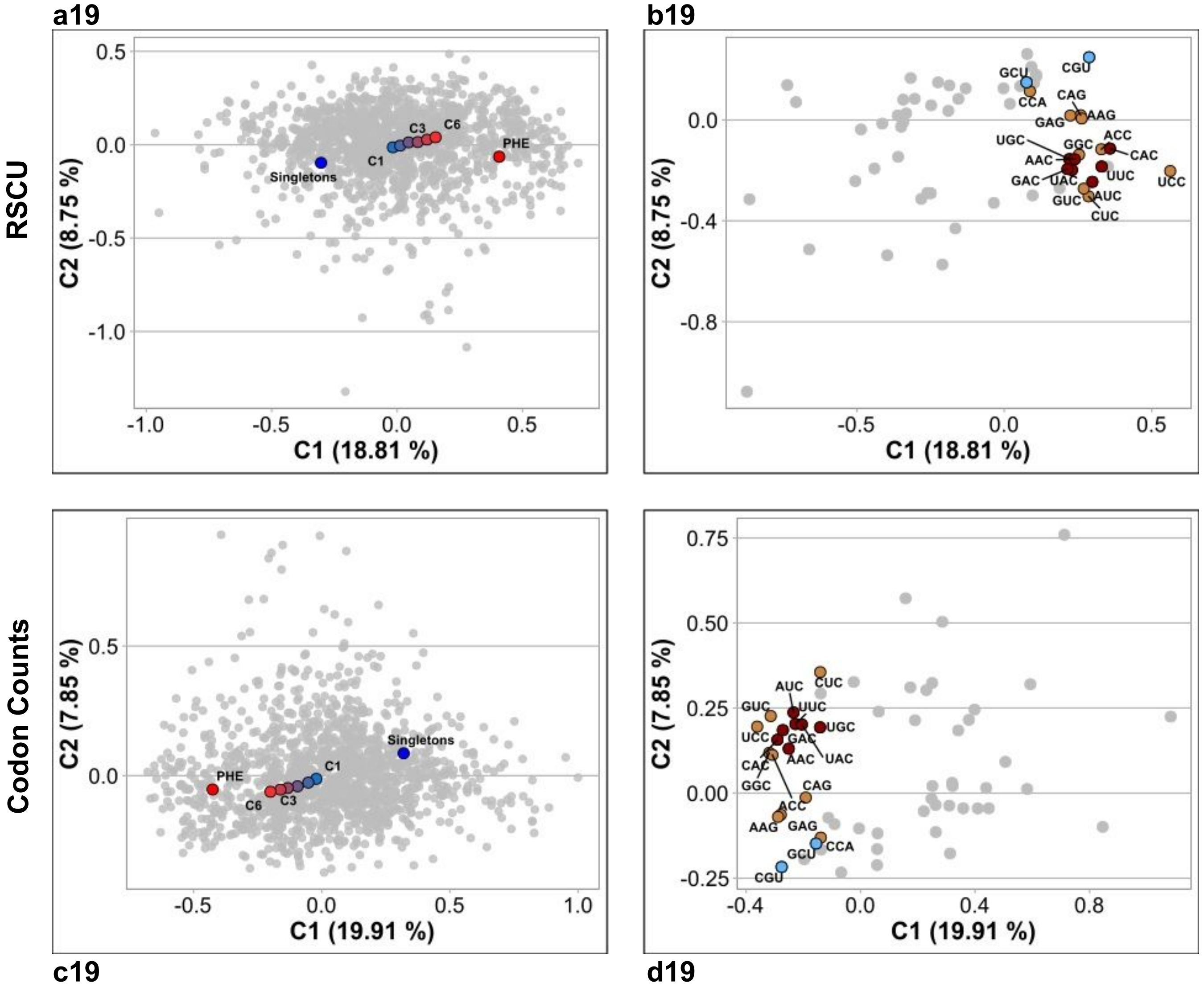

GROUP D

Leisingera methylohalidivorans DSM 14336

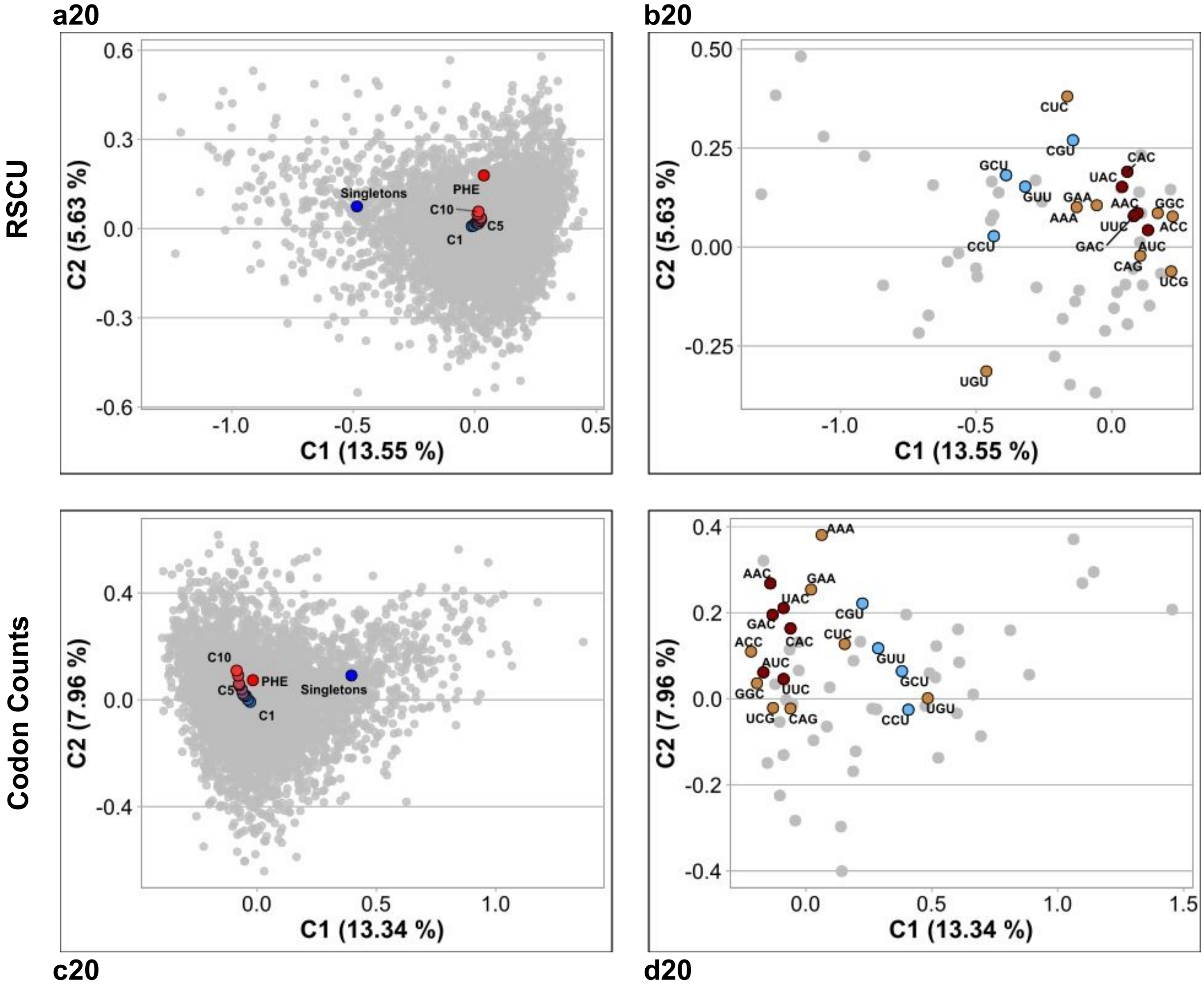

Oceanibaculum pacificum MCCC 1A02656 NZ_LPXN01000181

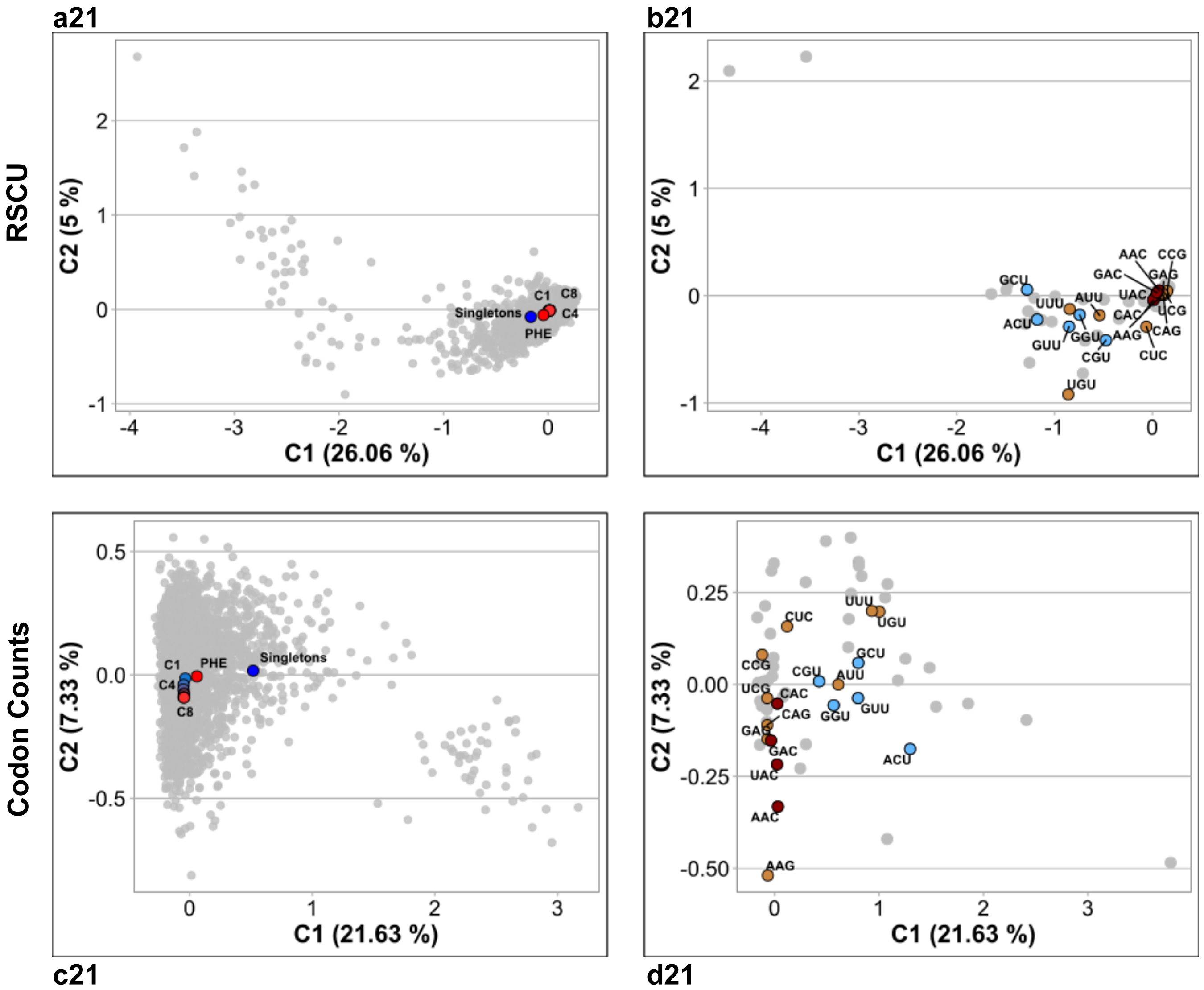

Arthrobacter enclensis NIO 1008 NZ_KQ758616

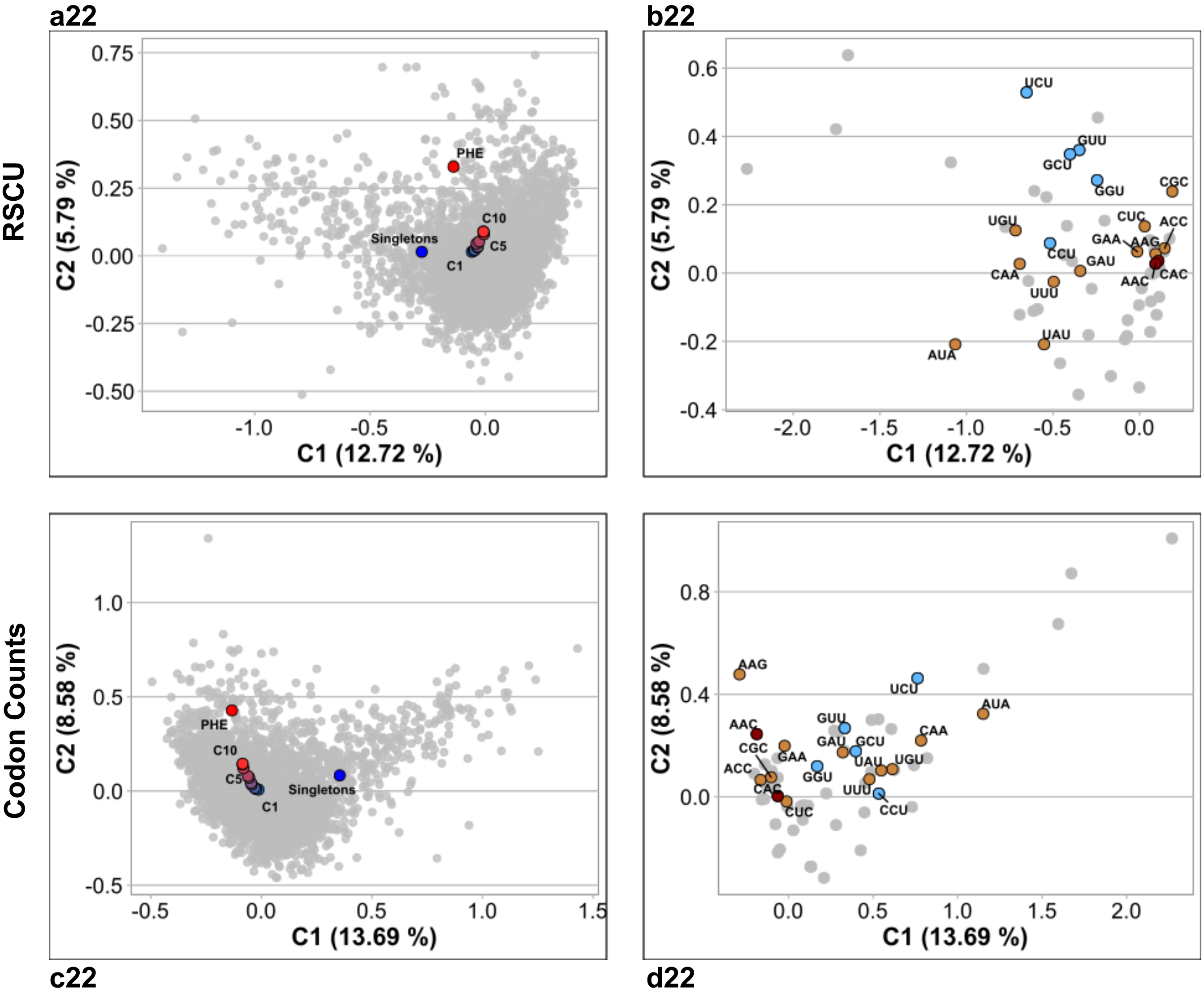

Rhodanobacter denitrificans 2APBS1

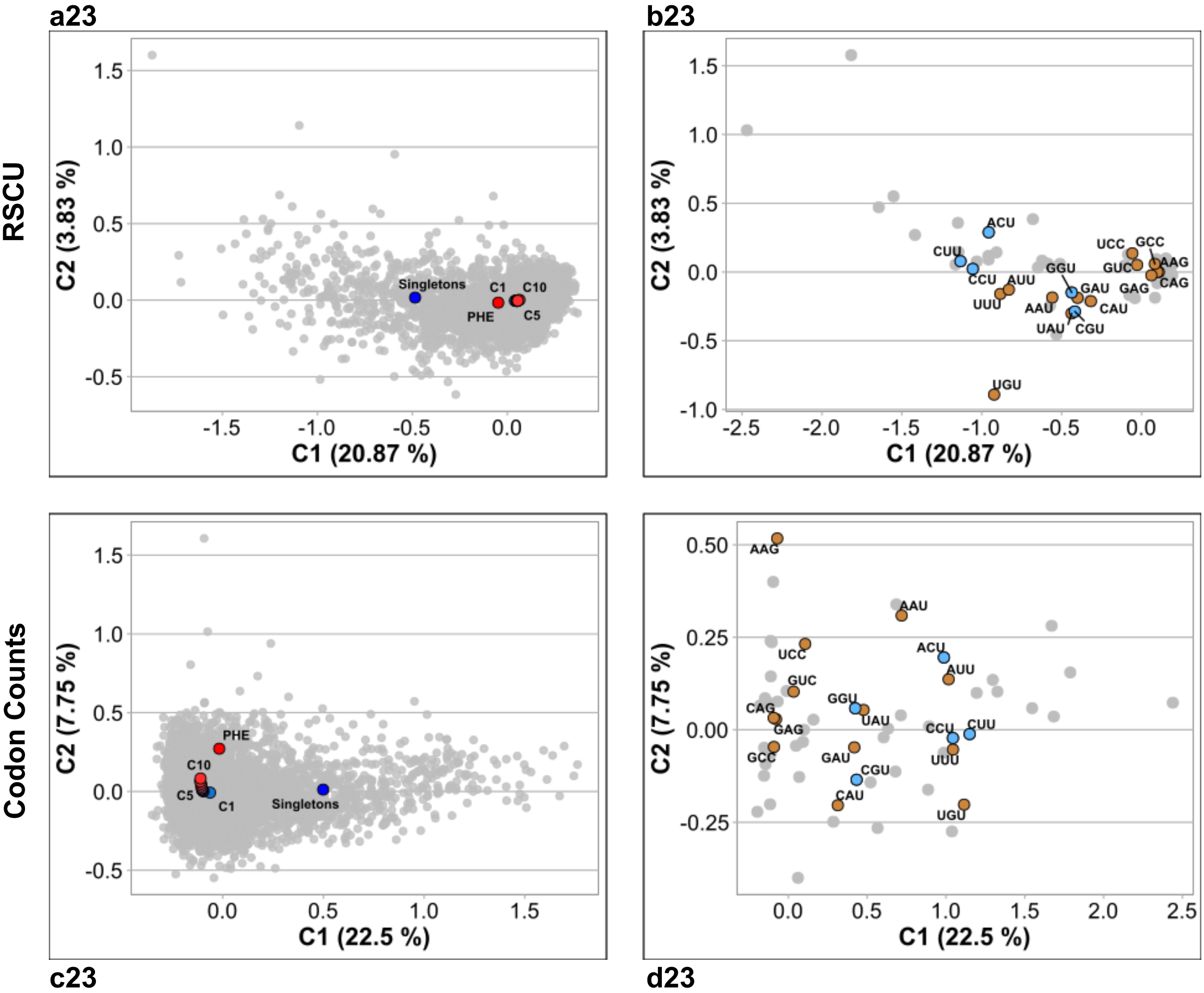

Microbacterium aurum KACC 15219 NZ_CP018762

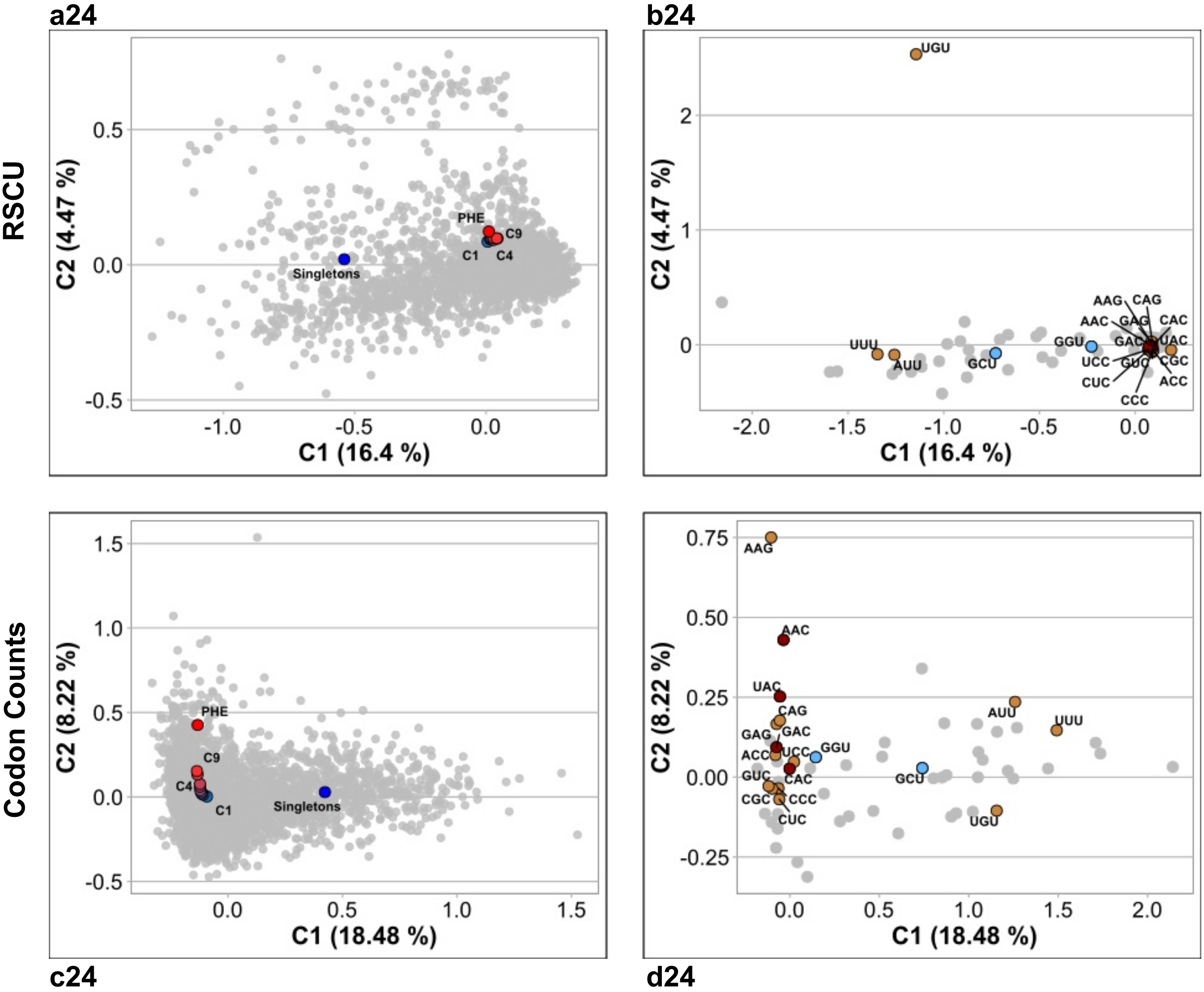

Roseomonas mucosa ATCC BAA 692 NZ_JHWD01000081

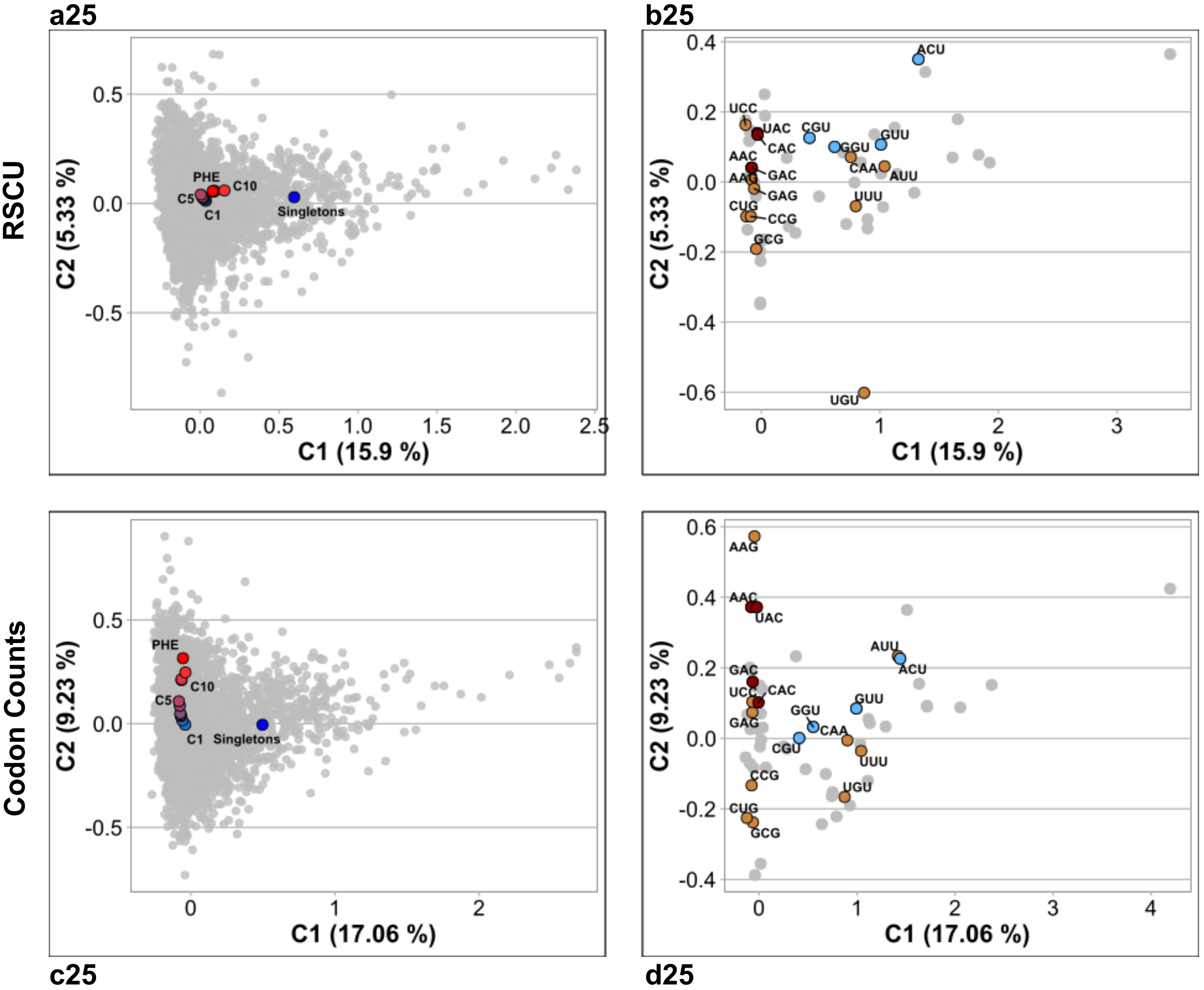

Fig. S1B. Correspondence-Analysis (CA) plots of core-gene sets with different degrees of conservation throughout the phylogeny of 25 prokaryote families. The strains that were not included in the main manuscript are represented here and classified as belonging to Group A, B, C, or D. **Panel a1 to a25:** The individual genes (in gray) are represented in the space of the first-two CA components, with the percent variation of components 1 and 2 being indicated on the axes. CAs were computed using either raw codon counts (RCC) or RSCU as the input variables as indicated. Average coordinates (centroids) for different gene sets (i.e. singletons in blue, C1 to Cn in a gradient from blue to red, and PHE in red) were projected on the CA space in the RCC-based CA plots. Similarly, modal codon usages for the same gene sets—calculated as indicated in Materials and Methods—were projected on the RSCU-based CA plots. In C1 to Cn the higher number denote a more ancestral core-gene set within the phylogeny. Table S1_a (tab 1) lists the prokaryote species that were used to construct each Ci gene set by means of the EDGAR software (54, 55). **Panel b1 to b25.** Plots describing codon relative weight in the first two principal-component positions of the CA. Codons that are colored in brown indicate those which present—for each amino acid—the highest CUF enrichment from C1 to PHE (i.e. those codons that better represent translational adaptation) except when some of those codons also correspond to a C-bias (colored in red) or a U-bias (colored in light blue).
