# Supplementary material for: Codon Usage Optimization in the Prokaryotic Tree of Life: How Synonymous Codons Are Differentially Selected in Sequence Domains with Different Expression Levels and Degrees of Conservation
Source: mBio. 2020 Jul 21;11(4):e00766-20. doi: 10.1128/mBio.00766-20 (PMC7374057; doi:10.1128/mBio.00766-20)
Supplement: FIG S2 [file mBio.00766-20-sf002.pdf]

FIGURE S2

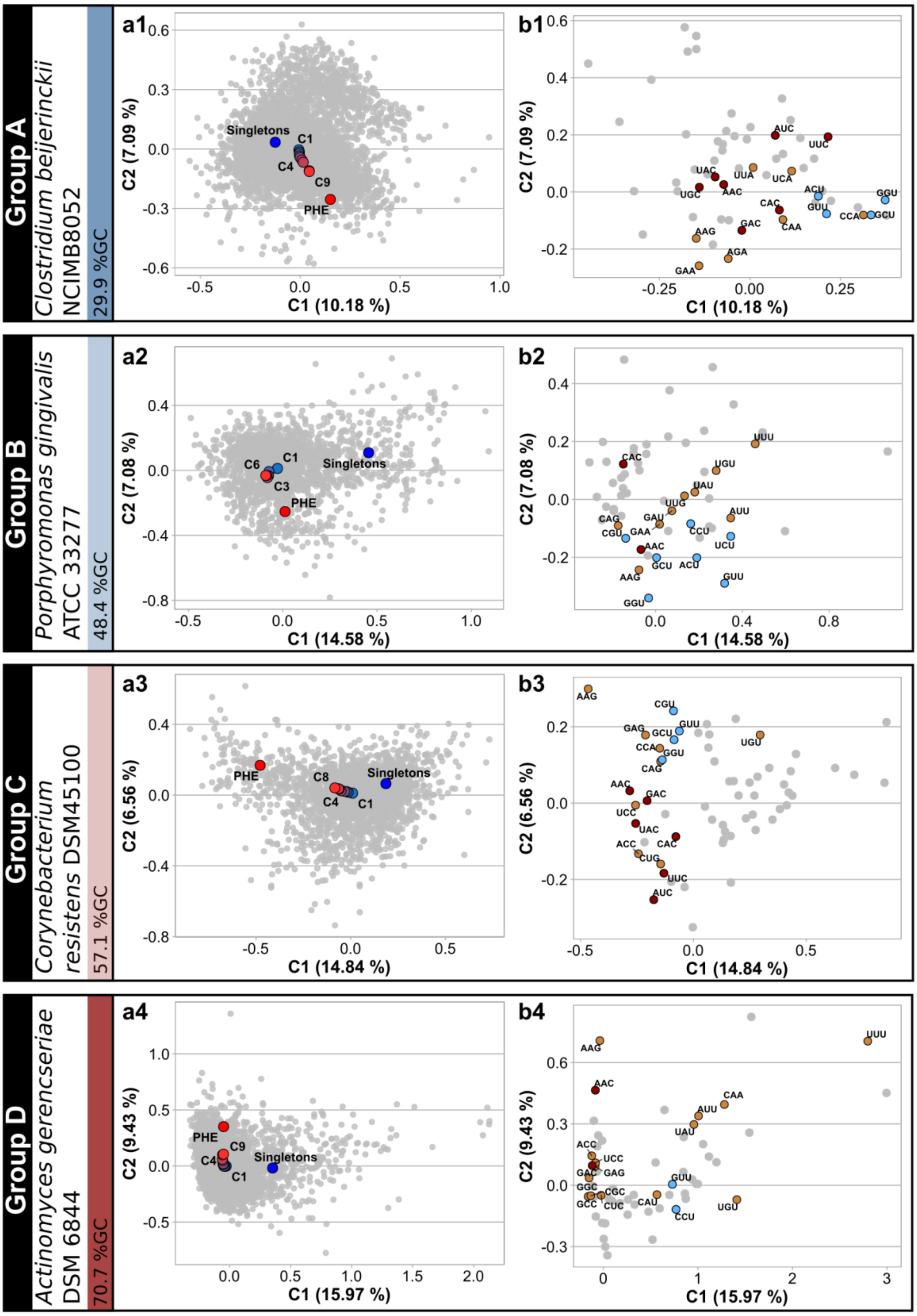

Fig. S2. RCC-based CA plots of core-gene sets excluding the PHE genes. Panels a1 to a4. PHE genes were extracted from each group of core genes and the resulting gene sets, indicated as Ci (from “1” to “n”, in blue to red circles), were projected on the CA plots. Singletons and PHE are in blue and red, respectively. **Panels b1 to b4.** Plots

describing codon relative weight in the first two principal-component positions of the CA. Codons with the highest CUF enrichment for each amino acid from C1 to PHE (i.e. those codons that better represent translational adaptation) were colored in light brown, except when those same codons corresponded also to a 2-/3-fold C- or to a 4-fold U-bias in which cases they were colored in dark brown and light blue, respectively. Such C-bias and U-bias indicate that 2-/3-fold degenerate amino acids are biased towards the use of C-ending codon, and that 4-fold degenerate amino acids are biased towards the use of U-ending codon, respectively.
