# Supplementary material for: Codon Usage Optimization in the Prokaryotic Tree of Life: How Synonymous Codons Are Differentially Selected in Sequence Domains with Different Expression Levels and Degrees of Conservation
Source: mBio. 2020 Jul 21;11(4):e00766-20. doi: 10.1128/mBio.00766-20 (PMC7374057; doi:10.1128/mBio.00766-20)
Supplement: FIG S3 [file mBio.00766-20-sf003.pdf]

**FIGURE S3A**

(a)

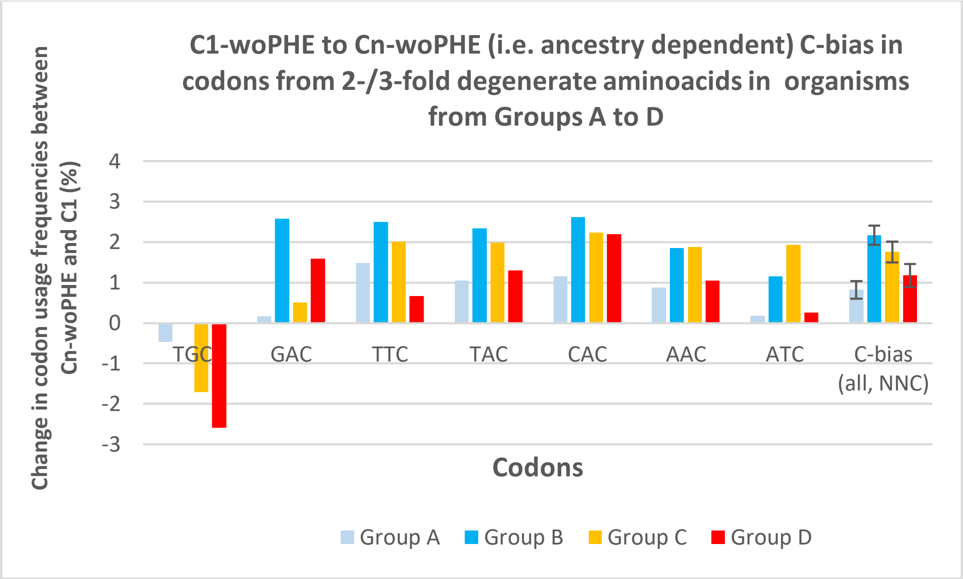


(b)

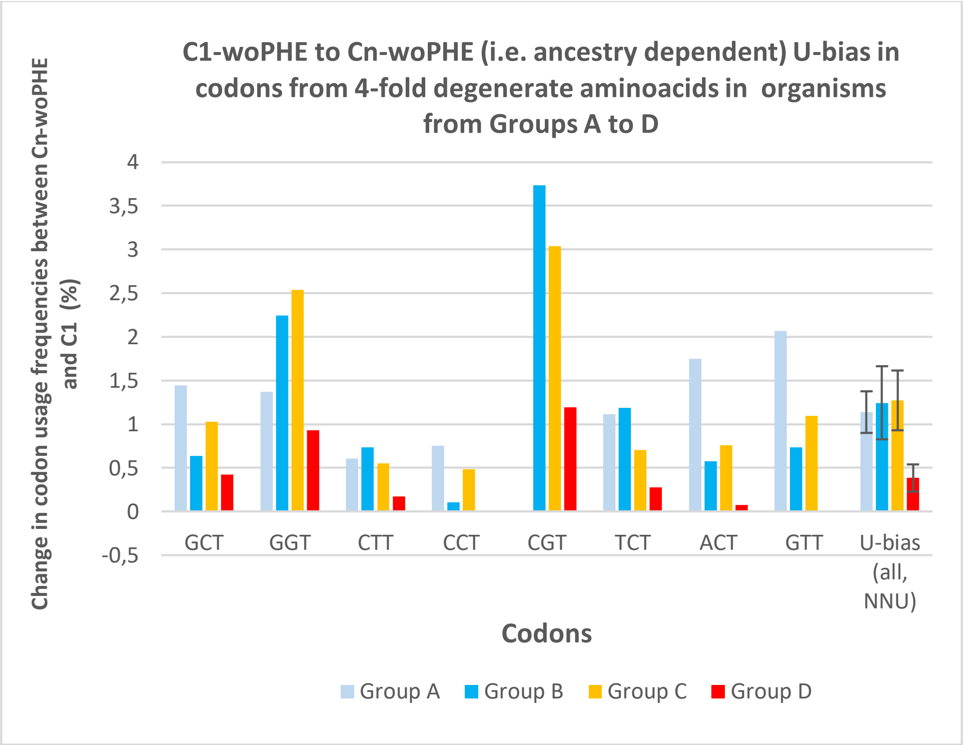


**Fig. S3A. Ancestry-dependent C- and U- bias when comparing the codon usage in the gene sets C1-woPHE and Cn-woPHE (i.e. Cn without PHE) in organisms from Groups A to D. (a)** C-bias at the third position of codons in 2-/3-fold degenerate aminoacids (significant bias in all groups with *t*-test at $p<0.02$). **(b)** U-bias at the third position of codons in 4-fold degenerate aminoacids (significant bias in all groups with *t*-test at $p<0.05$). Average values and standard deviations (SD) are shown in each panel on the right side considering all codons together (except TGC for Cys in panel a).

**FIGURE S3B**

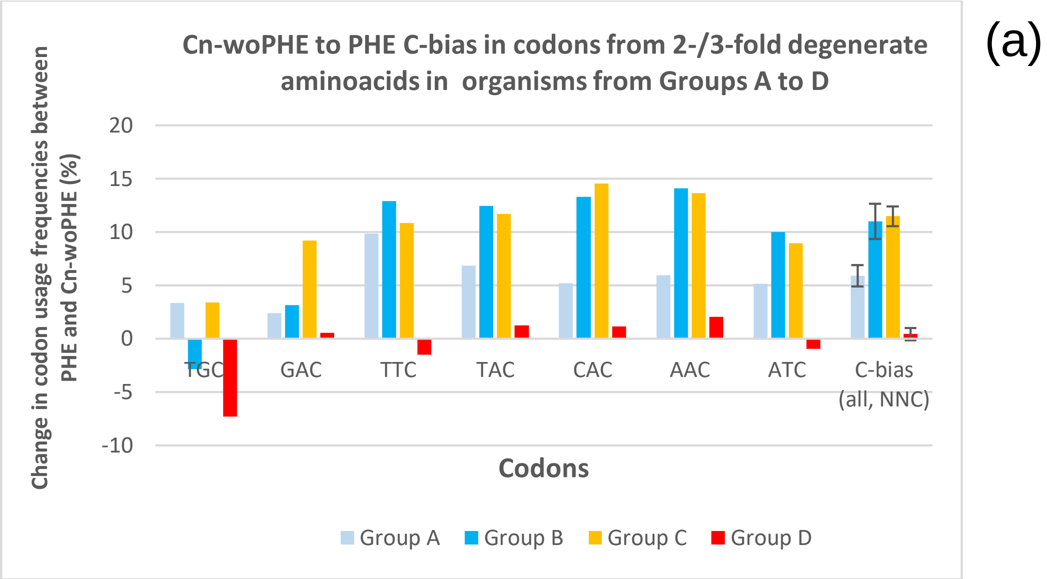


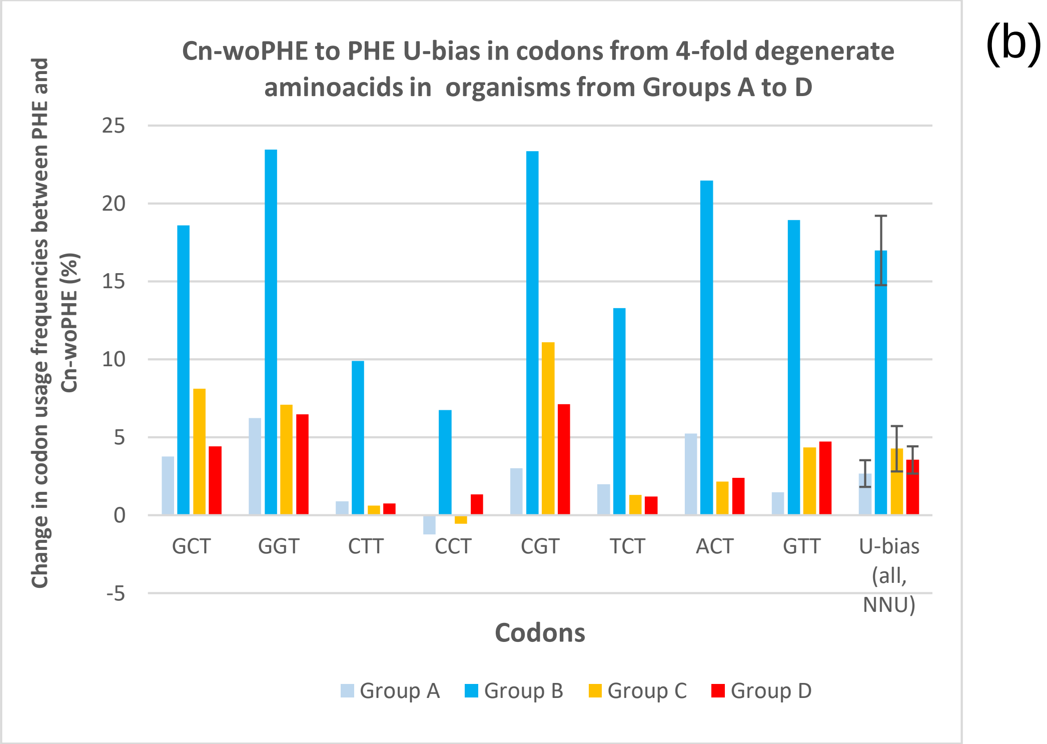


**Fig. S3B. Ancestry-dependent C- and U- bias when comparing the codon usage in the gene sets Cn-woPHE (i.e. Cn without PHE) and PHE in organisms from Groups A to D. (a)** C-bias at the third position of codons in 2-/3-fold degenerate aminoacids (significant bias in groups A to C with *t*-test at $p<0.002$). **(b)** U-bias at the third position of codons in 4-fold degenerate aminoacids (significant bias in all groups with *t*-test at $p<0.03$). Average values and standard deviations (SD) are shown in each panel on the right side considering all codons together (except TGC for Cys in panel a).
