# Supplementary material for: Codon Usage Optimization in the Prokaryotic Tree of Life: How Synonymous Codons Are Differentially Selected in Sequence Domains with Different Expression Levels and Degrees of Conservation
Source: mBio. 2020 Jul 21;11(4):e00766-20. doi: 10.1128/mBio.00766-20 (PMC7374057; doi:10.1128/mBio.00766-20)
Supplement: FIG S4 [file mBio.00766-20-sf004.pdf]

Fig. S4

Group A

Methanobrevibacter smithii ATCC 35061

a1

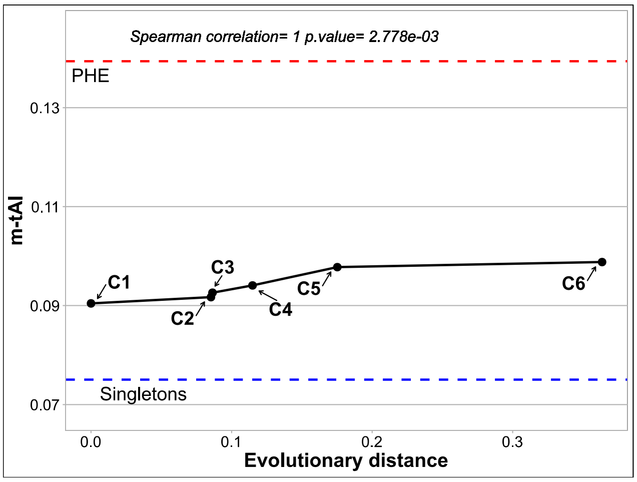

b1

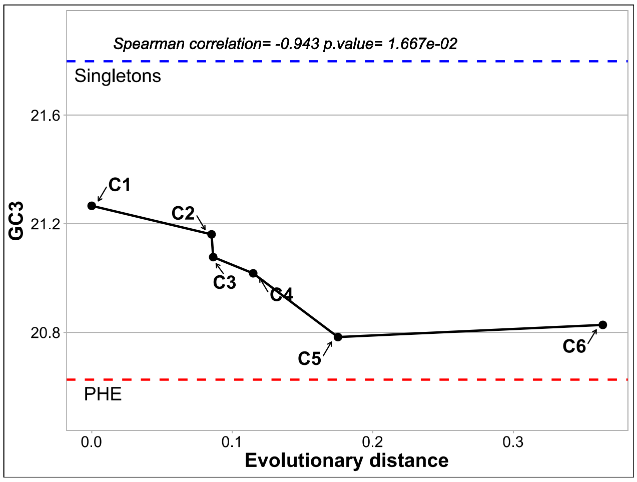

Tetragenococcus halophilus NBRC 12172 NC_016052

a2

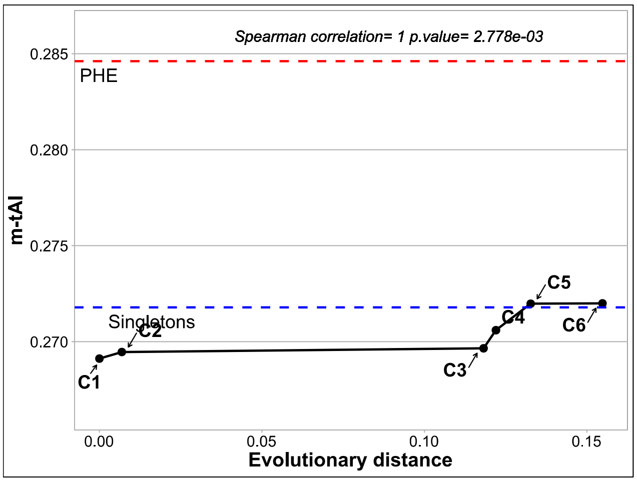

b2

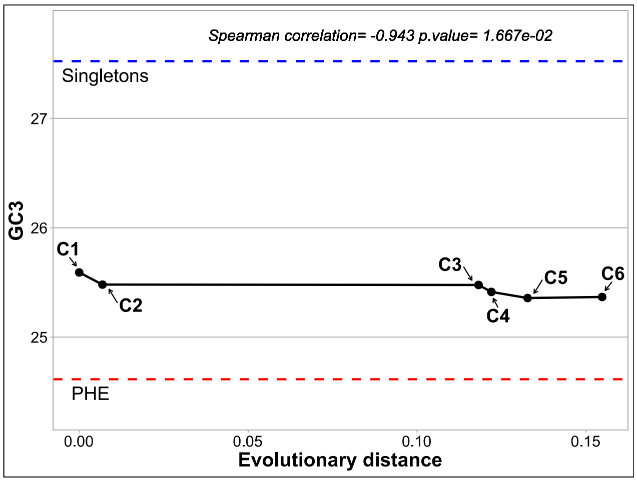

Group B

Sulfurospirillum multivorans DSM 12446 NZ_CP007201

a3

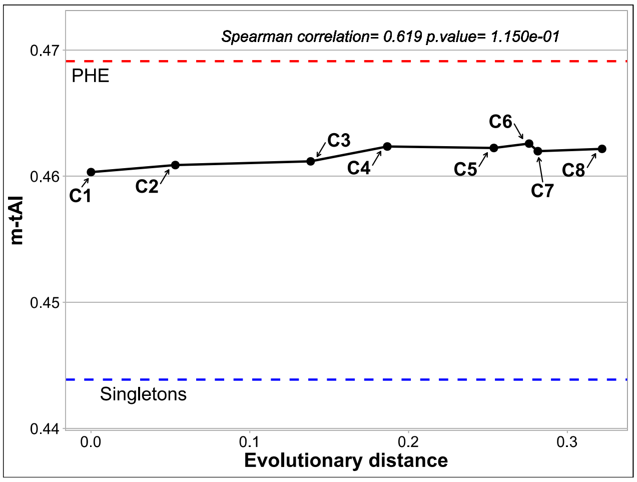

b3

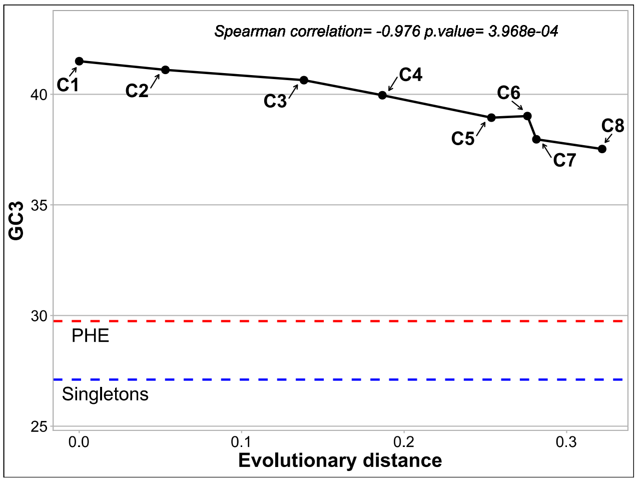

Streptococcus equi ATCC 33398 NZ_FTNH01000046

a4 **b4**

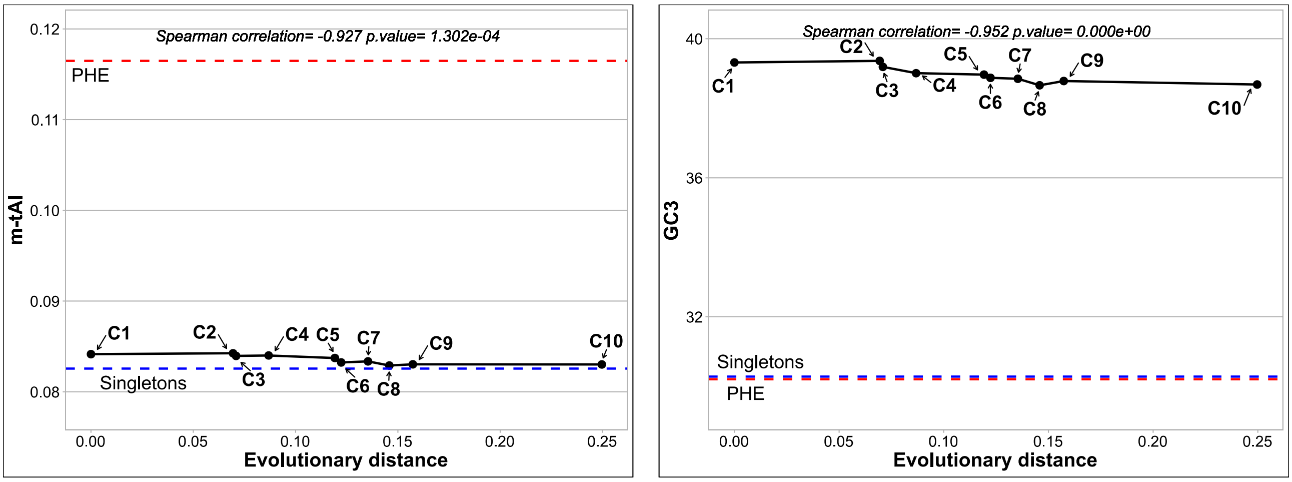

Bacteroides vulgatus ATCC 8482 NC_009614

a5 **b5**

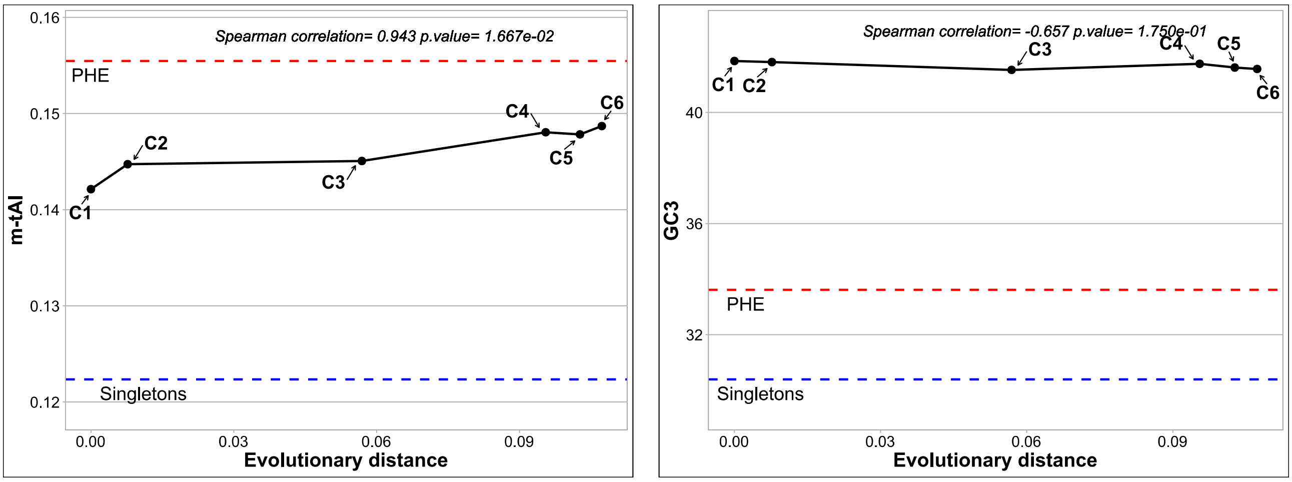

Bacillus subtilis subsp spizizenii TU B 10 NC_016047

a6 **b6**

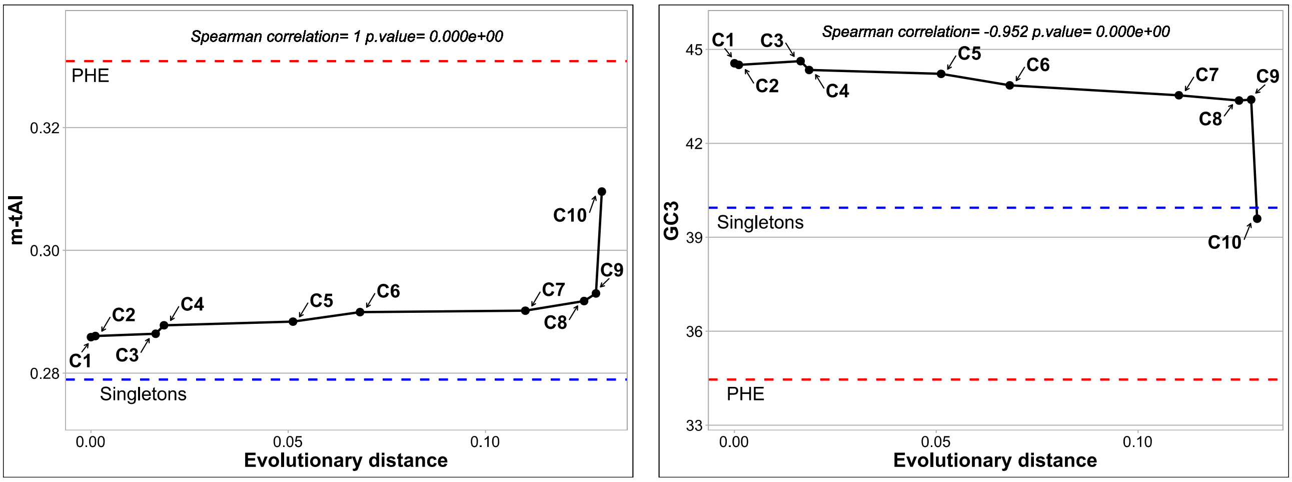

Moraxella bovis CCUG 2133 NZ_MUXV01000108

a7

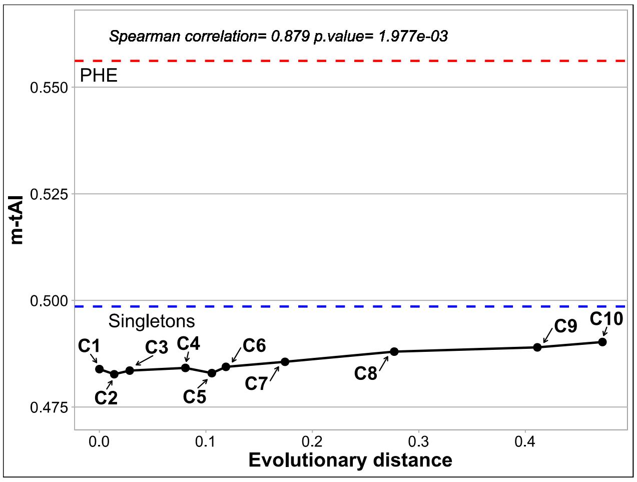

b7

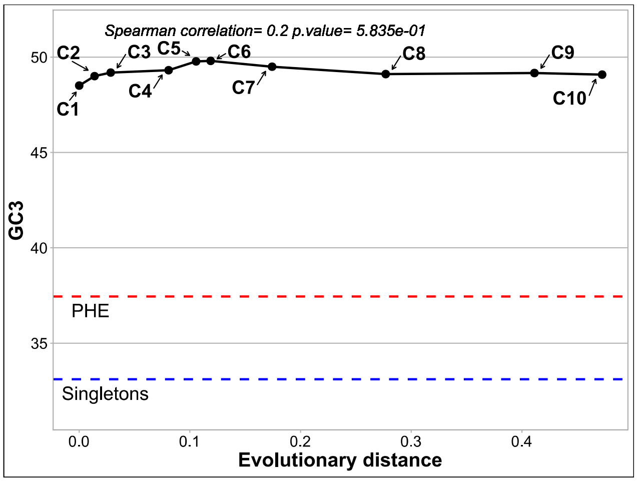

Chromobacterium violaceum ATCC12472

a8

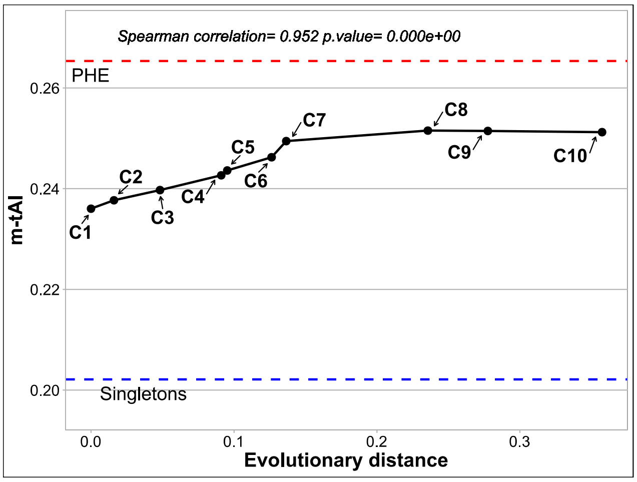

b8

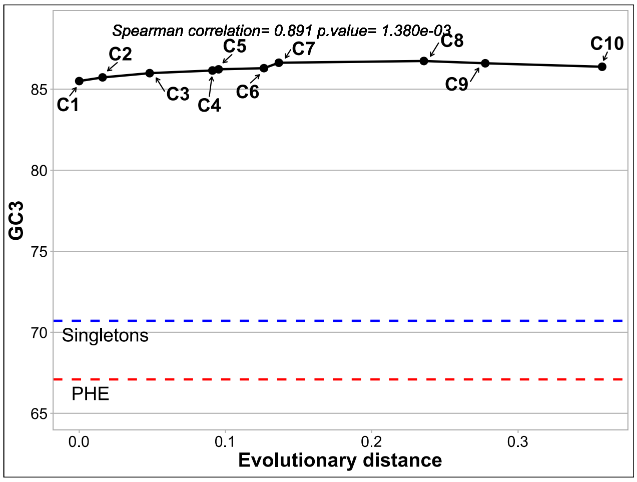

Paenibacillus graminis DSM 15220 NZ_CP009287

a9

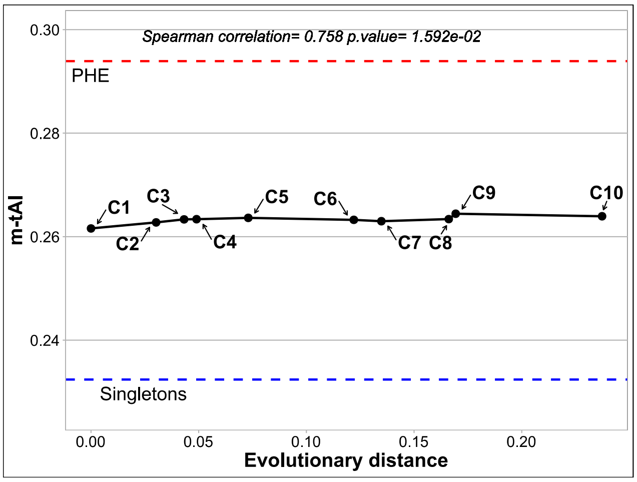

b9

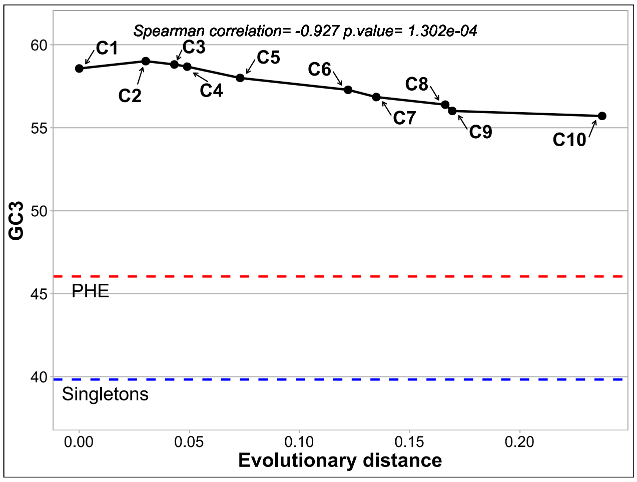

Photobacterium gaetbulicola Gung47

a10

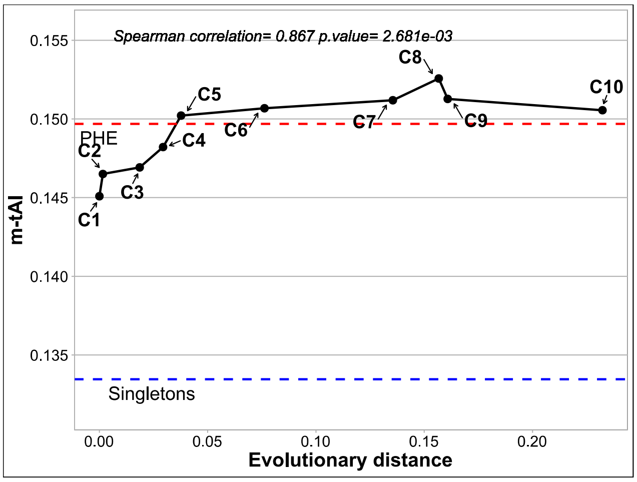

b10

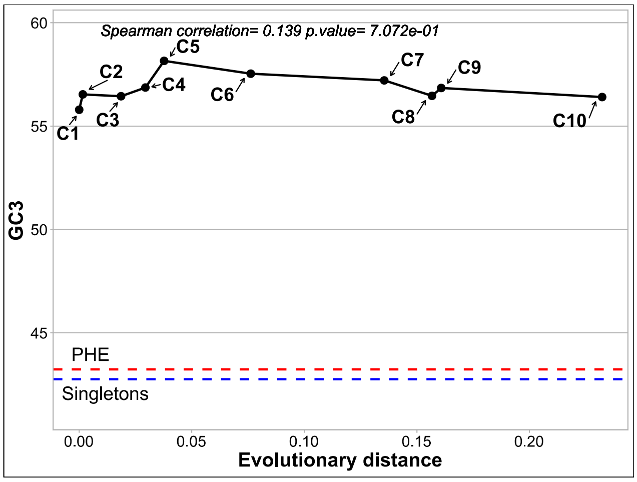

Group C

Treponema succinifaciens DSM 2489 NC_015385

a11

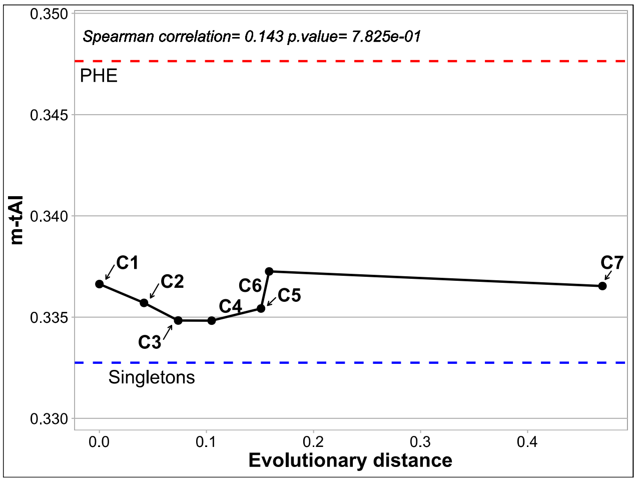

b11

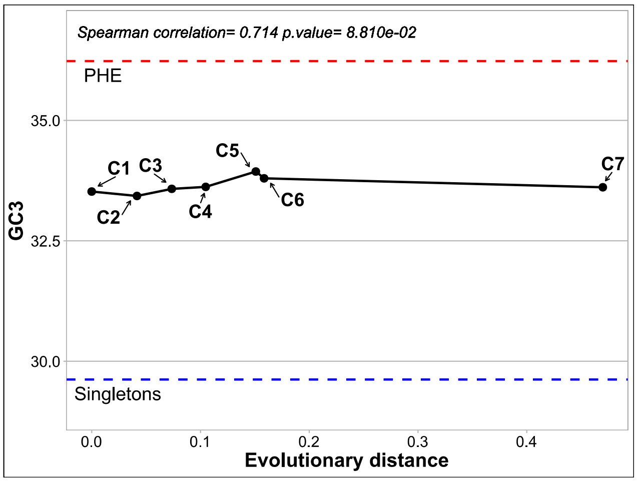

Prevotella melaninogenica ATCC 25845 NC_014371

a12

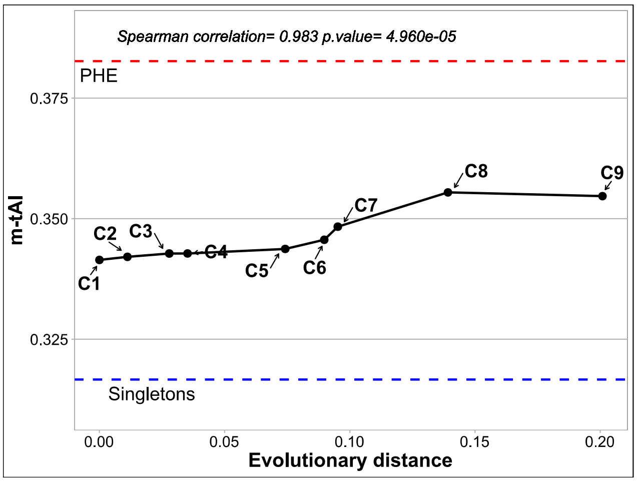

b12

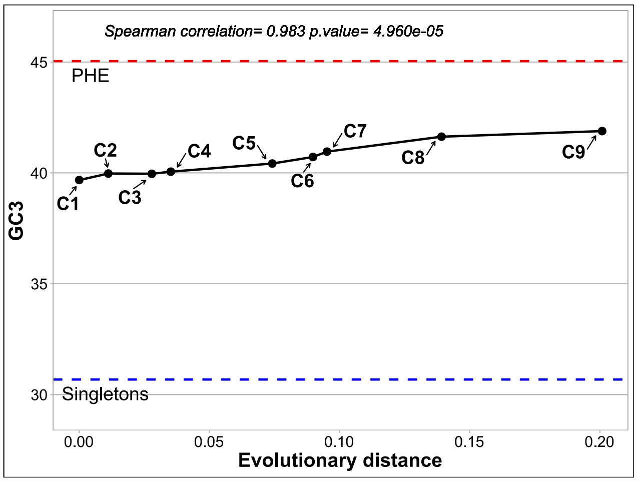

Yersinia enterocolitica subsp palearctica Y11 NC_017564

a13

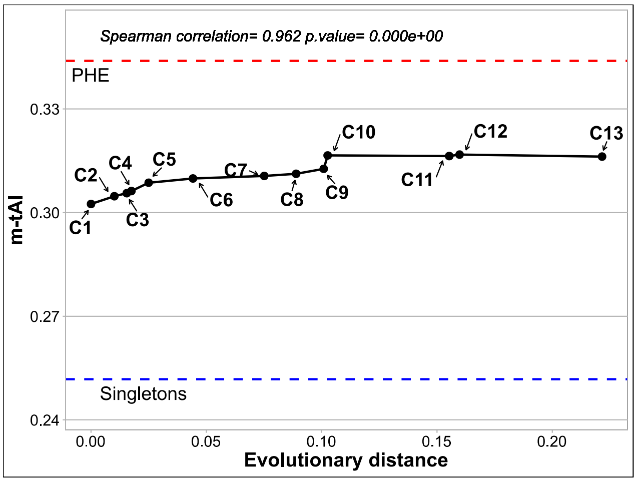

b13

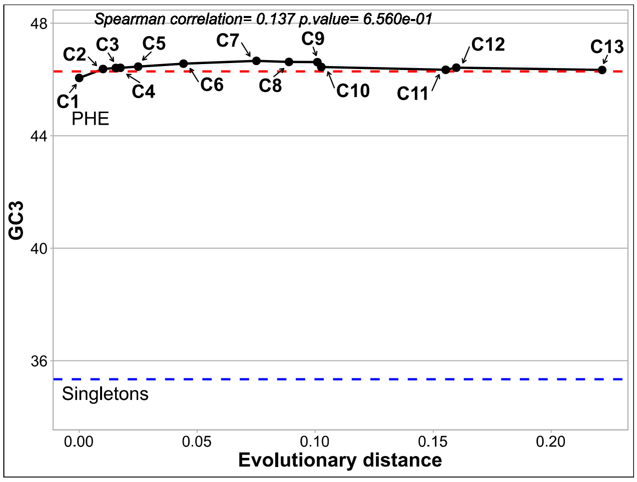

Methanolacinia petrolearia DSM 11571 NC_014507

a14

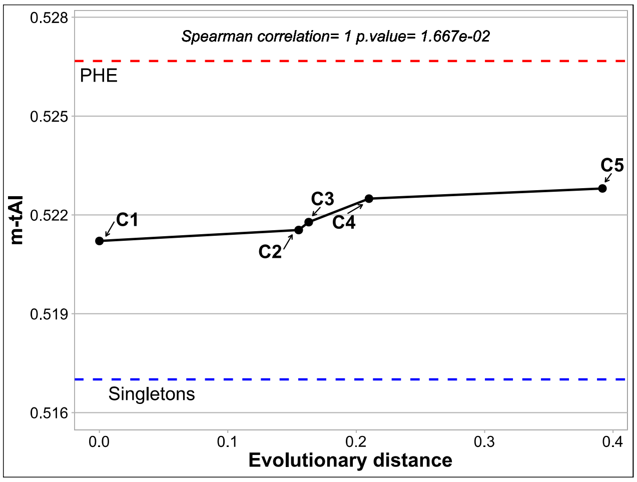

b14

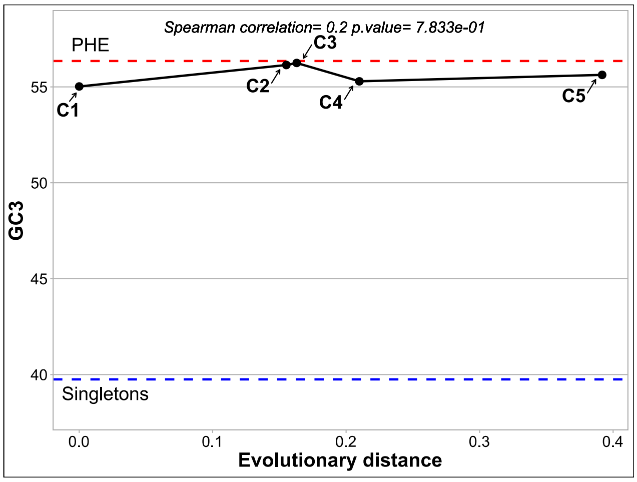

Bifidobacterium longum subsp longum JCM 1217 NC_015067

a15

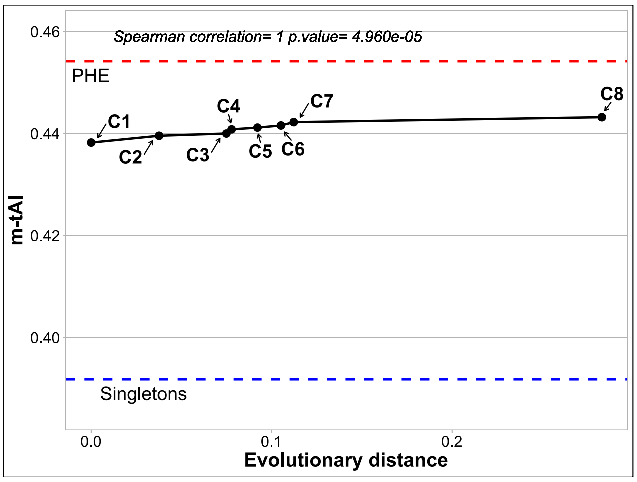

b15

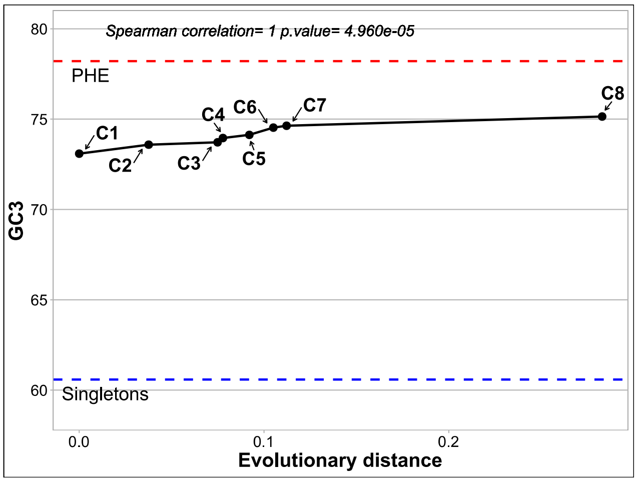

Bordetella holmesii ATCC 51541 NZ_CP007494

a16

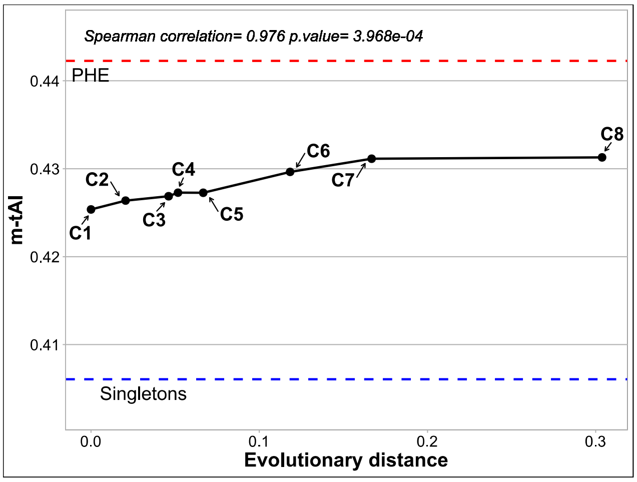

b16

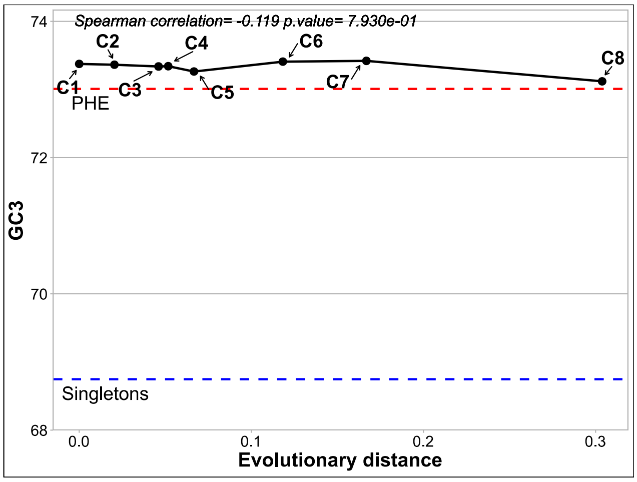

Mycobacterium fortuitum subsp fortuitum DSM 46621 ATCC 6841 CP014258

a17

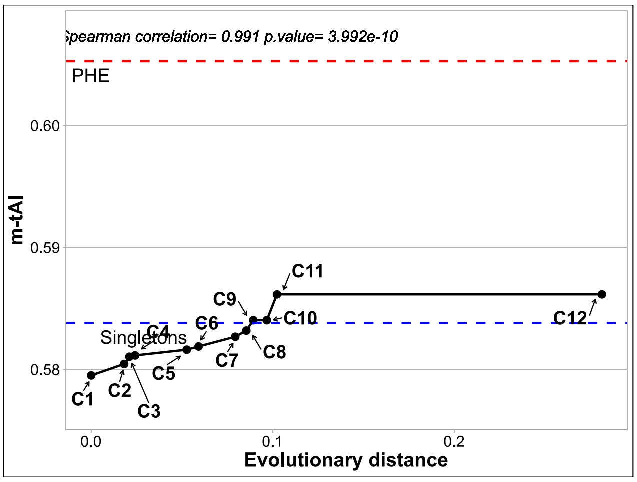

b17

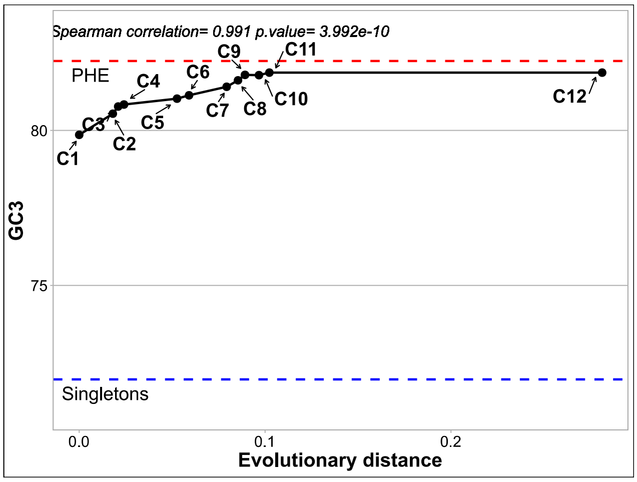

Sphingomonas parapaucimobilis NBRC 15100 NZ_BBPI01000001

a18

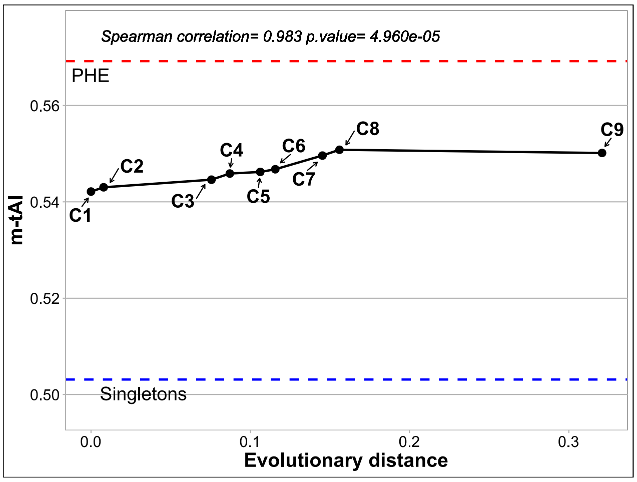

b18

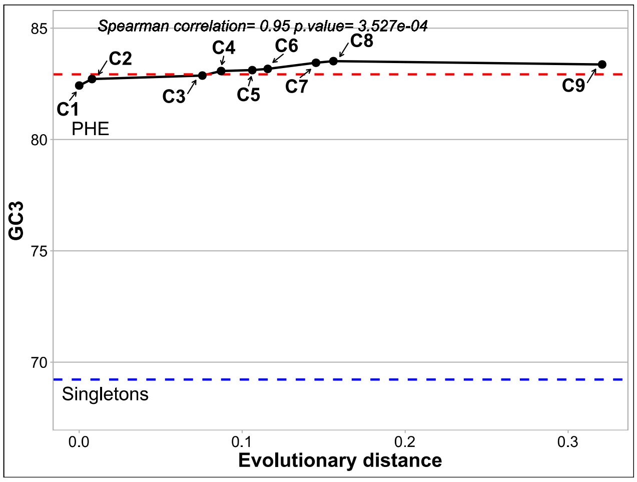

Atopobium parvulum DSM20469

a19

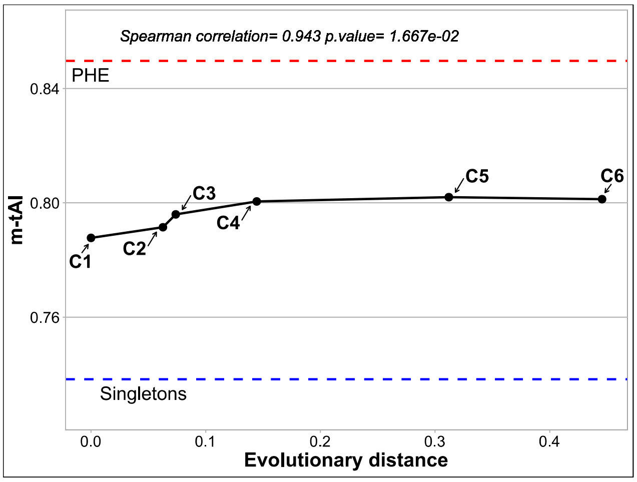

b19

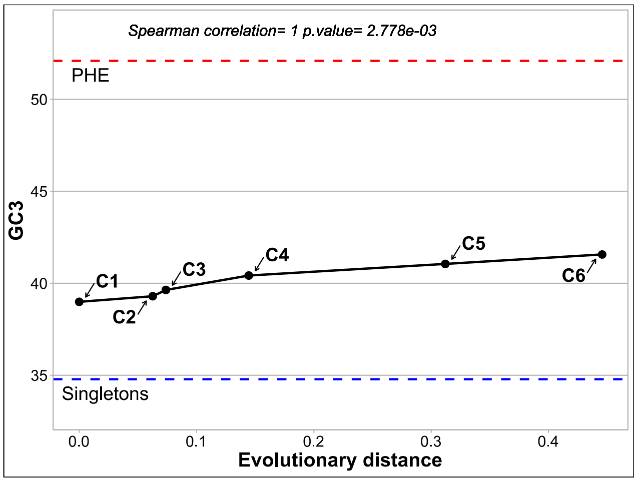

Group D

Leisingera methylohalidivorans DSM 14336

a20

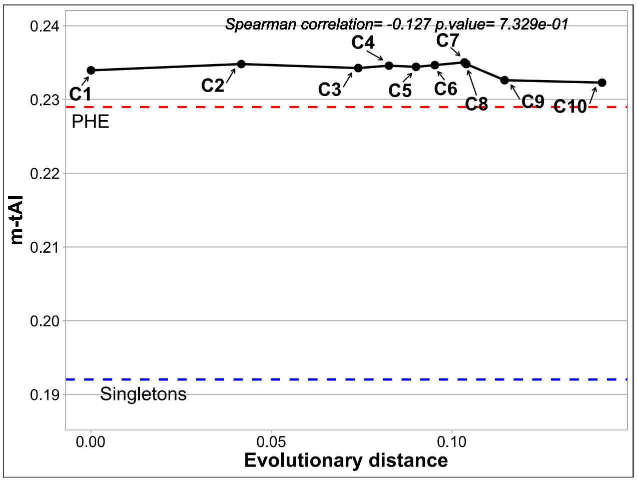

b20

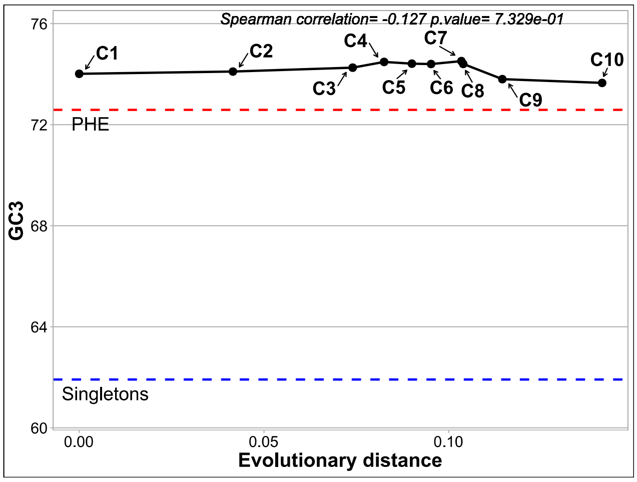

Oceanibaculum pacificum MCCC 1A02656 NZ_LPXN01000181

a21

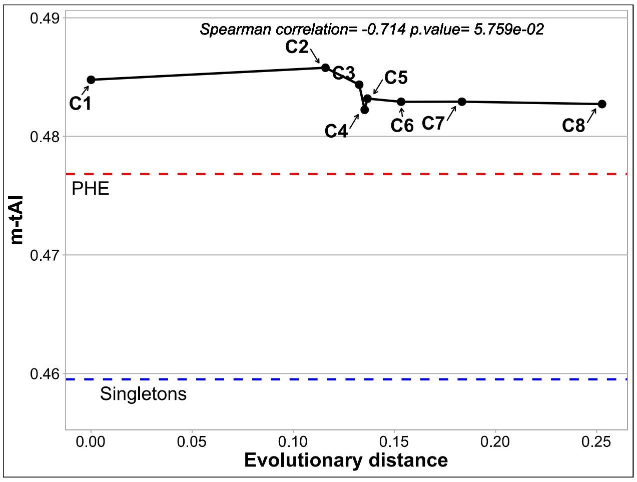

b21

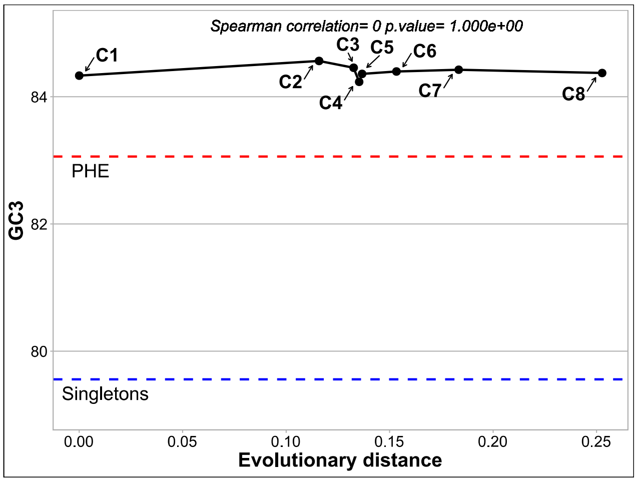

Arthrobacter enclensis NIO 1008 NZ_KQ758616

a22

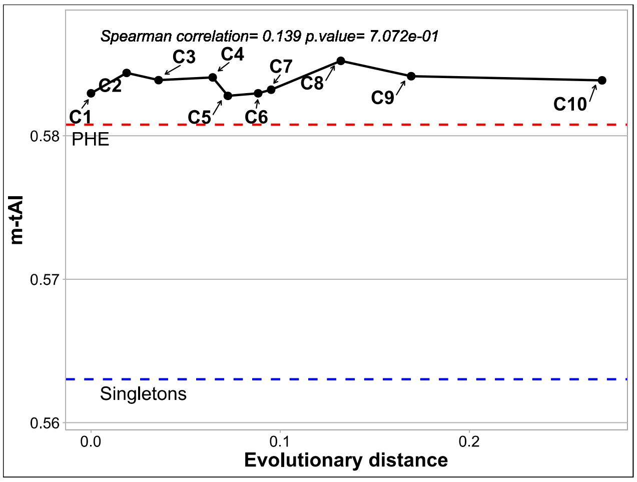

b22

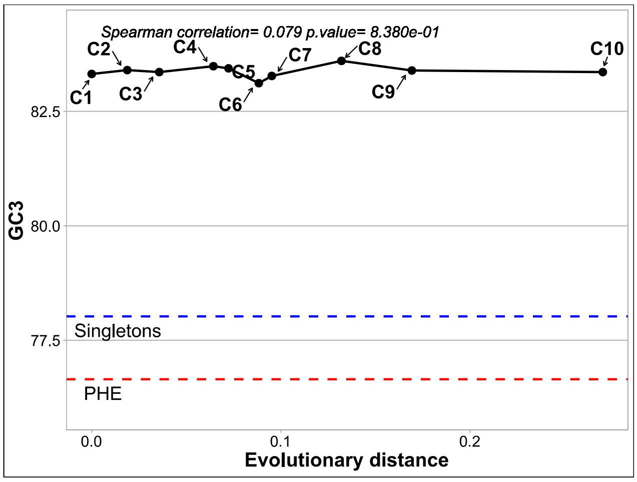

Rhodanobacter denitrificans 2APBS1

a23

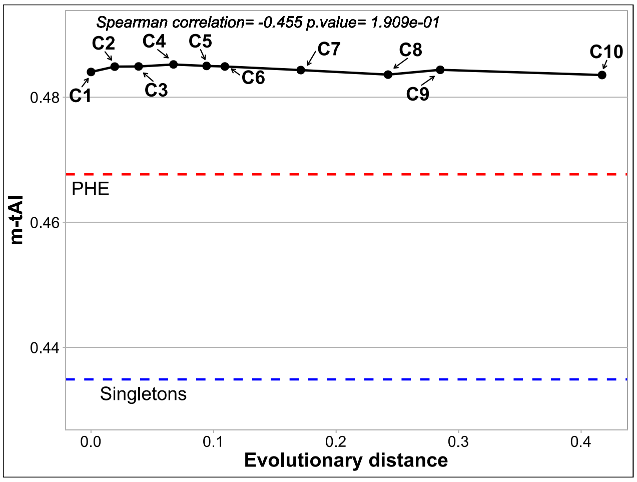

b23

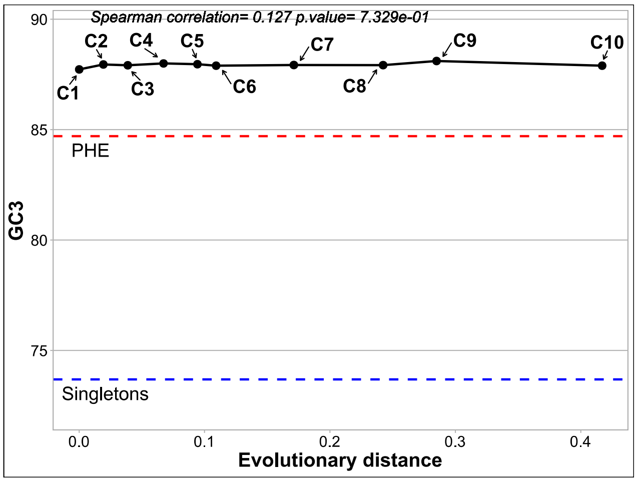

Microbacterium aurum KACC 15219 NZ_CP018762

a24

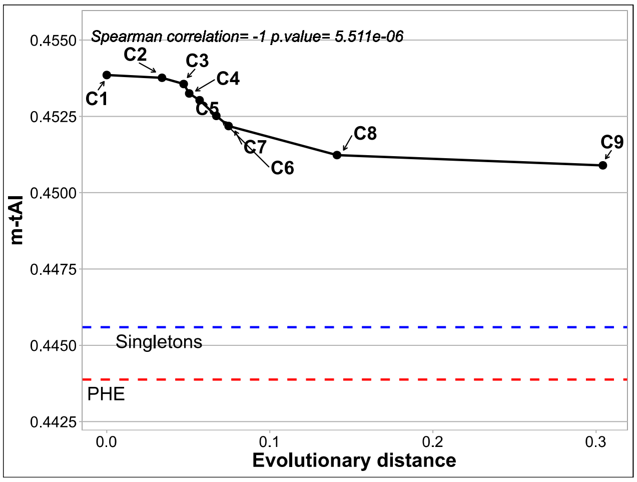

b24

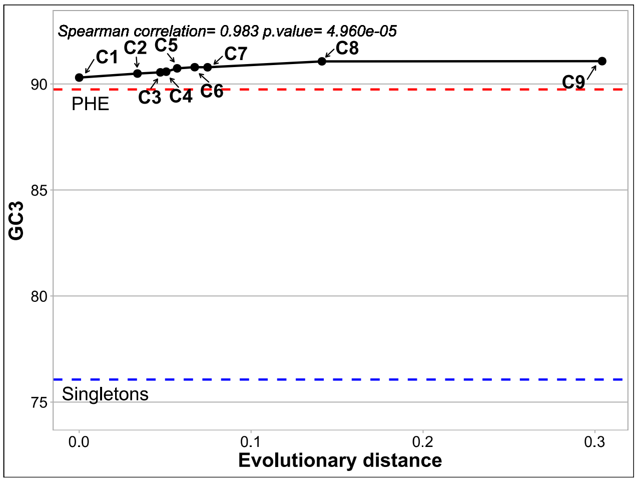

Roseomonas mucosa ATCC BAA 692 NZ_JHWD01000081

a25

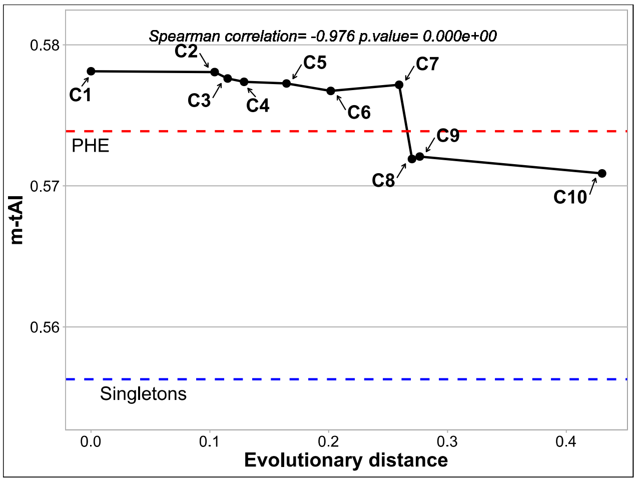

b25

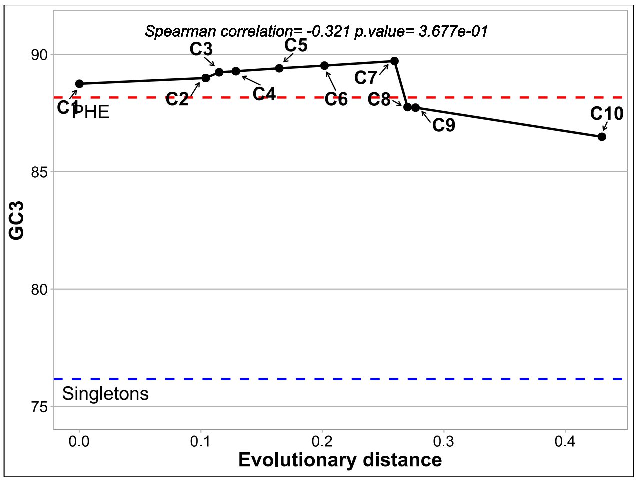

Fig. S4. Codon-usage adaptations to the cellular tRNA-pool, and changes in the GC3 content of core-gene sets with different degrees of conservation throughout the phylogeny of 25 prokaryote families. The strains represented here are the same as the 25 in Fig. S1. **Panels a1 to a25.** In each figure, the modal tRNA-adaptation index (m-tAI) calculated for each of the Ci gene sets as described in Materials and Methods is plotted on the *ordinate* as a function of the evolutionary distance (Table S1_a, tab 2) indicated on the *abscissa* as inferred from the corresponding phylogenetic trees included in Table S1_a-c. Higher values of m-tAI indicate an enrichment in the codon-usage frequencies of those synonymous codons better adapted to the host-cell tRNA pool. The C1 to Cn gene sets plotted here are the same as those presented in Fig. 1. The red and blue horizontal dashed lines correspond to the respective m-tAI values calculated for the PHE genes and the singletons. **Panels b1 to b25.** In each panel, the average GC3 content in each core-gene set of increasing ancestry is plotted on the *ordinate* as a function of the evolutionary distance indicated on the *abscissa* as in Panels a1 to a25. The PHE genes and the singletons are represented as red and blue horizontal dashed lines, respectively.
