# Supplementary material for: Codon Usage Optimization in the Prokaryotic Tree of Life: How Synonymous Codons Are Differentially Selected in Sequence Domains with Different Expression Levels and Degrees of Conservation
Source: mBio. 2020 Jul 21;11(4):e00766-20. doi: 10.1128/mBio.00766-20 (PMC7374057; doi:10.1128/mBio.00766-20)
Supplement: FIG S5 [file mBio.00766-20-sf005.pdf]

Fig. S5A

Group A

S4-1 Methanobrevibacter smithii ATCC 35061

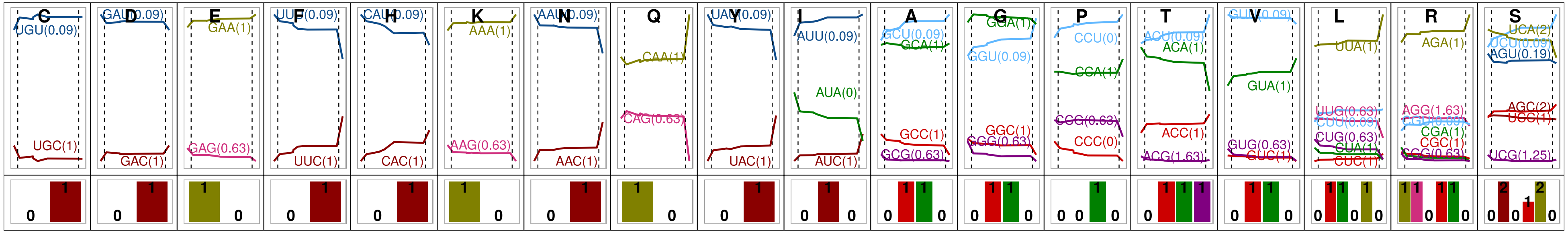

S4-2Tetragenococcus halophilus NBRC 12172 NC_016052

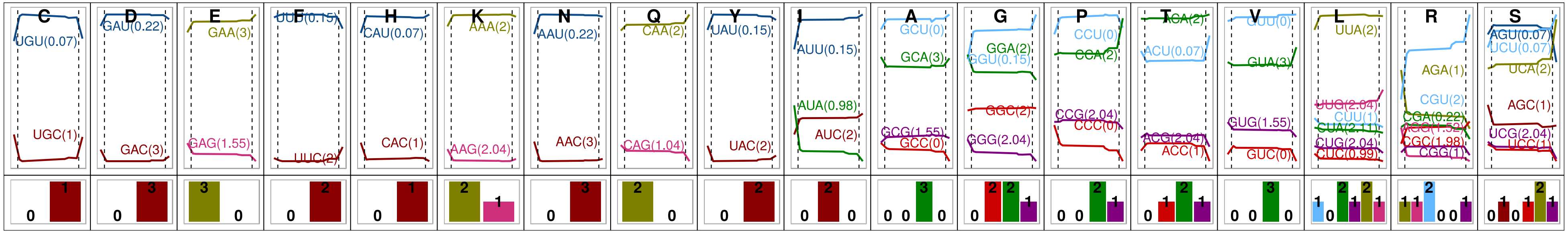

Group B

S4-3 Sulfurospirillum multivorans DSM 12446 NZ_CP007201

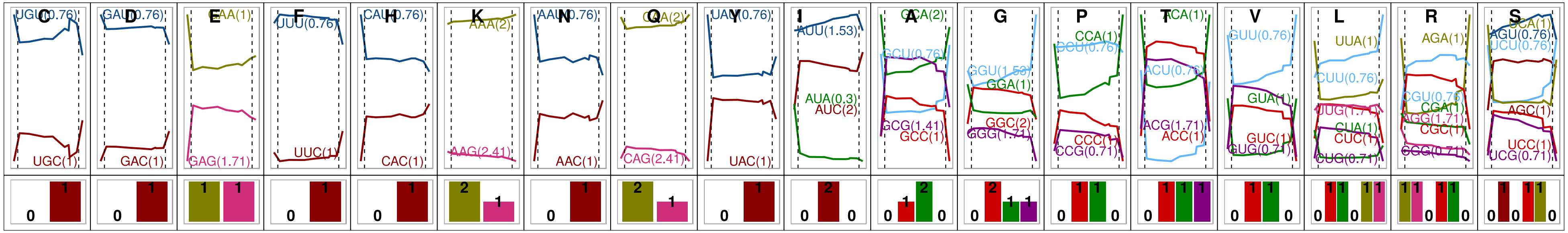

S4-4 Streptococcus equi ATCC 33398 NZ_FTNH01000046

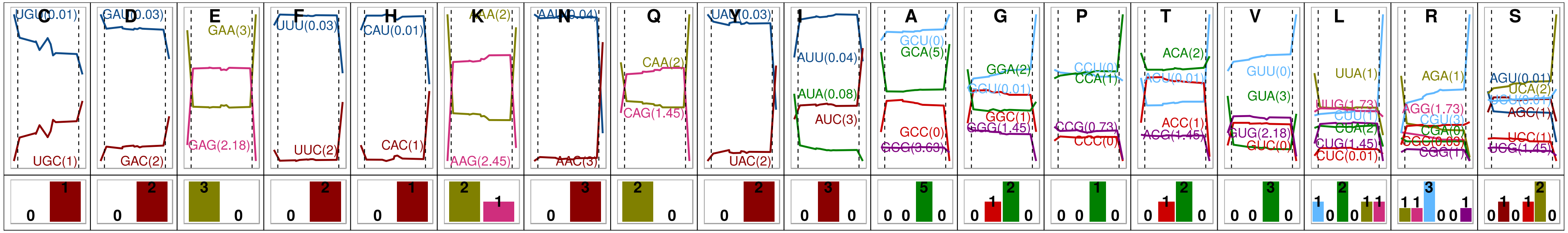

S4-5 Bacteroides vulgatus ATCC 8482 NC_009614

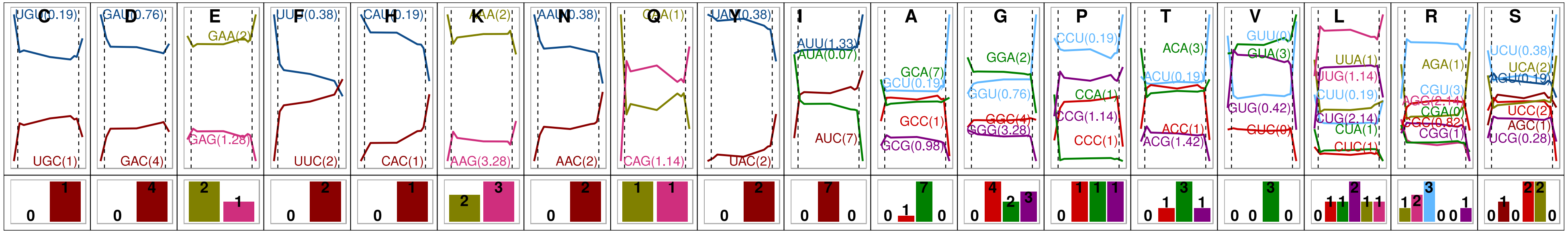

S4-6 Bacillus subtilis subsp spizizenii TU B 10 NC_016047

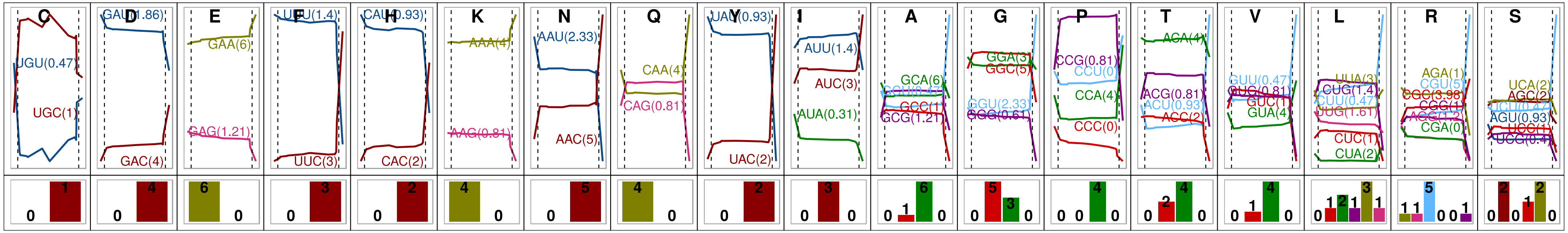

S4-7 Moraxella bovis CCUG 2133 NZ_MUXV01000108

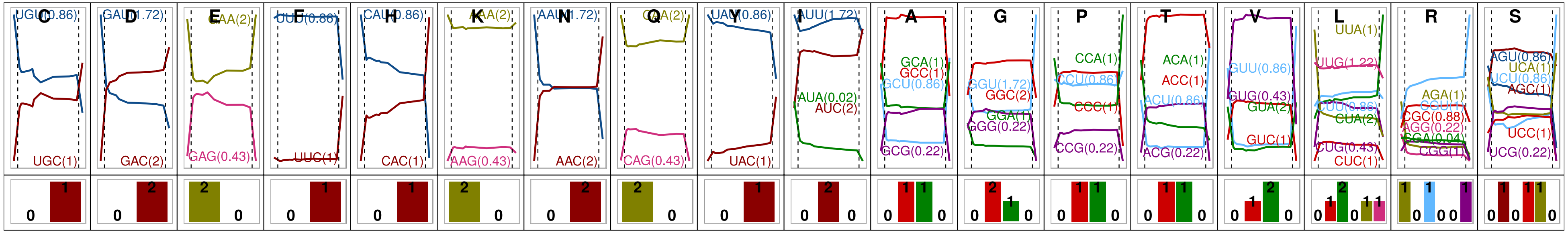

S4-8 Chromobacterium violaceum ATCC12472

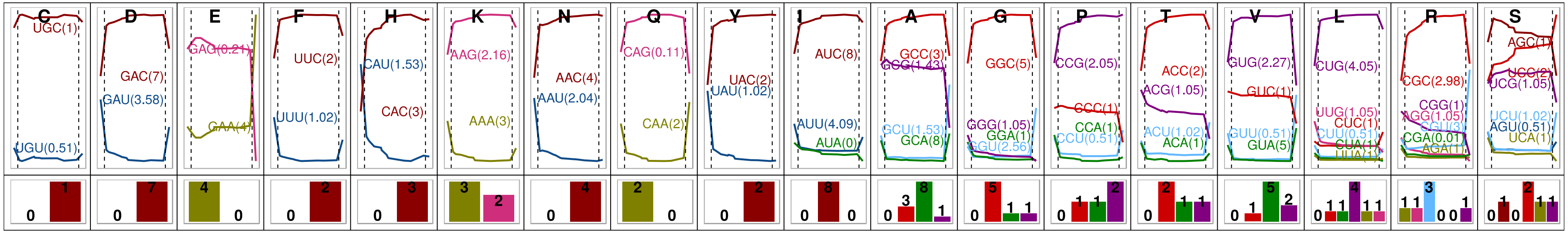

S4-9 Paenibacillus graminis DSM 15220 NZ_CP009287

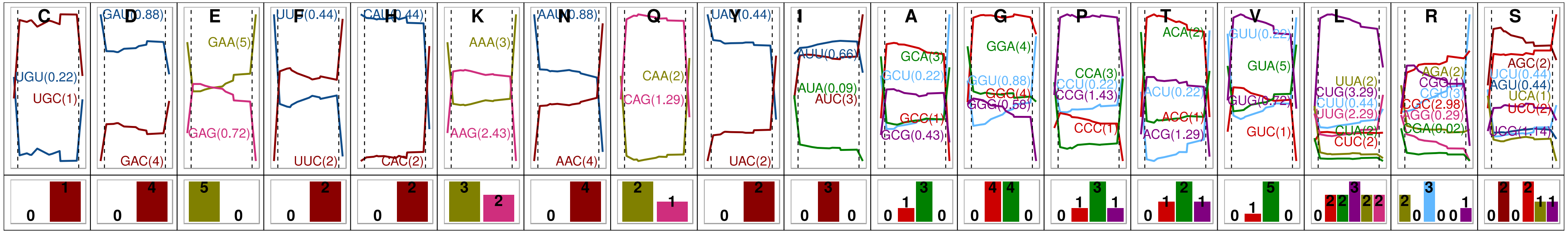

S4-10 Photobacterium gaetbulicola Gung47

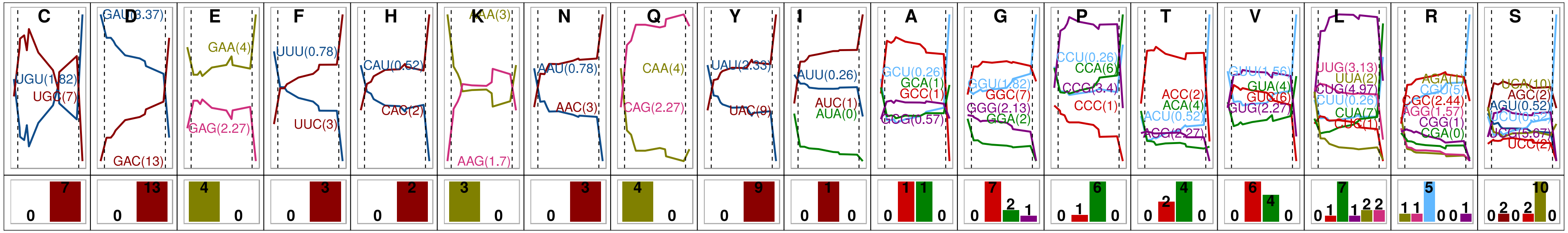

Group C

S4-11 Treponema succinifaciens DSM 2489 NC_015385

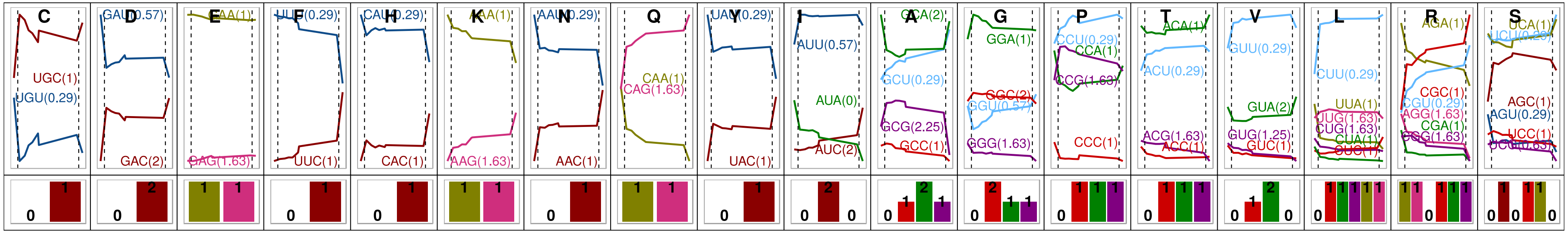

4-12 Prevotella melaninogenica ATCC 25845 NC_014371

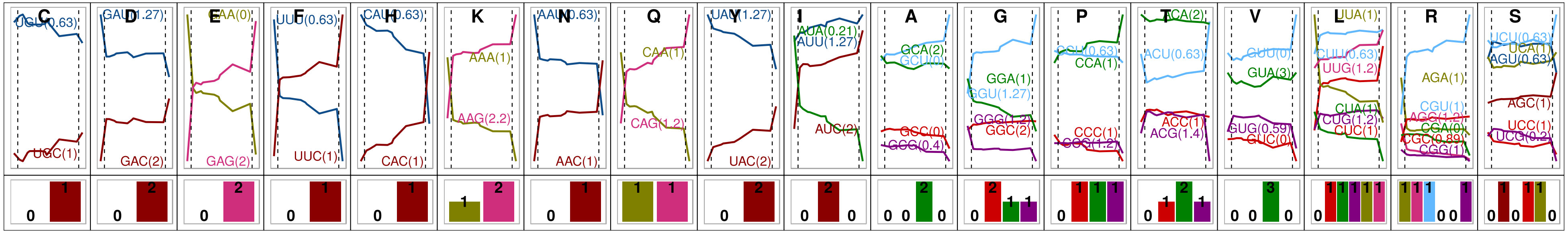

S4-13 Yersinia enterocolitica subsp palearctica Y11 NC_017564

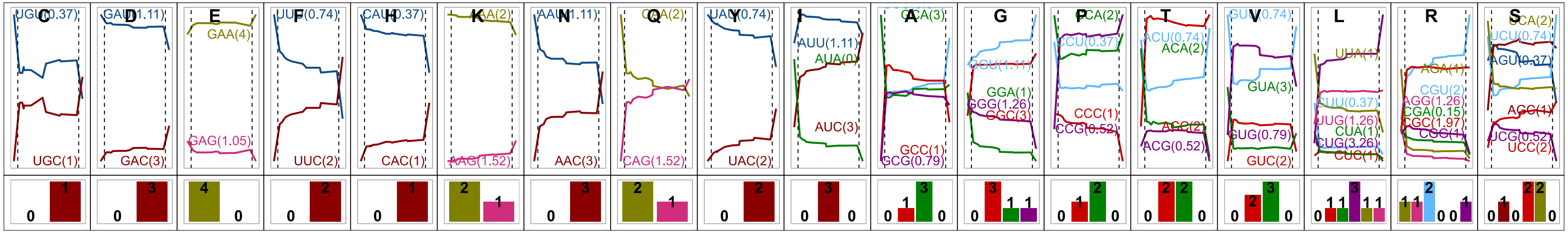

S4-14 Methanolacinia petrolearia DSM 11571 NC_014507

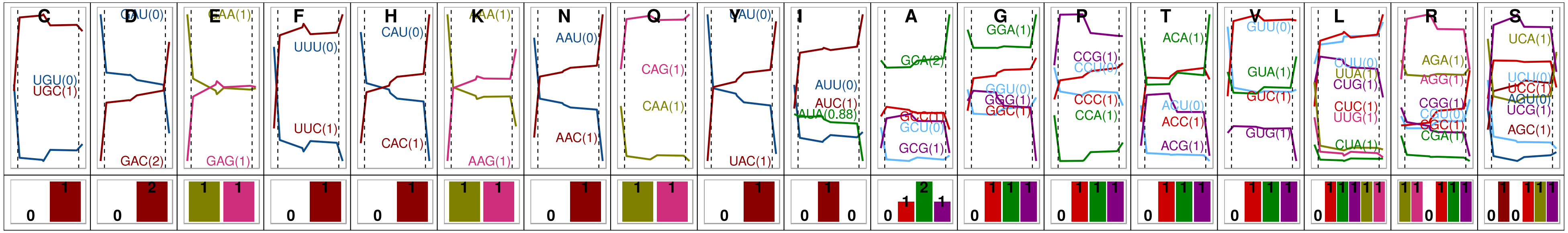

S4-15 Bifidobacterium longum subsp longum JCM 1217 NC_015067

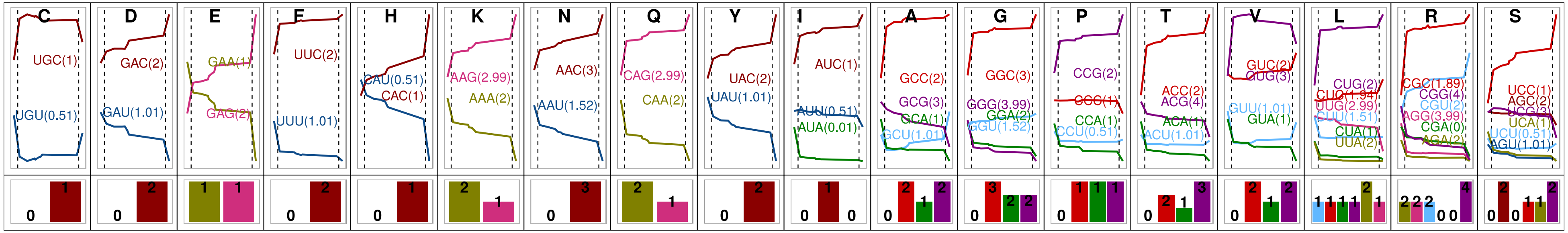

S4-16 Bordetella holmesii ATCC 51541 NZ_CP007494

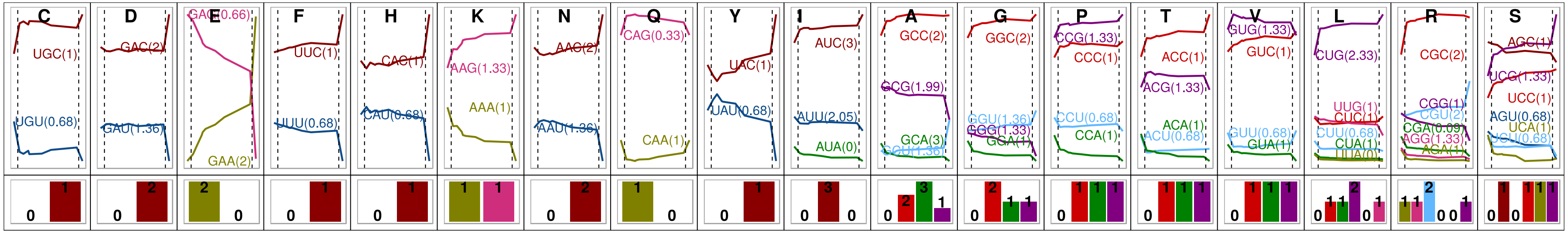

S4-17 Mycobacterium fortuitum subsp fortuitum DSM 46621 ATCC 6841 CP014258

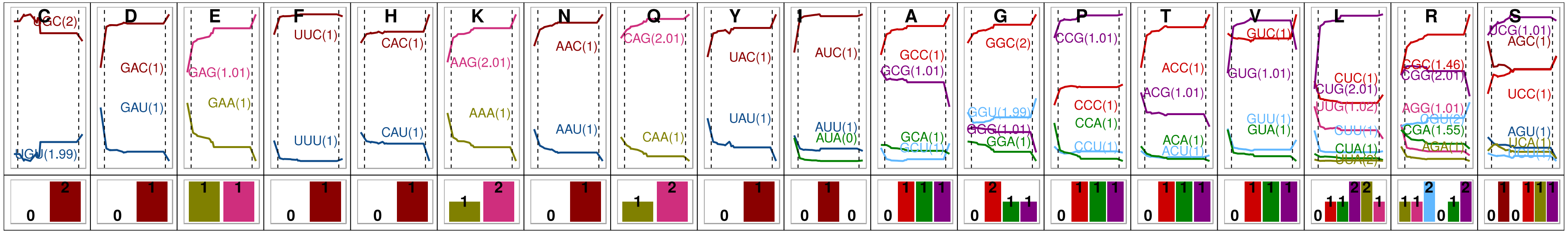

S4-18 Sphingomonas parapaucimobilis NBRC 15100 NZ_BBPI01000001

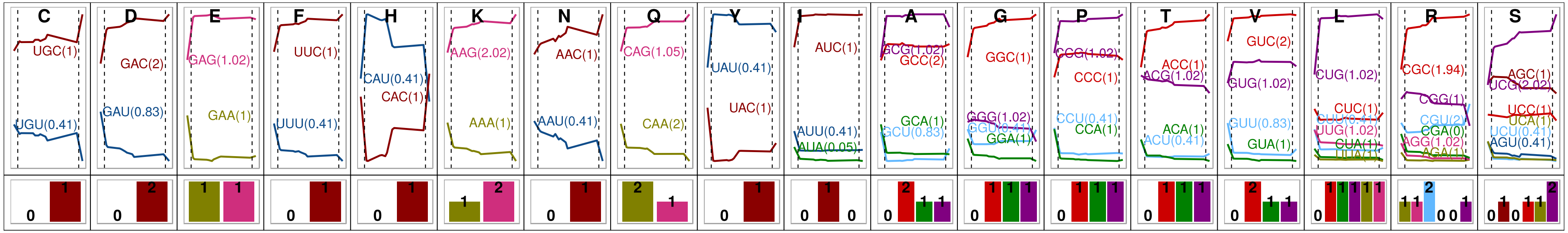

S4-19 Atopobium parvulum DSM20469

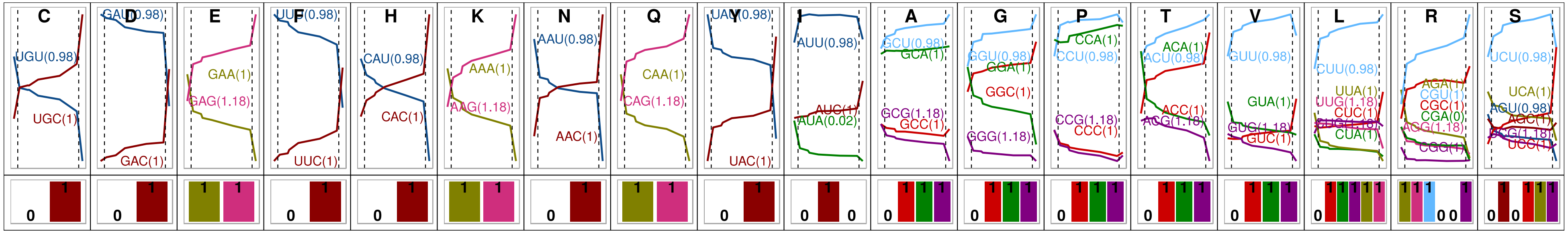

Group D

S4-20 Leisingera methylohalidivorans DSM 14336

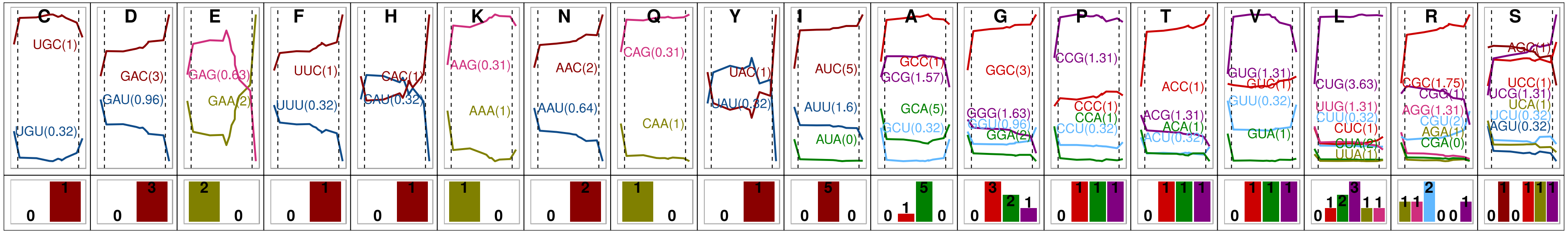

S4-21 Oceanibaculum pacificum MCCC 1A02656 NZ_LPXN01000181

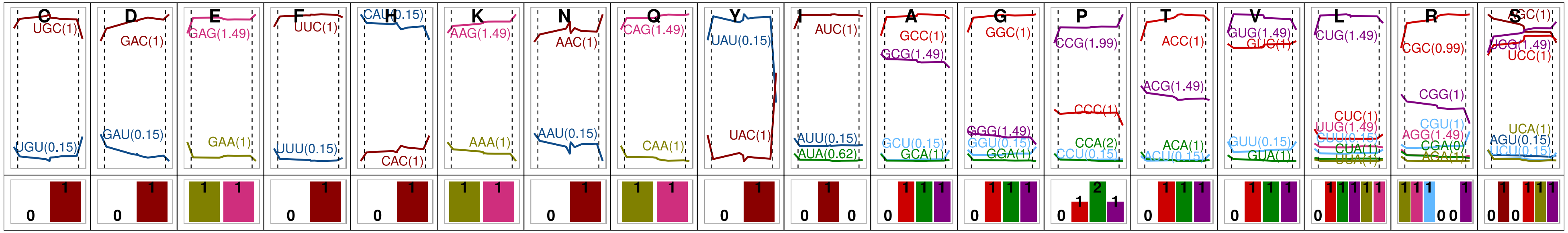

S4-22 Arthrobacter enclensis NIO 1008 NZ_KQ758616

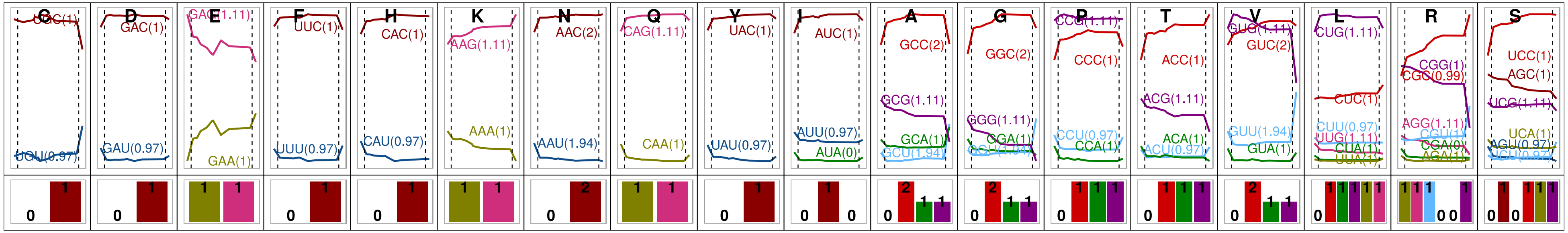

S4-23 Rhodanobacter denitrificans 2APBS1

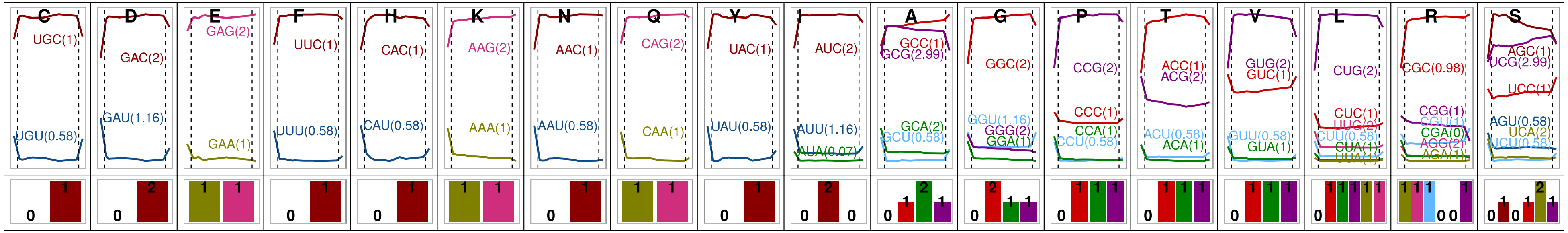

S4-24 Microbacterium aurum KACC 15219 NZ_CP018762

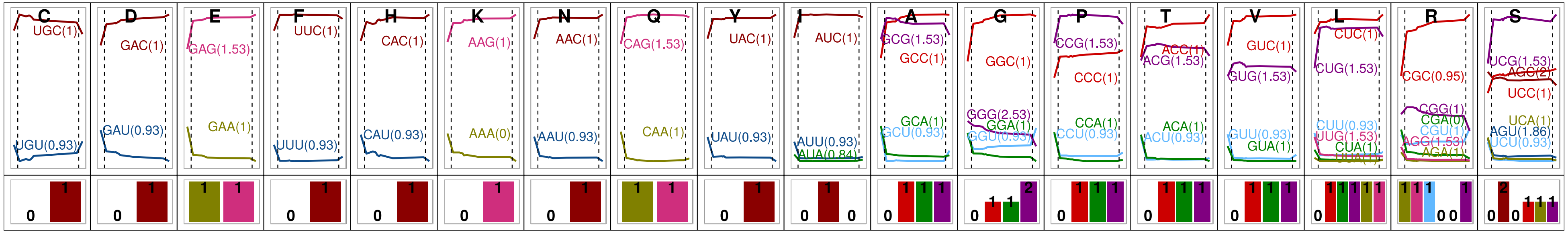

S4-25 Roseomonas mucosa ATCC BAA 692 NZ_JHWD01000081

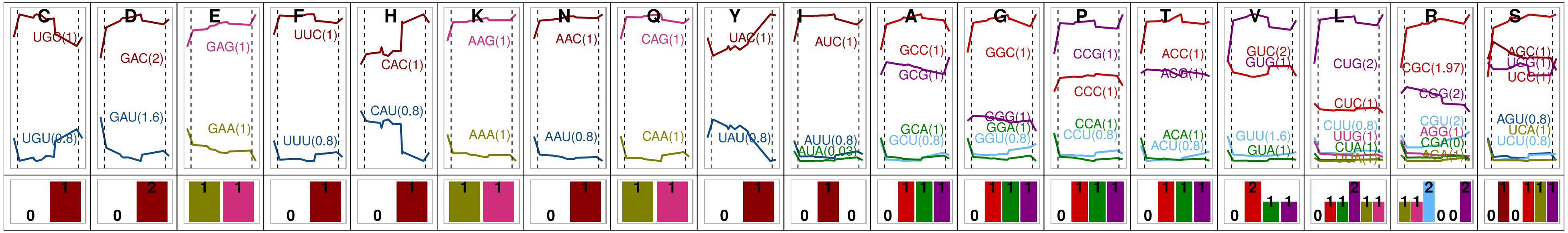

Fig. S5A. Codon usage frequencies and adaptation indices (*Wi*s) of the gene sets analyzed in this work, together with the tRNA-gene–copy numbers for strains of 25 different prokaryote families included in Groups A to D. For the amino acid denoted by the corresponding single-letter identification code located above each panel, the change in the codon-usage frequencies (CUFs; see Materials and Methods) of the core-gene sets with increasing ancestries (left to right, the C1 to Cn), the PHE genes, and the singletons are plotted in the upper panels as solid horizontal curves for each of the indicated codon triplets between the two vertical broken lines, for the singletons to the left of the first of those lines, and for the PHE genes to the right of the second (with singletons and PHE genes being located at the beginning and the end of the curves, respectively). The CUFs are represented by different colors with the associated codon-adaptation index (*Wi*) being denoted within parentheses beside each triplet. Finally, the presence and gene-copy number (N tRNA) of the cognate tRNA species of a given synonymous codon bearing the exact complementary anticodon is depicted with a number and a bar of proportional height in the lower panel in the same color as the corresponding triplet and curve in the upper panel.

FIGURE S5B

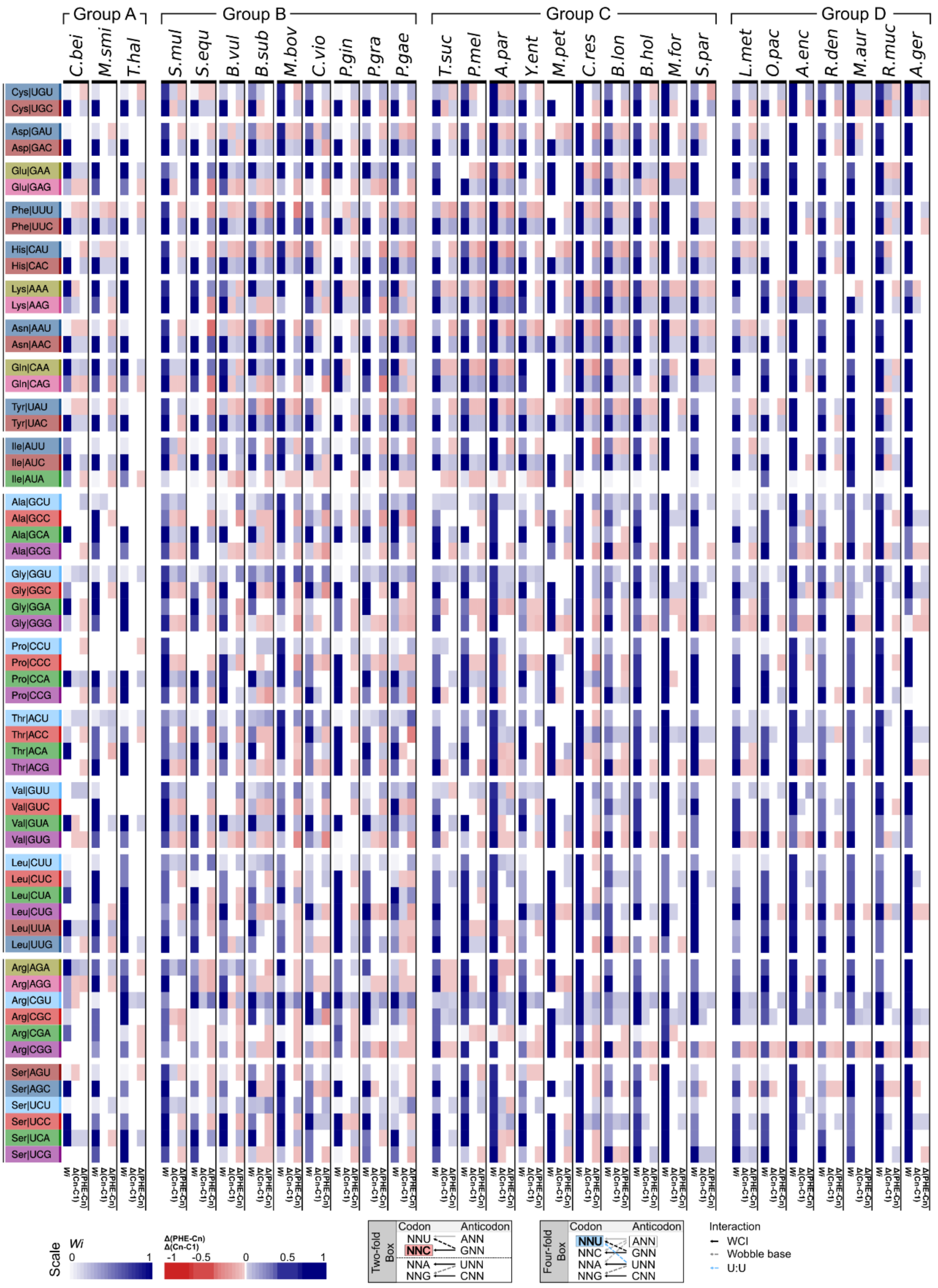

Fig. S5B. General codon-enrichment profiles in reference strains of the 29 prokaryote families analyzed. The reference species from Groups A, B, C, and D are denoted above the figure. The enrichment in a given ith codon over ancestry was

calculated as $_{\text{CODON"i"}}\text{CUF}^{\text{Cn}} - {}_{\text{CODON"i"}}\text{CUF}^{\text{C1}}$, referred to simply as Δ(Cn-C1). In like manner, the enrichment in the PHE genes over ancestry was calculated as $_{\text{CODON"i"}}\text{CUF}^{\text{PHE}} - {}_{\text{CODON"i"}}\text{CUF}^{\text{Cn}}$, similarly referred to as Δ(PHE-Cn). The rectangular boxes to the left of the figure indicating the codons in the different amino acids are color-coded according to the nature of the 3' base present as follows: blue-variants, U; red-variants, C; green-variants, A; and violet-variants G. The vertical rectangles at the left of each small panel represent the *Wi*s, with the intensity of the blue color being proportional to the values for each codon. The *Wi* color intensities are furthermore normalized to the maximum *Wi* value in each amino-acid–codon family. The vertical rectangles in the middle and at the right of each small panel represent the Δ(Cn-C1) or Δ(PHE-Cn), with the color intensity being in proportion to either an increased (blue), a decreased (red), or an equal (white) CUF in the gene sets under analysis (Cn *versus* C1, PHE *versus* Cn), as indicated in the color keys below the figure. The variations in codon-anticodon interactions (WCI, wobble base, U:U) along with their participation in the C (red) and U biases (light blue) are illustrated in the boxes below the figure.
